# Interactions with humans shape coyote responses to hazing

**DOI:** 10.1038/s41598-019-56524-6

**Published:** 2019-12-27

**Authors:** Julie K. Young, Edd Hammill, Stewart W. Breck

**Affiliations:** 1 USDA-WS-National Wildlife Research Center-Predator Research Facility, Department of Wildland Resources, Utah State University, 5230 Old Main Hill, Logan, UT 84322 USA; 2 Department of Watershed Resources, Utah State University, 5230 Old Main Hill, Logan, UT 84322 USA; 3 USDA-WS-National Wildlife Research Center, 4101 Laporte Ave., Fort Collins, CO 80521 USA

**Keywords:** Behavioural ecology, Urban ecology

## Abstract

Medium and large carnivores coexist with people in urban areas globally, occasionally resulting in negative interactions that prompt questions about how to reduce human-wildlife conflict. Hazing, i.e., scaring wildlife, is frequently promoted as an important non-lethal means for urbanites to reduce conflict but there is limited scientific evidence for its efficacy. We used a population of captive coyotes (*Canis latrans*) to simulate urban human-coyote interactions and subsequent effects of hazing on coyote behavior. Past experiences with humans significantly affected the number of times a coyote approached a human to necessitate hazing. Coyotes that had been hand fed by adults had to be more frequently hazed than coyotes with other or no past experiences with adults. Past experience with children had no impact on the number of hazing events. The number of times a coyote approached an adult or child was reduced across days based on the accumulative number of times hazed, suggesting coyotes learn to avoid behaviors warranting hazing and that this could be used as a non-lethal management tool. However, prior experience and whether the interaction is with an adult or child can alter the outcomes of hazing and must be considered in determining the efficacy of hazing programs.

## Introduction

Humans and wildlife have co-occurred throughout our shared history on Earth. Yet only in recent decades has it become common that both humans and wildlife coexist at high densities in communal environments like urban areas^1^. Coyotes (*Canis latrans*) live in cities throughout North and Central America, including all major cities in the USA^2^; leopards (*Panthera pardus*) roam India’s cities^3^; spotted hyenas (*Crocuta crocuta*) subsist on anthropogenic resources throughout urban areas of Ethiopia^4^; and Eurasian lynx (*Lynx lynx*) live amongst humans in Norway^5^. These co-occurrences between people and large carnivores reflect societal changes in human values toward carnivores, from persecution^6,7^ to protection and conservation^8^.

Coexisting with carnivores can bring new challenges related to alterations in human and carnivore behavior^9^. Humans in urban areas rarely shoot, trap, or otherwise intend to kill or harm carnivores and instead, directly and indirectly provide resources that enhance local carnivore populations^10,11^. As a result, urban carnivores can have smaller home ranges and live at higher densities^12^. They may also become bolder toward humans^13^. Thus, the consequences of coexistence can occasionally be dangerous or deadly to humans and their pets^14,15^, leading many urban governances to establish and publicize protocols on what to do if one encounters a carnivore.

With cities around the world now grappling with the issue of human-carnivore interactions, there are many examples of outreach and educational material describing what to do when a person encounters a carnivore. When aimed at urbanites, these guidelines often suggest hazing (e.g., https://www.youtube.com/watch?v=7MOnDIx71Q0). Hazing is a form of aversive conditioning that typically involves directing loud noises, chasing, and other activities that qualify as harassment of the animal. The aim of hazing is to alter the animal’s behavior or cause it to move away. While hazing can reduce undesirable behavior of wildlife (e.g.^16^,), there are no clear guidelines on how to haze carnivores or the consequences of hazing. Previous research efforts are few and have produced ambiguous results regarding the efficacy of hazing^17–19^. In general, there is a lack of science-based evidence to support hazing guidelines that could result in failures of initiatives to succeed and subsequently reduce or eliminate trust between community members and their governance^20^.

In this study, we used a population of captive coyotes and simulated scenarios between coyotes and people that commonly occur in urban settings. We then applied a hazing treatment to determine how coyotes with different human experiences subsequently responded to hazing. We focused on coyotes because hazing is often recommended as a means of reducing coyote conflict in cities around North America. Further, coyotes live in all major cities in the USA^21^, have rapidly expanded their range^22^, and urban coyotes exhibit behavioral plasticity (i.e.^11^,). Coyotes may offer the best example of complex management for urban carnivores - they are pervasive, can present a danger to human health, and interactions between coyotes and people are common throughout urban areas where both co-occur^23^.

Prior to implementing any hazing trials, we first simulated five scenarios to represent different types of interactions occurring in cities between humans and coyotes: adult walking, adult walking with a dog, adult hand-feeding coyotes and walking, child walking, and child hand-feeding coyotes and walking. We included an adult walking with a pet dog because coyote aggression toward other canids appears to be an important driver of conflict^17,24^. However, the child scenarios did not include walking a dog because of safety concerns for all three species. We included a scenario where humans were feeding coyotes because wildlife feeding is a common activity^25^, so it is likely important to understand how this human behavior affects management solutions. We included two scenarios with children because some of the more severe types of conflict involve coyotes biting children^15,26,27^ and we questioned whether coyotes could gauge the risks posed by children relative to adults. Each scenario was repeated on five pairs of adult coyotes. After exposing the pairs of coyotes to one of these scenarios for five days, we then implemented the hazing treatment. Hazing consisted of shaking a tin can full of coins, yelling, and stomping of feet at any coyote that approached within 1–3 m. This reflects behavior promoted in many cities in the USA and Canada. We also used pairs of coyotes as controls, where these coyotes did not interact with humans during the first five days but were exposed to the hazing-treatment period following this acclimatization process. We recorded the behavior of coyotes during both phases and the number of times hazed during the hazing treatment period as our response variables.

## Results

Coyotes that were hazed by children generally spent more time avoiding the child than coyotes exposed to the adult (F_6,239_ = 34.82 P = 0.043), however the actual time spent avoiding the adult or child was influenced by a series of interactive effects between whether an adult or a child was used, the treatment type, and the number of times a coyote pair was hazed. Due to these interactions, we split the data on the basis of whether an adult or a child was used, then separately investigated the effects of different treatment types and number of times hazed for each data type.

**Figure 1:**
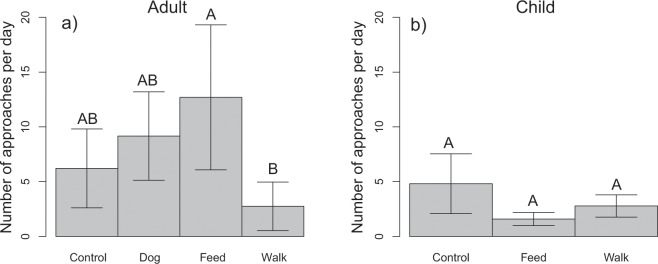
The number of times pairs of coyotes approached and were therefore hazed during the hazing-treatment period during the adult (**a**) and child (**b**) trials. Bars and error values are produced from the output of a generalized linear mixed effect model, and take into account other significant factors (i.e., cumulative previous times hazed) and random effects (coyote pair ID). Error bars denote standard errors. The same letters above bars denote treatments that are not significantly different from each other.

In the adult trials, the type of human scenario had a significant effect on the number of times a coyote approached the human subject and was subsequently hazed (F_3,70_ = 4.08, P = 0.048, Fig. 1a), with coyotes in the adult walking trials being hazed 78.3% ± 34.8% less than coyotes in the adult feeding trials. When adults were walking without a dog, coyotes approached the subject and were subsequently hazed 69.9% ± 24.1% less than when adults were walking with a dog. In addition, the number of times a coyote approached the human subject on a particular day was significantly reduced as the cumulative number of previous times hazed increased (F_1,70_ = 21.75, P < 0.001).

**Figure 2:**
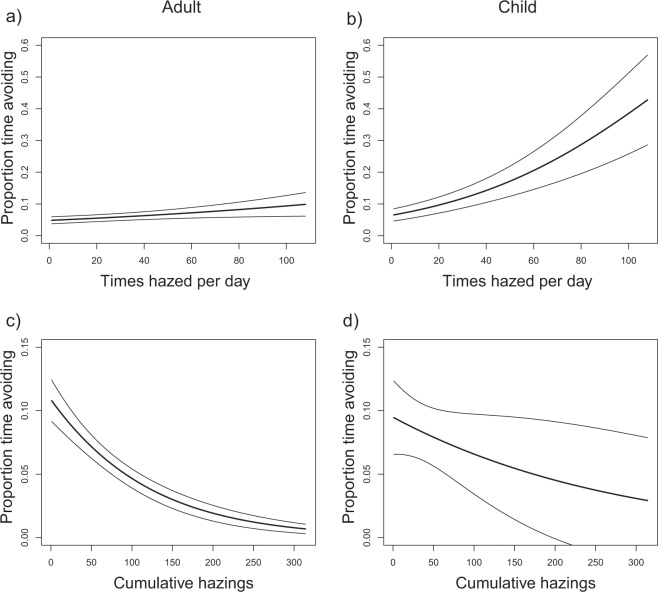
Effect of the number of daily hazings (**a**,**b**) and cumulative hazings (**c**,**d**) on the proportion of time a pair of coyotes spent avoiding the adult or child during the hazing-treatment period. Data are produced from the output of a generalized linear mixed effect model that accounts for other significant descriptive variables (treatment type) and random effects (coyote pair ID). Central lines represent the model output, narrower lines denote standard errors.

**Figure 3:**
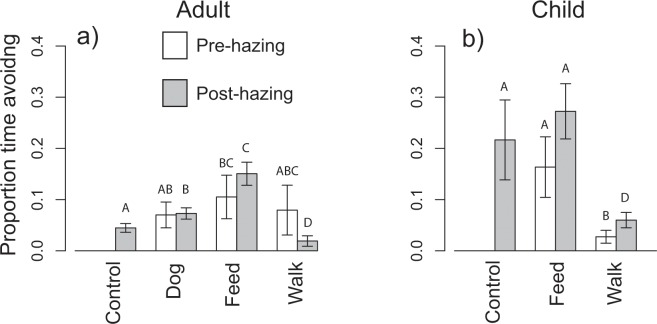
Effect of different treatments on the proportion of time coyotes spent avoiding the tester in adult (**a**) and child (**b**) trials. Bars and error values are produced from the output of a generalized linear mixed effect model, and take into account other significant fixed factors (i.e., cumulative previous hazings and number of times hazed per day) and random effects (coyote pair ID). Error bars denote standard errors. The same letters above bars denote treatments that are not significantly different from each other.

Overall for the adult trials, the time spent avoiding the adult was significantly greater during the hazing-treatment period (F_1,123_ = 3.85, P < 0.001) compared to the human-treatment period. However, there was substantial variation in the response to hazing and the opposite effect to what we predicted for some treatments. For example, following the start of hazing, the proportion of time a pair of coyotes spent avoiding the adult increased slightly with the number of times they were hazed that day (F_1,63_ = 11.44, P = 0.001, Fig. 2a); however the effect size was very small, and decreased significantly with the number of times they had been cumulatively hazed prior to that day (F_1,63_ = 86.35, P < 0.001, Fig. 2c). In addition, the type of human scenario to which the coyotes were previously exposed significantly impacted the proportion of time they spent avoiding the adult (F_3,63_ = 6.53, P < 0.001, Fig. 3a). Specifically, coyotes in the feeding scenario spent a greater proportion of time avoiding than any other treatment group, followed by the dog walking scenario, then the control animals that had no previous human scenario experience (Fig. 3a). Coyotes that were exposed to an adult-walking scenario spent less time avoiding humans than those in any other scenario (Fig. 3a). The time spent in other behavioral categories during the human-treatment and hazing-treatment periods by the type of adult-interaction scenario can be found in supplemental information (Supplementary Fig. S1a,c,e,g).

For the child trials, the type of human-interaction scenario had no significant effect on the number of times a coyote approached the human subject (F_2,55_ = 1.30, P = 0.28, Fig. 1b); however, the number of approaches per day was reduced by the number of times the coyote had been previously hazed (F_1,55_ = 23.10, P < 0.001). The proportion of time spent avoiding the child was significantly higher during the hazing-treatment period (F_1,101_ = 203.11, P < 0.001). During the hazing-treatment period, coyote pairs spent more time avoiding the child as the number of times they were hazed that day increased (F_1,51_ = 17.05, P > 0.001, Fig. 2b), but reduced the time spent avoiding the child as the number of times they had been previously hazed increased (F_1,63_ = 15.80, P < 0.001, Fig. 2d). In addition, the type of human-interaction scenario to which the coyotes were exposed significantly impacted the proportion of time they spent avoiding the child (F_2,51_ = 6.56, P < 0.001, Fig. 3b). Coyotes exposed to the control and the child-feeding scenarios spent more time avoiding a child than those exposed to a child walking scenario (Fig. 3b), however, the control and feeding scenarios were not significantly different from each other (Fig. 3b). The time spent in other behavioral categories during the human-treatment and hazing-treatment periods by the type of child-interaction scenario can be found in supplemental information (Supplementary Fig. S1b,d,f,h).

## Discussion

Until recently there has been insufficient science-based evidence on whether hazing is a useful mechanism to reduce human-carnivore conflict in urban areas^17,18^. Such studies are critical because management of urban carnivores is one of the greatest conservation challenges due to potential conflicts arising between the needs of large carnivores and risks to human health. The potential risks associated with human-carnivore conflict call for immediate actions in most cases. Therefore, there is a mismatch between the immediate needs of solving conflicts and the length of time necessary to collect sufficient data to determine the best course of action. While most local governances want to avoid human-wildlife conflict, they lack the financial, human, and time resources to do more than react to issues as they arise. Further, previous experience with humans likely shape responses of individual carnivores to hazing^28^, and historical information on individual hazing events is typically unavailable to researchers or urban managers. Thus, identifying an urban area where the risk to human health and safety is limited while providing the opportunity to experimentally test management actions and subsequent responses by carnivores is challenging to find (but see^17,18)^. Instead, creative, alternative scenarios need to be explored, such as simulating urban environments with captive carnivores to experimentally study applied questions^2^. In this study, we succeeded at using a captive population of coyotes to experimentally determine how various scenarios between humans and coyotes impact subsequent hazing attempts.

We found that prior experience with an adult (i.e., human treatment) influenced how pairs of coyotes responded to hazing. Our results indicate that coyotes are adept at learning new behavioral tactics based on previous experience. Of particular importance was our finding that the scenario of an adult hand-feeding coyotes resulted in coyotes approaching humans and subsequently being hazed more than coyotes in other human treatments (i.e., adult walking). In other words, hand-fed coyotes were more likely to continue to approach an adult even when it could result in being hazed. Although coyote pairs that were exposed to an adult walking-a-dog scenario were hazed less frequently than those exposed to an adult hand-feeding scenario, they were hazed more frequently than coyote pairs exposed to the adult-walking scenario. These differences in the number of times hazed and behavioral responses across different scenarios suggest coyotes learning to associate humans with a food resource is the strongest indicator of future human-encounter behaviors but that the presence of a dog can also be influential. Dog presence has previously been shown to increase attacks by coyotes and other carnivores^29^, a significant reduction in response of coyotes to hazing when a dog was present has previously been reported^17^, and coyotes are known to breed and interact in various ways with domestic dogs^30,31^. While in all scenarios coyotes were observed to respond by exhibiting avoidance of human behavior when hazed, our results illustrate how coyote persistence in attempting to approach a human will vary based on a combination of previous and immediate circumstances.

Results for the adult-feeding scenarios are not surprising given research findings in other systems. In approximately one-third of all cases where coyotes attacked a person, they had been previously fed by people in the vicinity of the attack^15^. Residential areas provide coyotes with year-round access to food even if people are not intentionally feeding coyotes, and both intentional and unintentional feeding ultimately result in closer proximity of coyotes to humans and higher levels of attacks^15,23,32^. Thus, our findings related to an adult feeding coyotes support the growing body of literature that show commonly used pleas for the public not to feed coyotes are warranted.

Interestingly, the results we documented in the adult scenarios were not observed when a child was involved. Instead, only the number of times coyotes had been previously hazed mattered with respect to how coyotes responded to a child. That coyotes respond differently to children may be related to the size or demeanor of children relative to adults. In this study, both children became distracted while walking and ignored the coyotes unless a hazing event was provoked, whereas the adult stayed focused on the task at hand. It could be that the size or behavior of children make them appear less threatening to coyotes or are more easily mistaken as potential prey items. Many reported coyote attacks involve children^15,23,32^ and in at least one case a series of attacks occurred at crepuscular hours on children that were rolling or running near tall grass and suspected to be mistaken as potential prey items^33^. Even so, some trends were similar between child and adult trials. For example, coyotes dramatically increased their time spent with conspecifics (i.e., their mate) during hazing, suggesting pairs interacted more with each other instead of interacting with the child or performing other behaviors and this trend was also observed, albeit weaker, with the adult trials (Supplemental Fig. S1c,d). While a behavioral shift may also be likely in the wild as a response to hazing, whether conspecific activity or an alternative behavioral state would express itself is unclear as captive coyotes may have more limited alternatives than urban coyotes. In general, like with adult trials, the coyotes in this study still appeared to have learned to reduce their proximity around a child over time, as the number of times coyotes approached children was reduced as the cumulative number of times they had been hazed increased. Thus, hazing by children may still remain effective despite differences in how coyotes respond behaviorally but further study may be warranted.

An important finding was that the number of times a pair of coyotes was hazed by an adult or child decreased over time during the hazing period. This suggests coyotes that were initially more persistent in approaching a human during the hazing-treatment period were either learning to avoid humans over time or at least learning to maintain a distance from humans that would not result in hazing. We observed coyotes moving away from the adult or child during hazing events, and this is reflected in the increased amount of time coyotes spent in avoidance behavior during the hazing-treatment period. However, the observed decrease in avoidance behavior relative to the cumulative times hazed suggests that overall coyotes may have learned to remain at distances from humans that were far enough to not trigger a hazing event. We cannot entirely rule out that coyotes recognized the fence as a barrier to interact with the adult, child, or dog by the coyotes and altered their responses over time. Coyote responses may have been influenced by the knowledge that a physical barrier existed. We do not believe it affected our results as the coyotes at the facility often interact with neighboring coyotes in areas where enclosure fence lines are in proximity to one another. Further, our responses were similar to how coyotes hazed in Colorado moved >3 m away from the person doing the hazing^17^. This is the distance is at which the coyote would no longer get hazed. Although we are unable to elucidate the mechanism entirely that altered coyote strategies to reduce the number of times hazed, the result is the same from a human health and safety standpoint: coyotes learned to remain at a distance away from humans that would allow a human to act quickly but safely to remove themselves from an encounter with a coyote. This is a promising result for managers desiring methods that reduce the potential for human-carnivore conflicts in urban areas.

Persistence of specific behaviors are often measured in problem-solving tasks as the length of time an animal is engaged working on a problem, and carnivores that show greater persistence are more likely to succeed at solving a given task^34^. Persistence can also be considered as to how long an animal engages in any specific activity^35^. In this study, coyote persistent behaviors are revealed from two types of data: the number of approaches towards humans during the hazing-treatment period and proportion of time in avoidance behavior. In both, coyotes altered behaviors in ways that showed they may have learned to avoid performing a behavior (i.e., approaching within a close distance to person) that resulted in hazing. The observed variation in behavior among and within treatment groups, however, suggests some other factors in addition to previous experience with humans may also shape the behavioral response. Coyotes exhibit behavioral syndromes, i.e., consistency in behavioral traits (aka. personalities)^36^, and this may also be important to how they respond to hazing. Alternatively, the use of captive coyote that had previously been fed by humans during normal daily care may explain the observed variation. Yet from a management perspective, limited background information regarding previous interactions with humans and behavioral traits of the coyotes are likely to be available to determine whether coyotes or other carnivores will appropriately respond to hazing. Instead, general information is likely to be available on whether a community has exposed coyotes to categories of past experiences, such as access to anthropogenic food or whether most community members have pet dogs. Based on this study, knowing the general circumstances under which coyotes have previously been exposed can help managers determine how coyotes will respond to hazing and set public expectations related to the outcomes of hazing. It is likely the same expectations about management actions based on past societal interactions with co-occurring carnivores can be made for other urban carnivores that exhibit persistent behavioral traits.

There are two caveats to our study: (1) the coyotes were housed as pairs and data were combined for analysis, so we are unable to determine if one or both individuals within each pair drove the observed patterns and (2) we could not control for whether neighbors observed each other during trials. It was not possible to clearly recognize individuals during video coding of behavior and instead conducted the analysis at the pair level. Both individuals and packs have been reported in attacks of people by coyotes^15,23,29,31–33^, so results combining pairs are still useful to broader management applications. Further, we could not observe on video whether other coyotes on trial observed and perhaps learned from their neighbors. Coyotes are able to learn via social transmission and more information is needed on this topic^37^. However, the potential to learn from observing neighbors was minimal at best for several reasons. First, the large size and configuration of the enclosures means the opportunity for coyotes to watch treatments at other enclosures would only occur for coyotes housed next to a treatment enclosure (i.e., coyotes could not watch treatments at multiple enclosures). Second, the structures in enclosures and topography create visual barriers during at least half of the treatment period (i.e., when the person was on the far side of the enclosure) thus reducing the potential for visual learning. Finally, the fact the control animals were consistent with our expectations (i.e., different than the various treatments) strongly supports the idea that visual learning was either a non-factor or relatively unimportant in our experiment. Finally, it is likely coyotes in urban areas may also be able to observe other urban coyotes and learning could also occur in this scenario. What is most important from management and human safety perspectives is whether the behavior of an approaching coyote can be altered using hazing, and our results suggest it can but with limitations to its efficacy.

Human-carnivore conflict in urban areas is increasing^29,38^, likely because high densities of both humans and carnivores in urban areas lead to increased encounter rates^1^. In urban areas, humans no longer hunt carnivores and therefore may not serve as a predator that carnivores actively avoid^39,40^. Carnivores likely relax behaviors that are considered human-fear responses and may instead be attracted to humans because of the resources they provide. Therefore, changes in human behavior may reduce the number of attacks^32^, and city managers seeking methods to reduce the risk of conflict have recently begun to promote human actions that instill fear in carnivores, such as hazing. While such actions may be warranted, there has been limited scientific evidence that the techniques being promoted are effective. Further, people in urban areas enjoy seeing wildlife and may be hesitant or ignorant of methods to ensure wildlife viewing opportunities are done safely^41^. Here, we were able to carry out experimental tests using a population of captive coyotes that exhibit behaviors typical of wild coyotes^42^ and systematically control for past experience. Thus, our study simulating interactions between coyotes and humans, with dogs, adults, children, and feeding wildlife, is both novel and important to quantifying responses to management actions aimed at reducing the risk of conflict that may subsequently occur. While this study focused on coyote-human interactions, we believe results could be relevant to management decisions aimed at reducing conflicts between mammalian carnivores and people in most urban systems.

## Methods

We simulated human-coyote interactions by using a captive population of coyotes at the USDA-National Wildlife Research Center’s (NWRC) Predator Research Facility in Millville, Utah, USA. All protocols followed humane animal care standards and were approved by NWRC’s Institute for Animal Care and Use Committee (QA-1827). The 66.4-ha facility houses ~100 adult coyotes, typically kept as male-female pairs, in outdoor enclosures. Most coyotes are born to captive parents but wild pups are brought in and hand-reared until ~10 weeks of age every few years for genetic purposes. All of the coyotes maintain behavior similar to wild counterparts^42^. They are scatter fed a wet food by animal care staff six days a week and fasted one day each week. Water is provided *ad libitum*.

During this experiment, we housed test male-female pairs of adult coyotes in eight 0.6-ha pens. Coyotes on study ranged in age from 1–10 years, with most being ≤5 years of age; we assigned different pairs of coyotes within different age classes (i.e., 1–2, 3–4, 5+) to each of the different treatments so that age was relatively equally distributed across treatment groups. The pens are octagonal shaped, with a small, roofed capture area attached to one corner. The pens are arranged in two rows of four, and coyotes may be able to observe one another in some neighboring pens. Prior to the study, each pen was fitted with two automatic feeders from which pellet food was dispensed twice daily before and during the experiments. Thus, the coyotes were not scatter fed by staff and staff did not feed or interact with the coyotes for at least six weeks before the study except to refill and check feeders. This time period was selected to be of sufficient length to habituate coyotes to the removal of human interactions but within logistical constraints of available housing and time needed to carry out the entire study. Further, the adult and children conducting the trials had never fed the coyotes previously. All coyotes continued to receive food via the automatic feeders during the trials.

We tested coyotes over two, five-day periods. The first five days, referred to as the human-treatment period, simulated human interactions with coyotes in urban areas. There was no hazing during the human-treatment period. Hazing began in the second five-day period, referred to as the hazing-treatment period. For both human- and hazing-treatment periods, the child, adult, or adult with a dog would walk around each pen, staying within approximately 0.5 m of the pen fence, at a moderate walking pace. The dog, an adult female black lab, was kept on a 150-cm long leash at all times but allowed to freely move towards and away from the fence. The adult, child, or adult with a dog walked outside of the pen for the safety of the dog, child, and coyotes. Walking took 4.5–7 minutes per pen, as some pens had more topography and the dog sometimes made stops to urinate and defecate. Walking time did not include hand-feeding, which occurred at the same location for each enclosure assigned this treatment on each of the five days before the adult or child walked around the pen. Hand-feeding typically took 1–2 minutes. For this, the person gave the pair of coyotes 12, 2.5-cm^3^ food frozen cubes of their normal wet food, dropped through or thrown over the enclosure fence prior to walking around the enclosure.

Each pair of coyotes was randomly assigned a pen, testing month, and which human treatment they would experience. Five pairs of coyotes received each treatment and none were used for more than one treatment. Because only eight pens were available, the study took place over five testing months to reach our sample sizes. The tests ran between March-October of 2012. Within each testing period, we randomly selected one pair of coyotes to serve as controls. These pairs received no human interaction during the human treatment week but the adult or child walked around the pen and performed hazing as prescribed for all test pens during the hazing-treatment period. The same adult performed all adult treatments. The female adult was 163-cm tall and Caucasian, with brown hair. Because of summer school and travel schedules, it was necessary to use two children. Each child volunteered for two consecutive weeks so that the coyotes within a testing month always interacted with the same child. Both were blonde-haired males, Caucasian, and of similar height (~144 cm) and weight at the time of testing.

After the five days of human treatment, there was no activity for two days, followed by five days of hazing treatment. No coyotes were hand fed during hazing but the dog continued to accompany the adult for all adult with dog treatments. The adult or child would walk the pen exactly as during the human treatment period, but anytime the coyote approached within <3 m, the human would haze the coyote. Hazing consisted of turning to face the coyote, shaking a small tin can that was filled with coins at it, yelling, and stomping; this is a suggested hazing technique in many urban areas^17^. The person would continue to walk after hazing, so hazing was often repeated multiple times during each day’s treatment.

While the person walked, a second person used a vehicle as a mobile blind to video record each treatment and count the number of times hazing occurred during the hazing-treatment period. Each hazing bout was defined as starting when the person first shook the tin and ending when the person started to walk again, even if only for a few steps. We used the total number of times a pair of coyotes was hazed as the hazing response variable for statistical analysis. We could not assign hazing events to a particular coyote within each pen as sometimes both coyotes were within 3 m of the person and at other times only one coyote was within 3 m and triggered a hazing event. Thus, hazing analyses were conducted at the pair level.

All videos were coded for behavior, classified into five categories (Table 1). We coded behavior for each coyote within a pair for each day but could not always determine unique identity of coyotes between days because not all have maintained their unique ear tags and the video quality was too poor to identify unique features. Thus, all behavior analyses were conducted at the pair level. To eliminate error among observers, only one person coded all video after extensive training with a second researcher to ensure consistency in behavioral coding. Training included checks for intra-observer error, to avoid drift over time. Because walking time varied, we used the proportion of time in each behavior category for statistical analysis of behavior.

**Table 1:** Description of all behavior recorded during trials simulating urban interactions between people and coyotes using pairs of captive coyotes at the NWRC Predator Research Facility. Categories used for analysis are shown.

Behavior Category	Behavior	Definition
Avoidance	Move run	Coyote runs away from the human
	Move walk	Coyote walks or trots away from the human
	Pacing	Walking, trotting, or running back and forth at a distance or away from the human
Affiliation	Approach	Coyote walks, trots, or runs toward the human
	Follow	Coyote tracks ahead or behind the human
Conspecific	Vocalize	Coyote barks, yips, and howls
	Play	Exaggerated, out of sequence, and incomplete non-aggressive actions and solicitations for action between coyotes or solicited towards dog or human
	Marking	Urinating on the ground or objects in the enclosure
	Aggression	Coyote growls, bites, or otherwise attacks other coyote
Vigilant	Sit	Coyote in a seated position while observing human, dog, or other coyotes
Other/Unknown	Still NA	Coyote stands still while observing human, dog, or other coyotes All other behavior observed or when an animal was not seen on video and behavior could not be determined

The multinomial nature of the data (coyotes were engaged in 1 of 5 exclusive behaviors at each observation) presented analytical challenges. While multinomial analyses account for the fact that a coyote may be engaged in a range of behaviors, the complex nature of these analyses makes interpretation difficult. In order to produce interpretable results where the effects of different descriptive variables could be analyzed, and because the focus of the study was to understand how different factors affect the likelihood that coyotes avoid humans, we converted our multinomial data into a binomial form and focused on one specific and biologically important behavior: whether coyotes were avoiding or not avoiding the human. This conversion to a binomial data structure allowed us to conduct the analysis using generalized linear mixed effect models (GLMM) using the lme4 package^43^ within the programming language R^44^. It also allowed for less error to be introduced from behavioral coding. All models utilized coyote pair ID as a random effect to account for the fact that the same individuals, tested as pairs, were recorded multiple times on successive days. If the initial GLMM identified a significant human treatment effect, we conducted post-hoc Tukeys tests to identify differences among treatments. The “prediceSE” function within the “AICcmodavg” package^45^ was used to isolate the effects of individual fixed factors so they could be plotted. To explore potential changes in other behavioral categories, we repeated the binomial analysis for each behavioral category and these results are provided as supplemental information.

## Supplementary information

Supplementary Information

## Data Availability

All data are available as supplementary material (Table S1) and via archives with the USDA-National Wildlife Research Center.
