# Supplementary material for: Interactions with humans shape coyote responses to hazing
Source: Sci Rep. 2019 Dec 27;9:20046. doi: 10.1038/s41598-019-56524-6 (PMC6934508; doi:10.1038/s41598-019-56524-6)
Supplement: Supplementary file 1 — Supplementary Information [file 41598_2019_56524_MOESM1_ESM.pdf]

# Interactions with humans shape coyote responses to hazing

Julie K. Young, Edd Hammil, and Stewart W. Breck

Figure S1.

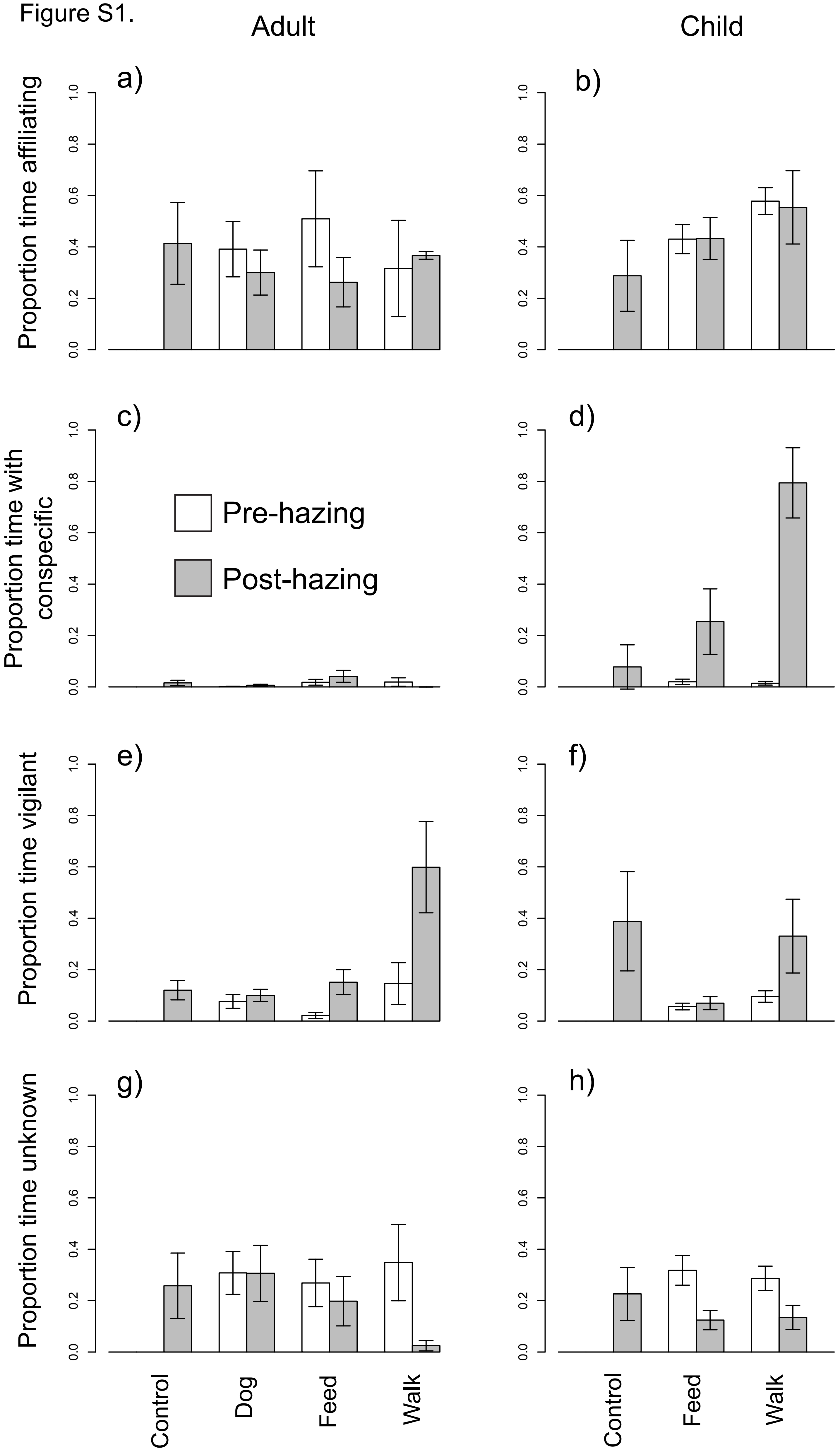

Adult
Child
a)
b)
c)
d)
e)
f)
g)
h)
Proportion time affiliating
Proportion time with conspecific
Proportion time vigilant
Proportion time unknown
Pre-hazing
Post-hazing
0.0
0.2
0.4
0.6
0.8
1.0
Control
Dog
Feed
Walk

# Table S1.

Raw Data

| Group | Month | Day | Type | Human | CoyTest | FlagTest | Coy | pen | pen.grp | grp.day.pen | uniq.ID | #TimesHaze | Avoid | Affil | Cons | Vig | unk | TOTALsec | propAvoid | PropAffil | PropCons | PropVig | PropUnk |
|---|---|---|---|---|---|---|---|---|---|---|---|---|---|---|---|---|---|---|---|---|---|---|---|
| 1 | April | 1 | Dog | adult | Bold | Bold | 1 | 1 | 1.1 | 1m1m1 | 1.1.1 | 0 | 8 | 105 | 0 | 27 | 56 | 196 | 0.0408 | 0.5357 | 0 | 0.1378 | 0.2857 |
| 1 | April | 1 | Dog | adult | Bold | Bold | 2 | 1 | 1.1 | 1m1m1 | 1.2.1 | 0 | 24 | 47 | 0 | 10 | 112 | 193 | 0.1244 | 0.2435 | 0 | 0.0518 | 0.5803 |
| 1 | April | 2 | Dog | adult | Bold | Bold | 1 | 1 | 1.1 | 1m1m2 | 1.1.1 | 0 | 39 | 100 | 3 | 42 | 24 | 208 | 0.1875 | 0.4808 | 0.0144 | 0.2019 | 0.1154 |
| 1 | April | 2 | Dog | adult | Bold | Bold | 2 | 1 | 1.1 | 1m1m2 | 1.2.1 | 0 | 15 | 166 | 2 | 20 | 5 | 208 | 0.0721 | 0.7981 | 0.0096 | 0.0962 | 0.024 |
| 1 | April | 3 | Dog | adult | Bold | Bold | 1 | 1 | 1.1 | 1m1m3 | 1.1.1 | 0 | 15 | 140 | 4 | 27 | 30 | 216 | 0.0694 | 0.6481 | 0.0185 | 0.125 | 0.1389 |
| 1 | April | 3 | Dog | adult | Bold | Bold | 2 | 1 | 1.1 | 1m1m3 | 1.2.1 | 0 | 7 | 172 | 0 | 29 | 8 | 216 | 0.0324 | 0.7963 | 0 | 0.1343 | 0.037 |
| 1 | April | 4 | Dog | adult | Bold | Bold | 1 | 1 | 1.1 | 1m1m4 | 1.1.1 | 0 | 5 | 136 | 9 | 59 | 18 | 227 | 0.022 | 0.5991 | 0.0396 | 0.2599 | 0.0793 |
| 1 | April | 4 | Dog | adult | Bold | Bold | 2 | 1 | 1.1 | 1m1m4 | 1.2.1 | 0 | 4 | 120 | 4 | 42 | 37 | 207 | 0.0193 | 0.5797 | 0.0193 | 0.2029 | 0.1787 |
| 1 | April | 5 | Dog | adult | Bold | Bold | 1 | 1 | 1.1 | 1m1m5 | 1.1.1 | 0 | 16 | 93 | 36 | 122 | 4 | 271 | 0.059 | 0.3432 | 0.1328 | 0.4502 | 0.0148 |
| 1 | April | 5 | Dog | adult | Bold | Bold | 2 | 1 | 1.1 | 1m1m5 | 1.2.1 | 0 | 12 | 132 | 7 | 76 | 44 | 271 | 0.0443 | 0.4871 | 0.0258 | 0.2804 | 0.1624 |
| 1 | April | 12 | Dog | adult | Bold | Bold | 1 | 1 | 1.1 | 1m1m12 | 1.1.1 | 0 | 80 | 57 | 0 | 41 | 110 | 288 | 0.2778 | 0.1979 | 0 | 0.1424 | 0.3819 |
| 1 | April | 1 | Dog | adult | Shy | Shy | 1 | 2 | 1.2 | 1m2m1 | 1.1.2 | 0 | 6 | 10 | 14 | 0 | 100 | 130 | 0.0462 | 0.0769 | 0.1077 | 0 | 0.7692 |
| 1 | April | 1 | Dog | adult | Shy | Shy | 2 | 2 | 1.2 | 1m2m1 | 1.2.2 | 0 | 3 | 0 | 8 | 0 | 119 | 130 | 0.0231 | 0 | 0.0615 | 0 | 0.9154 |
| 1 | April | 2 | Dog | adult | Shy | Shy | 1 | 2 | 1.2 | 1m2m2 | 1.1.2 | 0 | 16 | 55 | 0 | 19 | 121 | 211 | 0.0758 | 0.2607 | 0 | 0.09 | 0.5735 |
| 1 | April | 2 | Dog | adult | Shy | Shy | 2 | 2 | 1.2 | 1m2m2 | 1.2.2 | 0 | 3 | 49 | 0 | 23 | 136 | 211 | 0.0142 | 0.2322 | 0 | 0.109 | 0.6445 |
| 1 | April | 3 | Dog | adult | Shy | Shy | 1 | 2 | 1.2 | 1m2m3 | 1.1.2 | 0 | 15 | 116 | 0 | 21 | 13 | 165 | 0.0909 | 0.703 | 0 | 0.1273 | 0.0788 |
| 1 | April | 3 | Dog | adult | Shy | Shy | 2 | 2 | 1.2 | 1m2m3 | 1.2.2 | 0 | 29 | 34 | 0 | 24 | 78 | 165 | 0.1758 | 0.2061 | 0 | 0.1455 | 0.4727 |
| 1 | April | 4 | Dog | adult | Shy | Shy | 1 | 2 | 1.2 | 1m2m4 | 1.1.2 | 0 | 5 | 69 | 0 | 59 | 17 | 150 | 0.0333 | 0.46 | 0 | 0.3933 | 0.1133 |
| 1 | April | 4 | Dog | adult | Shy | Shy | 2 | 2 | 1.2 | 1m2m4 | 1.2.2 | 0 | 30 | 48 | 0 | 11 | 61 | 150 | 0.2 | 0.32 | 0 | 0.0733 | 0.4067 |
| 1 | April | 5 | Dog | adult | Shy | Shy | 1 | 2 | 1.2 | 1m2m5 | 1.1.2 | 0 | 11 | 110 | 0 | 30 | 46 | 197 | 0.0558 | 0.5584 | 0 | 0.1523 | 0.2335 |
| 1 | April | 5 | Dog | adult | Shy | Shy | 2 | 2 | 1.2 | 1m2m5 | 1.2.2 | 0 | 12 | 33 | 0 | 41 | 111 | 197 | 0.0609 | 0.1675 | 0 | 0.2081 | 0.5635 |
| 1 | April | 8 | Dog | adult | Shy | Shy | 1 | 2 | 1.2 | 1m2m8 | 1.1.2 | 0 | 23 | 40 | 0 | 28 | 102 | 193 | 0.1192 | 0.2073 | 0 | 0.1451 | 0.5285 |
| 1 | April | 9 | Dog | adult | Shy | Shy | 1 | 2 | 1.2 | 1m2m9 | 1.1.2 | 0 | 28 | 75 | 0 | 26 | 88 | 217 | 0.129 | 0.3456 | 0 | 0.1198 | 0.4055 |
| 1 | April | 11 | Dog | adult | Shy | Shy | 1 | 2 | 1.2 | 1m2m11 | 1.1.2 | 0 | 16 | 49 | 0 | 48 | 111 | 224 | 0.0714 | 0.2188 | 0 | 0.2143 | 0.4955 |
| 1 | April | 1 | Feed | adult | Shy | Shy | 1 | 3 | 1.3 | 1m3m1 | 1.1.3 | 0 | 23 | 0 | 0 | 0 | 211 | 234 | 0.0983 | 0 | 0 | 0 | 0.9017 |
| 1 | April | 1 | Feed | adult | Shy | Shy | 2 | 3 | 1.3 | 1m3m1 | 1.2.3 | 0 | 30 | 0 | 0 | 0 | 206 | 236 | 0.1271 | 0 | 0 | 0 | 0.8729 |
| 1 | April | 2 | Feed | adult | Shy | Shy | 1 | 3 | 1.3 | 1m3m2 | 1.1.3 | 0 | 52 | 0 | 0 | 16 | 221 | 289 | 0.1799 | 0 | 0 | 0.0554 | 0.7647 |
| 1 | April | 2 | Feed | adult | Shy | Shy | 2 | 3 | 1.3 | 1m3m2 | 1.2.3 | 0 | 97 | 18 | 0 | 9 | 165 | 289 | 0.3356 | 0.0623 | 0 | 0.0311 | 0.5709 |
| 1 | April | 3 | Feed | adult | Shy | Shy | 1 | 3 | 1.3 | 1m3m3 | 1.1.3 | 0 | 65 | 24 | 0 | 6 | 172 | 267 | 0.2434 | 0.0899 | 0 | 0.0225 | 0.6442 |
| 1 | April | 3 | Feed | adult | Shy | Shy | 2 | 3 | 1.3 | 1m3m3 | 1.2.3 | 0 | 95 | 17 | 9 | 10 | 136 | 267 | 0.3558 | 0.0637 | 0.0337 | 0.0375 | 0.5094 |
| 1 | April | 4 | Feed | adult | Shy | Shy | 1 | 3 | 1.3 | 1m3m4 | 1.1.3 | 0 | 127 | 0 | 0 | 3 | 139 | 269 | 0.4721 | 0 | 0 | 0.0112 | 0.5167 |
| 1 | April | 4 | Feed | adult | Shy | Shy | 2 | 3 | 1.3 | 1m3m4 | 1.2.3 | 0 | 90 | 0 | 0 | 27 | 152 | 269 | 0.3346 | 0 | 0 | 0.1004 | 0.5651 |
| 1 | April | 5 | Feed | adult | Shy | Shy | 1 | 3 | 1.3 | 1m3m5 | 1.1.3 | 0 | 160 | 10 | 0 | 64 | 108 | 342 | 0.4678 | 0.0292 | 0 | 0.1871 | 0.3158 |
| 1 | April | 5 | Feed | adult | Shy | Shy | 2 | 3 | 1.3 | 1m3m5 | 1.2.3 | 0 | 120 | 28 | 0 | 80 | 114 | 342 | 0.3509 | 0.0819 | 0 | 0.2339 | 0.3333 |
| 1 | April | 8 | Feed | adult | Shy | Shy | 1 | 3 | 1.3 | 1m3m8 | 1.1.3 | 0 | 132 | 11 | 0 | 25 | 87 | 255 | 0.5176 | 0.0431 | 0 | 0.098 | 0.3412 |
| 1 | April | 8 | Feed | adult | Shy | Shy | 2 | 3 | 1.3 | 1m3m8 | 1.2.3 | 0 | 140 | 0 | 0 | 47 | 68 | 255 | 0.549 | 0 | 0 | 0.1843 | 0.2667 |
| 1 | April | 9 | Feed | adult | Shy | Shy | 1 | 3 | 1.3 | 1m3m9 | 1.1.3 | 0 | 115 | 0 | 0 | 7 | 112 | 234 | 0.4915 | 0 | 0 | 0.0299 | 0.4786 |
| 1 | April | 9 | Feed | adult | Shy | Shy | 2 | 3 | 1.3 | 1m3m9 | 1.2.3 | 0 | 91 | 0 | 0 | 0 | 143 | 234 | 0.3889 | 0 | 0 | 0 | 0.6111 |
| 1 | April | 10 | Feed | adult | Shy | Shy | 1 | 3 | 1.3 | 1m3m10 | 1.1.3 | 0 | 203 | 0 | 0 | 19 | 28 | 250 | 0.812 | 0 | 0 | 0.076 | 0.112 |
| 1 | April | 10 | Feed | adult | Shy | Shy | 2 | 3 | 1.3 | 1m3m10 | 1.2.3 | 0 | 197 | 0 | 0 | 10 | 43 | 250 | 0.788 | 0 | 0 | 0.04 | 0.172 |
| 1 | April | 11 | Feed | adult | Shy | Shy | 1 | 3 | 1.3 | 1m3m11 | 1.1.3 | 0 | 181 | 0 | 0 | 45 | 62 | 288 | 0.6285 | 0 | 0 | 0.1563 | 0.2153 |
| 1 | April | 11 | Feed | adult | Shy | Shy | 2 | 3 | 1.3 | 1m3m11 | 1.2.3 | 0 | 192 | 0 | 0 | 38 | 56 | 286 | 0.6713 | 0 | 0 | 0.1329 | 0.1958 |
| 1 | April | 12 | Feed | adult | Shy | Shy | 1 | 3 | 1.3 | 1m3m12 | 1.1.3 | 0 | 104 | 0 | 0 | 3 | 128 | 235 | 0.4426 | 0 | 0 | 0.0128 | 0.5447 |
| 1 | April | 12 | Feed | adult | Shy | Shy | 2 | 3 | 1.3 | 1m3m12 | 1.2.3 | 0 | 130 | 0 | 0 | 15 | 90 | 235 | 0.5532 | 0 | 0 | 0.0638 | 0.383 |
| 1 | April | 1 | Feed | adult | Bold | Bold | 1 | 4 | 1.4 | 1m4m1 | 1.1.4 | 0 | 6 | 126 | 11 | 5 | 9 | 157 | 0.0382 | 0.8025 | 0.0701 | 0.0318 | 0.0573 |
| 1 | April | 1 | Feed | adult | Bold | Bold | 2 | 4 | 1.4 | 1m4m1 | 1.2.4 | 0 | 38 | 72 | 0 | 0 | 153 | 263 | 0.1445 | 0.2738 | 0 | 0 | 0.5817 |
| 1 | April | 2 | Feed | adult | Bold | Bold | 1 | 4 | 1.4 | 1m4m2 | 1.1.4 | 0 | 19 | 148 | 0 | 0 | 0 | 167 | 0.1138 | 0.8862 | 0 | 0 | 0 |
| 1 | April | 2 | Feed | adult | Bold | Bold | 2 | 4 | 1.4 | 1m4m2 | 1.2.4 | 0 | 33 | 68 | 12 | 8 | 142 | 263 | 0.1255 | 0.2586 | 0.0456 | 0.0304 | 0.5399 |
| 1 | April | 3 | Feed | adult | Bold | Bold | 1 | 4 | 1.4 | 1m4m3 | 1.1.4 | 0 | 42 | 133 | 0 | 0 | 44 | 219 | 0.1918 | 0.6073 | 0 | 0 | 0.2009 |
| 1 | April | 3 | Feed | adult | Bold | Bold | 2 | 4 | 1.4 | 1m4m3 | 1.2.4 | 0 | 37 | 137 | 0 | 14 | 49 | 237 | 0.1561 | 0.5781 | 0 | 0.0591 | 0.2068 |
| 1 | April | 4 | Feed | adult | Bold | Bold | 1 | 4 | 1.4 | 1m4m4 | 1.1.4 | 0 | 24 | 131 | 2 | 5 | 20 | 182 | 0.1319 | 0.7198 | 0.011 | 0.0275 | 0.1099 |
| 1 | April | 4 | Feed | adult | Bold | Bold | 2 | 4 | 1.4 | 1m4m4 | 1.2.4 | 0 | 52 | 101 | 0 | 10 | 23 | 186 | 0.2796 | 0.543 | 0 | 0.0538 | 0.1237 |
| 1 | April | 5 | Feed | adult | Bold | Bold | 1 | 4 | 1.4 | 1m4m5 | 1.1.4 | 0 | 40 | 46 | 2 | 10 | 98 | 196 | 0.2041 | 0.2347 | 0.0102 | 0.051 | 0.5 |
| 1 | April | 5 | Feed | adult | Bold | Bold | 2 | 4 | 1.4 | 1m4m5 | 1.2.4 | 0 | 30 | 20 | 0 | 0 | 93 | 143 | 0.2098 | 0.1399 | 0 | 0 | 0.6503 |
| 1 | April | 8 | Feed | adult | Bold | Bold | 1 | 4 | 1.4 | 1m4m8 | 1.1.4 | 0 | 39 | 34 | 16 | 10 | 170 | 269 | 0.145 | 0.1264 | 0.0595 | 0.0372 | 0.632 |
| 1 | April | 10 | Feed | adult | Bold | Bold | 2 | 4 | 1.4 | 1m4m10 | 1.2.4 | 0 | 3 | 86 | 6 | 23 | 128 | 246 | 0.0122 | 0.3496 | 0.0244 | 0.0935 | 0.5203 |
| 1 | April | 12 | Feed | adult | Bold | Bold | 1 | 4 | 1.4 | 1m4m12 | 1.1.4 | 0 | 44 | 21 | 4 | 24 | 172 | 265 | 0.166 | 0.0792 | 0.0151 | 0.0906 | 0.6491 |
| 1 | April | 12 | Feed | adult | Bold | Bold | 2 | 4 | 1.4 | 1m4m12 | 1.2.4 | 0 | 51 | 30 | 0 | 42 | 142 | 265 | 0.1925 | 0.1132 | 0 | 0.1585 | 0.5358 |
| 1 | April | 1 | Walk | adult | Bold | Bold | 1 | 5 | 1.5 | 1m5m1 | 1.1.5 | 0 | 8 | 0 | 0 | 0 | 223 | 231 | 0.0346 | 0 | 0 | 0 | 0.9654 |
| 1 | April | 1 | Walk | adult | Bold | Bold | 2 | 5 | 1.5 | 1m5m1 | 1.2.5 | 0 | 51 | 0 | 0 | 0 | 180 | 231 | 0.2208 | 0 | 0 | 0 | 0.7792 |
| 1 | April | 2 | Walk | adult | Bold | Bold | 1 | 5 | 1.5 | 1m5m2 | 1.1.5 | 0 | 25 | 13 | 0 | 11 | 151 | 200 | 0.125 | 0.065 | 0 | 0.055 | 0.755 |
| 1 | April | 2 | Walk | adult | Bold | Bold | 2 | 5 | 1.5 | 1m5m2 | 1.2.5 | 0 | 20 | 18 | 0 | 16 | 146 | 200 | 0.1 | 0.09 | 0 | 0.08 | 0.73 |
| 1 | April | 3 | Walk | adult | Bold | Bold | 1 | 5 | 1.5 | 1m5m3 | 1.1.5 | 0 | 38 | 0 | 0 | 0 | 184 | 222 | 0.1712 | 0 | 0 | 0 | 0.8288 |
| 1 | April | 3 | Walk | adult | Bold | Bold | 2 | 5 | 1.5 | 1m5m3 | 1.2.5 | 0 | 34 | 0 | 0 | 24 | 164 | 222 | 0.1532 | 0 | 0 | 0.1081 | 0.7387 |
| 1 | April | 4 | Walk | adult | Bold | Bold | 1 | 5 | 1.5 | 1m5m4 | 1.1.5 | 0 | 77 | 23 | 0 | 27 | 126 | 253 | 0.3043 | 0.0909 | 0 | 0.1067 | 0.498 |
| 1 | April | 4 | Walk | adult | Bold | Bold | 2 | 5 | 1.5 | 1m5m4 | 1.2.5 | 0 | 49 | 49 | 9 | 14 | 132 | 253 | 0.1937 | 0.1937 | 0.0356 | 0.0553 | 0.5217 |
| 1 | April | 5 | Walk | adult | Bold | Bold | 1 | 5 | 1.5 | 1m5m5 | 1.1.5 | 0 | 35 | 58 | 0 | 182 | 16 | 291 | 0.1203 | 0.1993 | 0 | 0.6254 | 0.055 |
| 1 | April | 5 | Walk | adult | Bold | Bold | 2 | 5 | 1.5 | 1m5m5 | 1.2.5 | 0 | 33 | 42 | 0 | 116 | 100 | 291 | 0.1134 | 0.1443 | 0 | 0.3986 | 0.3436 |
| 1 | April | 8 | Walk | adult | Bold | Bold | 1 | 5 | 1.5 | 1m5m8 | 1.1.5 | 0 | 131 | 5 | 0 | 20 | 100 | 256 | 0.5117 | 0.0195 | 0 | 0.0781 | 0.3906 |
| 1 | April | 8 | Walk | adult | Bold | Bold | 2 | 5 | 1.5 | 1m5m8 | 1.2.5 | 0 | 77 | 10 | 0 | 61 | 108 | 256 | 0.3008 | 0.0391 | 0 | 0.2383 | 0.4219 |
| 1 | April | 9 | Walk | adult | Bold | Bold | 1 | 5 | 1.5 | 1m5m9 | 1.1.5 | 0 | 56 | 8 | 0 | 4 | 173 | 241 | 0.2324 | 0.0332 | 0 | 0.0166 | 0.7178 |
| 1 | April | 9 | Walk | adult | Bold | Bold | 2 | 5 | 1.5 | 1m5m9 | 1.2.5 | 0 | 70 | 52 | 3 | 28 | 88 | 241 | 0.2905 | 0.2158 | 0.0124 | 0.1162 | 0.3651 |
| 1 | April | 10 | Walk | adult | Bold | Bold | 1 | 5 | 1.5 | 1m5m10 | 1.1.5 | 0 | 69 | 25 | 5 | 12 | 123 | 234 | 0.2949 | 0.1068 | 0.0214 | 0.0513 | 0.5256 |
| 1 | April | 10 | Walk | adult | Bold | Bold | 2 | 5 | 1.5 | 1m5m10 | 1.2.5 | 0 | 27 | 45 | 0 | 27 | 135 | 234 | 0.1154 | 0.1923 | 0 | 0.1154 | 0.5769 |
| 1 | April | 11 | Walk | adult | Bold | Bold | 1 | 5 | 1.5 | 1m5m11 | 1.1.5 | 0 | 91 | 0 | 35 | 22 | 115 | 263 | 0.346 | 0 | 0.1331 | 0.0837 | 0.4373 |
| 1 | April | 11 | Walk | adult | Bold | Bold | 2 | 5 | 1.5 | 1m5m11 | 1.2.5 | 0 | 73 | 24 | 29 | 54 | 83 | 263 | 0.2776 | 0.0913 | 0.1103 | 0.2053 | 0.3156 |
| 1 | April | 12 | Walk | adult | Bold | Bold | 1 | 5 | 1.5 | 1m5m12 | 1.1.5 | 0 | 144 | 0 | 0 | 74 | 339 | 557 | 0.2585 | 0 | 0 | 0.1329 | 0.6086 |
| 1 | April | 12 | Walk | adult | Bold | Bold | 2 | 5 | 1.5 | 1m5m12 | 1.2.5 | 0 | 283 | 25 | 1 | 40 | 208 | 557 | 0.5081 | 0.0449 | 0.0018 | 0.0718 | 0.3734 |
| 1 | April | 1 | Walk | adult | Bold | Bold | 1 | 7 | 1.7 | 1m7m1 | 1.1.7 | 0 | 18 | 173 | 18 | 27 | 5 | 241 | 0.0747 | 0.7178 | 0.0747 | 0.112 | 0.0207 |
| 1 | April | 1 | Walk | adult | Bold | Bold | 2 | 7 | 1.7 | 1m7m1 | 1.2.7 | 0 | 17 | 35 | 0 | 29 | 160 | 241 | 0.0705 | 0.1452 | 0 | 0.1203 | 0.6639 |
| 1 | April | 2 | Walk | adult | Bold | Bold | 1 | 7 | 1.7 | 1m7m2 | 1.1.7 | 0 | 3 | 129 | 0 | 70 | 18 | 220 | 0.0136 | 0.5864 | 0 | 0.3182 | 0.0818 |
| 1 | April | 2 | Walk | adult | Bold | Bold | 2 | 7 | 1.7 | 1m7m2 | 1.2.7 | 0 | 3 | 0 | 0 | 20 | 197 | 220 | 0.0136 | 0 | 0 | 0.0909 | 0.8955 |
| 1 | April | 3 | Walk | adult | Bold | Bold | 1 | 7 | 1.7 | 1m7m3 | 1.1.7 | 0 | 38 | 88 | 25 | 37 | 16 | 204 | 0.1863 | 0.4314 | 0.1225 | 0.1814 | 0.0784 |
| 1 | April | 3 | Walk | adult | Bold | Bold | 2 | 7 | 1.7 | 1m7m3 | 1.2.7 | 0 | 0 | 0 | 0 | 64 | 140 | 204 | 0 | 0 | 0 | 0.3137 | 0.6863 |
| 1 | April | 4 | Walk | adult | Bold | Bold | 1 | 7 | 1.7 | 1m7m4 | 1.1.7 | 0 | 18 | 114 | 10 | 65 | 0 | 207 | 0.087 | 0.5507 | 0.0483 | 0.314 | 0 |
| 1 | April | 4 | Walk | adult | Bold | Bold | 2 | 7 | 1.7 | 1m7m4 | 1.2.7 | 0 | 0 | 0 | 10 | 197 | 0 | 207 | 0 | 0 | 0.0483 | 0.9517 | 0 |
| 1 | April | 5 | Walk | adult | Bold | Bold | 1 | 7 | 1.7 | 1m7m5 | 1.1.7 | 0 | 46 | 110 | 0 | 86 | 51 | 293 | 0.157 | 0.3754 | 0 | 0.2935 | 0.1741 |
| 1 | April | 5 | Walk | adult | Bold | Bold | 2 | 7 | 1.7 | 1m7m5 | 1.2.7 | 0 | 0 | 0 | 0 | 293 | 0 | 293 | 0 | 0 | 0 | 1 | 0 |
| 1 | April | 8 | Walk | adult | Bold | Bold | 2 | 7 | 1.7 | 1m7m8 | 1.2.7 | 0 | 4 | 96 | 0 | 60 | 15 | 175 | 0.0229 | 0.5486 | 0 | 0.3429 | 0.0857 |
| 1 | April | 9 | Walk | adult | Bold | Bold | 2 | 7 | 1.7 | 1m7m9 | 1.2.7 | 0 | 14 | 0 | 0 | 17 | 202 | 233 | 0.0601 | 0 | 0 | 0.073 | 0.867 |
| 1 | April | 10 | Walk | adult | Bold | Bold | 2 | 7 | 1.7 | 1m7m10 | 1.2.7 | 0 | 4 | 0 | 0 | 32 | 205 | 241 | 0.0166 | 0 | 0 | 0.1328 | 0.8506 |

| | | | | | | | | | | | | | | | | | | | | | | | |
|---|---|---|---|---|---|---|---|---|---|---|---|---|---|---|---|---|---|---|---|---|---|---|---|
| 1 | April | 11 | Walk | adult | Bold | Bold | 1 | 7 | 1.7 | 1m7m11 | 1.1.7 | 0 | 0 | 0 | 0 | 63 | 177 | 240 | 0 | 0 | 0 | 0.2625 | 0.7375 |
| 1 | April | 12 | Walk | adult | Bold | Bold | 1 | 7 | 1.7 | 1m7m12 | 1.1.7 | 0 | 24 | 0 | 0 | 26 | 170 | 220 | 0.1091 | 0 | 0 | 0.1182 | 0.7727 |
| 1 | April | 12 | Walk | adult | Bold | Bold | 2 | 7 | 1.7 | 1m7m12 | 1.2.7 | 0 | 38 | 2 | 0 | 23 | 157 | 220 | 0.1727 | 0.0091 | 0 | 0.1045 | 0.7136 |
| 1 | April | 8 | aControl | adult | Shy | Shy | 2 | 8 | 1.8 | 1m8m8 | 1.2.8 | 0 | 0 | 41 | 0 | 13 | 189 | 243 | 0 | 0.1687 | 0 | 0.0535 | 0.7778 |
| 1 | April | 9 | aControl | adult | Shy | Shy | 2 | 8 | 1.8 | 1m8m9 | 1.2.8 | 0 | 0 | 55 | 0 | 13 | 177 | 245 | 0 | 0.2245 | 0 | 0.0531 | 0.7224 |
| 1 | April | 10 | aControl | adult | Shy | Shy | 2 | 8 | 1.8 | 1m8m10 | 1.2.8 | 0 | 14 | 40 | 0 | 10 | 175 | 239 | 0.0586 | 0.1674 | 0 | 0.0418 | 0.7322 |
| 1 | April | 12 | aControl | adult | Shy | Shy | 1 | 8 | 1.8 | 1m8m12 | 1.1.8 | 0 | 27 | 0 | 0 | 65 | 136 | 228 | 0.1184 | 0 | 0 | 0.2851 | 0.5965 |
| 2 | June | 1 | ChildFeed | child | Bold | Bold | 1 | 1 | 2.1 | 2m1m1 | 2.1.1 | 0 | 18 | 48 | 0 | 18 | 149 | 233 | 0.0773 | 0.206 | 0 | 0.0773 | 0.6395 |
| 2 | June | 1 | ChildFeed | child | Bold | Bold | 2 | 1 | 2.1 | 2m1m1 | 2.2.1 | 0 | 32 | 81 | 0 | 24 | 96 | 233 | 0.1373 | 0.3476 | 0 | 0.103 | 0.412 |
| 2 | June | 2 | ChildFeed | child | Bold | Bold | 1 | 1 | 2.1 | 2m1m2 | 2.1.1 | 0 | 70 | 59 | 0 | 0 | 58 | 187 | 0.3743 | 0.3155 | 0 | 0 | 0.3102 |
| 2 | June | 2 | ChildFeed | child | Bold | Bold | 2 | 1 | 2.1 | 2m1m2 | 2.2.1 | 0 | 123 | 54 | 0 | 0 | 53 | 230 | 0.5348 | 0.2348 | 0 | 0 | 0.2304 |
| 2 | June | 3 | ChildFeed | child | Bold | Bold | 1 | 1 | 2.1 | 2m1m3 | 2.1.1 | 0 | 33 | 56 | 11 | 2 | 41 | 143 | 0.2308 | 0.3916 | 0.0769 | 0.014 | 0.2867 |
| 2 | June | 3 | ChildFeed | child | Bold | Bold | 2 | 1 | 2.1 | 2m1m3 | 2.2.1 | 0 | 36 | 145 | 0 | 0 | 6 | 187 | 0.1925 | 0.7754 | 0 | 0 | 0.0321 |
| 2 | June | 4 | ChildFeed | child | Bold | Bold | 1 | 1 | 2.1 | 2m1m4 | 2.1.1 | 0 | 45 | 91 | 0 | 4 | 20 | 160 | 0.2813 | 0.5688 | 0 | 0.025 | 0.125 |
| 2 | June | 4 | ChildFeed | child | Bold | Bold | 2 | 1 | 2.1 | 2m1m4 | 2.2.1 | 0 | 15 | 66 | 0 | 11 | 52 | 144 | 0.1042 | 0.4583 | 0 | 0.0764 | 0.3611 |
| 2 | June | 5 | ChildFeed | child | Bold | Bold | 1 | 1 | 2.1 | 2m1m5 | 2.1.1 | 0 | 23 | 46 | 12 | 6 | 61 | 148 | 0.1554 | 0.3108 | 0.0811 | 0.0405 | 0.4122 |
| 2 | June | 5 | ChildFeed | child | Bold | Bold | 2 | 1 | 2.1 | 2m1m5 | 2.2.1 | 0 | 48 | 82 | 0 | 0 | 0 | 130 | 0.3692 | 0.6308 | 0 | 0 | 0 |
| 2 | June | 9 | ChildFeed | child | Bold | Bold | 1 | 1 | 2.1 | 2m1m9 | 2.1.1 | 0 | 18 | 116 | 11 | 80 | 0 | 225 | 0.08 | 0.5156 | 0.0489 | 0.3556 | 0 |
| 2 | June | 9 | ChildFeed | child | Bold | Bold | 2 | 1 | 2.1 | 2m1m9 | 2.2.1 | 0 | 38 | 40 | 12 | 90 | 45 | 225 | 0.1689 | 0.1778 | 0.0533 | 0.4 | 0.2 |
| 2 | June | 10 | ChildFeed | child | Bold | Bold | 1 | 1 | 2.1 | 2m1m10 | 2.1.1 | 0 | 95 | 33 | 10 | 73 | 17 | 228 | 0.4167 | 0.1447 | 0.0439 | 0.3202 | 0.0746 |
| 2 | June | 10 | ChildFeed | child | Bold | Bold | 2 | 1 | 2.1 | 2m1m10 | 2.2.1 | 0 | 120 | 10 | 13 | 85 | 0 | 228 | 0.5263 | 0.0439 | 0.057 | 0.3728 | 0 |
| 2 | June | 12 | ChildFeed | child | Bold | Bold | 1 | 1 | 2.1 | 2m1m12 | 2.1.1 | 0 | 0 | 70 | 70 | 23 | 0 | 163 | 0 | 0.4294 | 0.4294 | 0.1411 | 0 |
| 2 | June | 12 | ChildFeed | child | Bold | Bold | 2 | 1 | 2.1 | 2m1m12 | 2.2.1 | 0 | 8 | 51 | 73 | 31 | 0 | 163 | 0.0491 | 0.3129 | 0.4479 | 0.1902 | 0 |
| 2 | June | 1 | ChildFeed | child | Bold | Bold | 1 | 2 | 2.2 | 2m2m1 | 2.1.2 | 0 | 59 | 141 | 0 | 0 | 26 | 226 | 0.2611 | 0.6239 | 0 | 0 | 0.115 |
| 2 | June | 1 | ChildFeed | child | Bold | Bold | 2 | 2 | 2.2 | 2m2m1 | 2.2.2 | 0 | 0 | 90 | 2 | 0 | 85 | 177 | 0 | 0.5085 | 0.0113 | 0 | 0.4802 |
| 2 | June | 2 | ChildFeed | child | Bold | Bold | 1 | 2 | 2.2 | 2m2m2 | 2.1.2 | 0 | 63 | 90 | 0 | 0 | 19 | 172 | 0.3663 | 0.5233 | 0 | 0 | 0.1105 |
| 2 | June | 2 | ChildFeed | child | Bold | Bold | 2 | 2 | 2.2 | 2m2m2 | 2.2.2 | 0 | 15 | 2 | 0 | 35 | 110 | 162 | 0.0926 | 0.0123 | 0 | 0.216 | 0.679 |
| 2 | June | 3 | ChildFeed | child | Bold | Bold | 1 | 2 | 2.2 | 2m2m3 | 2.1.2 | 0 | 16 | 110 | 0 | 0 | 19 | 145 | 0.1103 | 0.7586 | 0 | 0 | 0.131 |
| 2 | June | 3 | ChildFeed | child | Bold | Bold | 2 | 2 | 2.2 | 2m2m3 | 2.2.2 | 0 | 0 | 16 | 6 | 24 | 68 | 114 | 0 | 0.1404 | 0.0526 | 0.2105 | 0.5965 |
| 2 | June | 4 | ChildFeed | child | Bold | Bold | 1 | 2 | 2.2 | 2m2m4 | 2.1.2 | 0 | 30 | 112 | 0 | 0 | 0 | 142 | 0.2113 | 0.7887 | 0 | 0 | 0 |
| 2 | June | 4 | ChildFeed | child | Bold | Bold | 2 | 2 | 2.2 | 2m2m4 | 2.2.2 | 0 | 12 | 69 | 6 | 29 | 38 | 154 | 0.0779 | 0.4481 | 0.039 | 0.1883 | 0.2468 |
| 2 | June | 5 | ChildFeed | child | Bold | Bold | 1 | 2 | 2.2 | 2m2m5 | 2.1.2 | 0 | 0 | 0 | 13 | 6 | 56 | 75 | 0 | 0 | 0.1733 | 0.08 | 0.7467 |
| 2 | June | 5 | ChildFeed | child | Bold | Bold | 2 | 2 | 2.2 | 2m2m5 | 2.2.2 | 0 | 8 | 50 | 9 | 0 | 0 | 67 | 0.1194 | 0.7463 | 0.1343 | 0 | 0 |
| 2 | June | 9 | ChildFeed | child | Bold | Bold | 1 | 2 | 2.2 | 2m2m9 | 2.1.2 | 0 | 0 | 8 | 0 | 114 | 107 | 229 | 0 | 0.0349 | 0 | 0.4978 | 0.4672 |
| 2 | June | 9 | ChildFeed | child | Bold | Bold | 2 | 2 | 2.2 | 2m2m9 | 2.2.2 | 0 | 68 | 74 | 0 | 87 | 0 | 229 | 0.2969 | 0.3231 | 0 | 0.3799 | 0 |
| 2 | June | 12 | ChildFeed | child | Bold | Bold | 1 | 2 | 2.2 | 2m2m12 | 2.1.2 | 0 | 33 | 41 | 10 | 95 | 0 | 179 | 0.1844 | 0.2291 | 0.0559 | 0.5307 | 0 |
| 2 | June | 12 | ChildFeed | child | Bold | Bold | 2 | 2 | 2.2 | 2m2m12 | 2.2.2 | 0 | 58 | 23 | 0 | 98 | 0 | 179 | 0.324 | 0.1285 | 0 | 0.5475 | 0 |
| 2 | June | 1 | ChildFeed | child | Bold | Bold | 1 | 3 | 2.3 | 2m3m1 | 2.1.3 | 0 | 21 | 81 | 0 | 10 | 107 | 219 | 0.0959 | 0.3699 | 0 | 0.0457 | 0.4886 |
| 2 | June | 1 | ChildFeed | child | Bold | Bold | 2 | 3 | 2.3 | 2m3m1 | 2.2.3 | 0 | 43 | 111 | 0 | 54 | 10 | 218 | 0.1972 | 0.5092 | 0 | 0.2477 | 0.0459 |
| 2 | June | 2 | ChildFeed | child | Bold | Bold | 1 | 3 | 2.3 | 2m3m2 | 2.1.3 | 0 | 88 | 63 | 0 | 26 | 18 | 195 | 0.4513 | 0.3231 | 0 | 0.1333 | 0.0923 |
| 2 | June | 2 | ChildFeed | child | Bold | Bold | 2 | 3 | 2.3 | 2m3m2 | 2.2.3 | 0 | 94 | 63 | 0 | 7 | 0 | 164 | 0.5732 | 0.3841 | 0 | 0.0427 | 0 |
| 2 | June | 3 | ChildFeed | child | Bold | Bold | 1 | 3 | 2.3 | 2m3m3 | 2.1.3 | 0 | 45 | 53 | 0 | 15 | 0 | 113 | 0.3982 | 0.469 | 0 | 0.1327 | 0 |
| 2 | June | 3 | ChildFeed | child | Bold | Bold | 2 | 3 | 2.3 | 2m3m3 | 2.2.3 | 0 | 44 | 46 | 9 | 11 | 21 | 131 | 0.3359 | 0.3511 | 0.0687 | 0.084 | 0.1603 |
| 2 | June | 4 | ChildFeed | child | Bold | Bold | 1 | 3 | 2.3 | 2m3m4 | 2.1.3 | 0 | 31 | 44 | 7 | 15 | 0 | 97 | 0.3196 | 0.4536 | 0.0722 | 0.1546 | 0 |
| 2 | June | 4 | ChildFeed | child | Bold | Bold | 2 | 3 | 2.3 | 2m3m4 | 2.2.3 | 0 | 0 | 12 | 0 | 19 | 29 | 60 | 0 | 0.2 | 0 | 0.3167 | 0.4833 |
| 2 | June | 5 | ChildFeed | child | Bold | Bold | 1 | 3 | 2.3 | 2m3m5 | 2.1.3 | 0 | 19 | 58 | 38 | 6 | 10 | 131 | 0.145 | 0.4427 | 0.2901 | 0.0458 | 0.0763 |
| 2 | June | 5 | ChildFeed | child | Bold | Bold | 2 | 3 | 2.3 | 2m3m5 | 2.2.3 | 0 | 14 | 34 | 25 | 6 | 53 | 132 | 0.1061 | 0.2576 | 0.1894 | 0.0455 | 0.4015 |
| 2 | June | 8 | ChildFeed | child | Bold | Bold | 1 | 3 | 2.3 | 2m3m8 | 2.1.3 | 0 | 102 | 65 | 8 | 53 | 14 | 242 | 0.4215 | 0.2686 | 0.0331 | 0.219 | 0.0579 |
| 2 | June | 8 | ChildFeed | child | Bold | Bold | 2 | 3 | 2.3 | 2m3m8 | 2.2.3 | 0 | 79 | 50 | 0 | 74 | 39 | 242 | 0.3264 | 0.2066 | 0 | 0.3058 | 0.1612 |
| 2 | June | 9 | ChildFeed | child | Bold | Bold | 1 | 3 | 2.3 | 2m3m9 | 2.1.3 | 0 | 42 | 132 | 12 | 56 | 12 | 254 | 0.1654 | 0.5197 | 0.0472 | 0.2205 | 0.0472 |
| 2 | June | 9 | ChildFeed | child | Bold | Bold | 2 | 3 | 2.3 | 2m3m9 | 2.2.3 | 0 | 57 | 92 | 18 | 64 | 23 | 254 | 0.2244 | 0.3622 | 0.0709 | 0.252 | 0.0906 |
| 2 | June | 10 | ChildFeed | child | Bold | Bold | 1 | 3 | 2.3 | 2m3m10 | 2.1.3 | 0 | 0 | 97 | 0 | 145 | 0 | 242 | 0 | 0.4008 | 0 | 0.5992 | 0 |
| 2 | June | 10 | ChildFeed | child | Bold | Bold | 2 | 3 | 2.3 | 2m3m10 | 2.2.3 | 0 | 25 | 39 | 0 | 86 | 92 | 242 | 0.1033 | 0.1612 | 0 | 0.3554 | 0.3802 |
| 2 | June | 11 | ChildFeed | child | Bold | Bold | 1 | 3 | 2.3 | 2m3m11 | 2.1.3 | 0 | 31 | 72 | 22 | 112 | 0 | 237 | 0.1308 | 0.3038 | 0.0928 | 0.4726 | 0 |
| 2 | June | 11 | ChildFeed | child | Bold | Bold | 2 | 3 | 2.3 | 2m3m11 | 2.2.3 | 0 | 19 | 64 | 0 | 154 | 0 | 237 | 0.0802 | 0.27 | 0 | 0.6498 | 0 |
| 2 | June | 12 | ChildFeed | child | Bold | Bold | 1 | 3 | 2.3 | 2m3m12 | 2.1.3 | 0 | 0 | 155 | 0 | 66 | 0 | 221 | 0 | 0.7014 | 0 | 0.2986 | 0 |
| 2 | June | 12 | ChildFeed | child | Bold | Bold | 2 | 3 | 2.3 | 2m3m12 | 2.2.3 | 0 | 8 | 72 | 0 | 13 | 128 | 221 | 0.0362 | 0.3258 | 0 | 0.0588 | 0.5792 |
| 2 | June | 1 | ChildWalk | child | Bold | Bold | 1 | 4 | 2.4 | 2m4m1 | 2.1.4 | 0 | 0 | 147 | 0 | 0 | 0 | 147 | 0 | 1 | 0 | 0 | 0 |
| 2 | June | 1 | ChildWalk | child | Bold | Bold | 2 | 4 | 2.4 | 2m4m1 | 2.2.4 | 0 | 11 | 109 | 0 | 0 | 27 | 147 | 0.0748 | 0.7415 | 0 | 0 | 0.1837 |
| 2 | June | 2 | ChildWalk | child | Bold | Bold | 1 | 4 | 2.4 | 2m4m2 | 2.1.4 | 0 | 48 | 148 | 0 | 46 | 10 | 252 | 0.1905 | 0.5873 | 0 | 0.1825 | 0.0397 |
| 2 | June | 2 | ChildWalk | child | Bold | Bold | 2 | 4 | 2.4 | 2m4m2 | 2.2.4 | 0 | 107 | 95 | 0 | 32 | 18 | 252 | 0.4246 | 0.377 | 0 | 0.127 | 0.0714 |
| 2 | June | 3 | ChildWalk | child | Bold | Bold | 1 | 4 | 2.4 | 2m4m3 | 2.1.4 | 0 | 100 | 98 | 0 | 17 | 0 | 215 | 0.4651 | 0.4558 | 0 | 0.0791 | 0 |
| 2 | June | 3 | ChildWalk | child | Bold | Bold | 2 | 4 | 2.4 | 2m4m3 | 2.2.4 | 0 | 73 | 79 | 15 | 48 | 0 | 215 | 0.3395 | 0.3674 | 0.0698 | 0.2233 | 0 |
| 2 | June | 4 | ChildWalk | child | Bold | Bold | 1 | 4 | 2.4 | 2m4m4 | 2.1.4 | 0 | 143 | 46 | 0 | 46 | 0 | 235 | 0.6085 | 0.1957 | 0 | 0.1957 | 0 |
| 2 | June | 4 | ChildWalk | child | Bold | Bold | 2 | 4 | 2.4 | 2m4m4 | 2.2.4 | 0 | 148 | 31 | 9 | 47 | 0 | 235 | 0.6298 | 0.1319 | 0.0383 | 0.2 | 0 |
| 2 | June | 5 | ChildWalk | child | Bold | Bold | 1 | 4 | 2.4 | 2m4m5 | 2.1.4 | 0 | 21 | 128 | 0 | 78 | 0 | 227 | 0.0925 | 0.5639 | 0 | 0.3436 | 0 |
| 2 | June | 5 | ChildWalk | child | Bold | Bold | 2 | 4 | 2.4 | 2m4m5 | 2.2.4 | 0 | 21 | 119 | 0 | 49 | 38 | 227 | 0.0925 | 0.5242 | 0 | 0.2159 | 0.1674 |
| 2 | June | 8 | ChildWalk | child | Bold | Bold | 1 | 4 | 2.4 | 2m4m8 | 2.1.4 | 0 | 39 | 45 | 0 | 149 | 0 | 233 | 0.1674 | 0.1931 | 0 | 0.6395 | 0 |
| 2 | June | 8 | ChildWalk | child | Bold | Bold | 2 | 4 | 2.4 | 2m4m8 | 2.2.4 | 0 | 73 | 55 | 0 | 67 | 38 | 233 | 0.3133 | 0.2361 | 0 | 0.2876 | 0.1631 |
| 2 | June | 9 | ChildWalk | child | Bold | Bold | 1 | 4 | 2.4 | 2m4m9 | 2.1.4 | 0 | 28 | 85 | 0 | 101 | 14 | 228 | 0.1228 | 0.3728 | 0 | 0.443 | 0.0614 |
| 2 | June | 9 | ChildWalk | child | Bold | Bold | 2 | 4 | 2.4 | 2m4m9 | 2.2.4 | 0 | 6 | 73 | 0 | 0 | 149 | 228 | 0.0263 | 0.3202 | 0 | 0 | 0.6535 |
| 2 | June | 10 | ChildWalk | child | Bold | Bold | 1 | 4 | 2.4 | 2m4m10 | 2.1.4 | 0 | 31 | 94 | 0 | 88 | 37 | 250 | 0.124 | 0.376 | 0 | 0.352 | 0.148 |
| 2 | June | 10 | ChildWalk | child | Bold | Bold | 2 | 4 | 2.4 | 2m4m10 | 2.2.4 | 0 | 8 | 97 | 0 | 65 | 80 | 250 | 0.032 | 0.388 | 0 | 0.26 | 0.32 |
| 2 | June | 11 | ChildWalk | child | Bold | Bold | 1 | 4 | 2.4 | 2m4m11 | 2.1.4 | 0 | 19 | 101 | 0 | 58 | 51 | 229 | 0.083 | 0.441 | 0 | 0.2533 | 0.2227 |
| 2 | June | 11 | ChildWalk | child | Bold | Bold | 2 | 4 | 2.4 | 2m4m11 | 2.2.4 | 0 | 48 | 60 | 0 | 31 | 90 | 229 | 0.2096 | 0.262 | 0 | 0.1354 | 0.393 |
| 2 | June | 12 | ChildWalk | child | Bold | Bold | 1 | 4 | 2.4 | 2m4m12 | 2.1.4 | 0 | 0 | 34 | 26 | 39 | 133 | 232 | 0 | 0.1466 | 0.1121 | 0.1681 | 0.5733 |
| 2 | June | 12 | ChildWalk | child | Bold | Bold | 2 | 4 | 2.4 | 2m4m12 | 2.2.4 | 0 | 0 | 101 | 36 | 81 | 14 | 232 | 0 | 0.4353 | 0.1552 | 0.3491 | 0.0603 |
| 2 | June | 1 | ChildWalk | child | Bold | Bold | 1 | 5 | 2.5 | 2m5m1 | 2.1.5 | 0 | 26 | 158 | 0 | 22 | 23 | 229 | 0.1135 | 0.69 | 0 | 0.0961 | 0.1004 |
| 2 | June | 1 | ChildWalk | child | Bold | Bold | 2 | 5 | 2.5 | 2m5m1 | 2.2.5 | 0 | 0 | 218 | 0 | 0 | 11 | 229 | 0 | 0.952 | 0 | 0 | 0.048 |
| 2 | June | 2 | ChildWalk | child | Bold | Bold | 1 | 5 | 2.5 | 2m5m2 | 2.1.5 | 0 | 0 | 201 | 0 | 40 | 0 | 241 | 0 | 0.834 | 0 | 0.166 | 0 |
| 2 | June | 2 | ChildWalk | child | Bold | Bold | 2 | 5 | 2.5 | 2m5m2 | 2.2.5 | 0 | 0 | 94 | 0 | 41 | 106 | 241 | 0 | 0.39 | 0 | 0.1701 | 0.4398 |
| 2 | June | 3 | ChildWalk | child | Bold | Bold | 1 | 5 | 2.5 | 2m5m3 | 2.1.5 | 0 | 0 | 105 | 0 | 0 | 107 | 212 | 0 | 0.4953 | 0 | 0 | 0.5047 |
| 2 | June | 3 | ChildWalk | child | Bold | Bold | 2 | 5 | 2.5 | 2m5m3 | 2.2.5 | 0 | 0 | 212 | 0 | 0 | 0 | 212 | 0 | 1 | 0 | 0 | 0 |
| 2 | June | 4 | ChildWalk | child | Bold | Bold | 1 | 5 | 2.5 | 2m5m4 | 2.1.5 | 0 | 0 | 120 | 6 | 0 | 94 | 220 | 0 | 0.5455 | 0.0273 | 0 | 0.4273 |
| 2 | June | 4 | ChildWalk | child | Bold | Bold | 2 | 5 | 2.5 | 2m5m4 | 2.2.5 | 0 | 0 | 195 | 3 | 0 | 22 | 220 | 0 | 0.8864 | 0.0136 | 0 | 0.1 |
| 2 | June | 5 | ChildWalk | child | Bold | Bold | 1 | 5 | 2.5 | 2m5m5 | 2.1.5 | 0 | 0 | 180 | 0 | 49 | 0 | 229 | 0 | 0.786 | 0 | 0.214 | 0 |
| 2 | June | 5 | ChildWalk | child | Bold | Bold | 2 | 5 | 2.5 | 2m5m5 | 2.2.5 | 0 | 0 | 192 | 0 | 37 | 0 | 229 | 0 | 0.8384 | 0 | 0.1616 | 0 |
| 2 | June | 11 | ChildWalk | child | Bold | Bold | 1 | 5 | 2.5 | 2m5m11 | 2.1.5 | 0 | 27 | 10 | 0 | 133 | 87 | 257 | 0.1051 | 0.0389 | 0 | 0.5175 | 0.3385 |
| 2 | June | 11 | ChildWalk | child | Bold | Bold | 2 | 5 | 2.5 | 2m5m11 | 2.2.5 | 0 | 0 | 33 | 0 | 224 | 0 | 257 | 0 | 0.1284 | 0 | 0.8716 | 0 |
| 2 | June | 1 | ChildWalk | child | Bold | Bold | 1 | 6 | 2.6 | 2m6m1 | 2.1.6 | 0 | 0 | 199 | 0 | 0 | 30 | 229 | 0 | 0.869 | 0 | 0 | 0.131 |
| 2 | June | 1 | ChildWalk | child | Bold | Bold | 2 | 6 | 2.6 | 2m6m1 | 2.2.6 | 0 | 17 | 47 | 0 | 43 | 122 | 229 | 0.0742 | 0.2052 | 0 | 0.1878 | 0.5328 |
| 2 | June | 2 | ChildWalk | child | Bold | Bold | 1 | 6 | 2.6 | 2m6m2 | 2.1.6 | 0 | 0 | 217 | 0 | 0 | 0 | 217 | 0 | 1 | 0 | 0 | 0 |
| 2 | June | 2 | ChildWalk | child | Bold | Bold | 2 | 6 | 2.6 | 2m6m2 | 2.2.6 | 0 | 73 | 0 | 0 | 55 | 89 | 217 | 0.3364 | 0 | 0 | 0.2535 | 0.4101 |

| | | | | | | | | | | | | | | | | | | | | | | | |
|---|---|---|---|---|---|---|---|---|---|---|---|---|---|---|---|---|---|---|---|---|---|---|---|
| 2 | June | 3 | ChildWalk | child | Bold | Bold | 1 | 6 | 2.6 | 2m6m3 | 2.1.6 | 0 | 0 | 203 | 0 | 8 | 0 | 211 | 0 | 0.9621 | 0 | 0.0379 | 0 |
| 2 | June | 3 | ChildWalk | child | Bold | Bold | 2 | 6 | 2.6 | 2m6m3 | 2.2.6 | 0 | 36 | 0 | 0 | 0 | 175 | 211 | 0.1706 | 0 | 0 | 0 | 0.8294 |
| 2 | June | 4 | ChildWalk | child | Bold | Bold | 1 | 6 | 2.6 | 2m6m4 | 2.1.6 | 0 | 0 | 183 | 6 | 0 | 63 | 252 | 0 | 0.7262 | 0.0238 | 0 | 0.25 |
| 2 | June | 4 | ChildWalk | child | Bold | Bold | 2 | 6 | 2.6 | 2m6m4 | 2.2.6 | 0 | 19 | 51 | 0 | 7 | 175 | 252 | 0.0754 | 0.2024 | 0 | 0.0278 | 0.6944 |
| 2 | June | 5 | ChildWalk | child | Bold | Bold | 1 | 6 | 2.6 | 2m6m5 | 2.1.6 | 0 | 11 | 215 | 0 | 10 | 0 | 236 | 0.0466 | 0.911 | 0 | 0.0424 | 0 |
| 2 | June | 5 | ChildWalk | child | Bold | Bold | 2 | 6 | 2.6 | 2m6m5 | 2.2.6 | 0 | 53 | 0 | 0 | 72 | 111 | 236 | 0.2246 | 0 | 0 | 0.3051 | 0.4703 |
| 3 | July | 1 | ChildFeed | child | Bold | Bold | 1 | 1 | 3.1 | 3m1m1 | 3.1.1 | 0 | 9 | 109 | 11 | 26 | 62 | 217 | 0.0415 | 0.5023 | 0.0507 | 0.1198 | 0.2857 |
| 3 | July | 1 | ChildFeed | child | Bold | Bold | 2 | 1 | 3.1 | 3m1m1 | 3.2.1 | 0 | 49 | 87 | 3 | 2 | 74 | 215 | 0.2279 | 0.4047 | 0.014 | 0.0093 | 0.3442 |
| 3 | July | 2 | ChildFeed | child | Bold | Bold | 1 | 1 | 3.1 | 3m1m2 | 3.1.1 | 0 | 18 | 132 | 5 | 0 | 44 | 199 | 0.0905 | 0.6633 | 0.0251 | 0 | 0.2211 |
| 3 | July | 2 | ChildFeed | child | Bold | Bold | 2 | 1 | 3.1 | 3m1m2 | 3.2.1 | 0 | 16 | 70 | 2 | 10 | 85 | 183 | 0.0874 | 0.3825 | 0.0109 | 0.0546 | 0.4645 |
| 3 | July | 3 | ChildFeed | child | Bold | Bold | 1 | 1 | 3.1 | 3m1m3 | 3.1.1 | 0 | 0 | 180 | 3 | 0 | 22 | 205 | 0 | 0.878 | 0.0146 | 0 | 0.1073 |
| 3 | July | 3 | ChildFeed | child | Bold | Bold | 2 | 1 | 3.1 | 3m1m3 | 3.2.1 | 0 | 11 | 176 | 0 | 9 | 10 | 206 | 0.0534 | 0.8544 | 0 | 0.0437 | 0.0485 |
| 3 | July | 4 | ChildFeed | child | Bold | Bold | 1 | 1 | 3.1 | 3m1m4 | 3.1.1 | 0 | 51 | 107 | 10 | 7 | 58 | 233 | 0.2189 | 0.4592 | 0.0429 | 0.03 | 0.2489 |
| 3 | July | 4 | ChildFeed | child | Bold | Bold | 2 | 1 | 3.1 | 3m1m4 | 3.2.1 | 0 | 73 | 105 | 15 | 17 | 0 | 210 | 0.3476 | 0.5 | 0.0714 | 0.081 | 0 |
| 3 | July | 5 | ChildFeed | child | Bold | Bold | 1 | 1 | 3.1 | 3m1m5 | 3.1.1 | 0 | 47 | 224 | 4 | 0 | 0 | 275 | 0.1709 | 0.8145 | 0.0145 | 0 | 0 |
| 3 | July | 5 | ChildFeed | child | Bold | Bold | 2 | 1 | 3.1 | 3m1m5 | 3.2.1 | 0 | 17 | 234 | 4 | 0 | 20 | 275 | 0.0618 | 0.8509 | 0.0145 | 0 | 0.0727 |
| 3 | July | 1 | ChildFeed | child | Bold | Bold | 1 | 2 | 3.2 | 3m2m1 | 3.1.2 | 0 | 67 | 80 | 0 | 37 | 44 | 228 | 0.2939 | 0.3509 | 0 | 0.1623 | 0.193 |
| 3 | July | 1 | ChildFeed | child | Bold | Bold | 2 | 2 | 3.2 | 3m2m1 | 3.2.2 | 0 | 74 | 50 | 0 | 25 | 79 | 228 | 0.3246 | 0.2193 | 0 | 0.1096 | 0.3465 |
| 3 | July | 2 | ChildFeed | child | Bold | Bold | 1 | 2 | 3.2 | 3m2m2 | 3.1.2 | 0 | 109 | 128 | 3 | 21 | 36 | 297 | 0.367 | 0.431 | 0.0101 | 0.0707 | 0.1212 |
| 3 | July | 2 | ChildFeed | child | Bold | Bold | 2 | 2 | 3.2 | 3m2m2 | 3.2.2 | 0 | 90 | 130 | 0 | 22 | 55 | 297 | 0.303 | 0.4377 | 0 | 0.0741 | 0.1852 |
| 3 | July | 3 | ChildFeed | child | Bold | Bold | 1 | 2 | 3.2 | 3m2m3 | 3.1.2 | 0 | 51 | 102 | 15 | 28 | 108 | 304 | 0.1678 | 0.3355 | 0.0493 | 0.0921 | 0.3553 |
| 3 | July | 3 | ChildFeed | child | Bold | Bold | 2 | 2 | 3.2 | 3m2m3 | 3.2.2 | 0 | 59 | 160 | 10 | 51 | 24 | 304 | 0.1941 | 0.5263 | 0.0329 | 0.1678 | 0.0789 |
| 3 | July | 4 | ChildFeed | child | Bold | Bold | 1 | 2 | 3.2 | 3m2m4 | 3.1.2 | 0 | 100 | 117 | 8 | 54 | 29 | 308 | 0.3247 | 0.3799 | 0.026 | 0.1753 | 0.0942 |
| 3 | July | 4 | ChildFeed | child | Bold | Bold | 2 | 2 | 3.2 | 3m2m4 | 3.2.2 | 0 | 132 | 98 | 0 | 59 | 19 | 308 | 0.4286 | 0.3182 | 0 | 0.1916 | 0.0617 |
| 3 | July | 5 | ChildFeed | child | Bold | Bold | 1 | 2 | 3.2 | 3m2m5 | 3.1.2 | 0 | 69 | 159 | 4 | 14 | 0 | 246 | 0.2805 | 0.6463 | 0.0163 | 0.0569 | 0 |
| 3 | July | 5 | ChildFeed | child | Bold | Bold | 2 | 2 | 3.2 | 3m2m5 | 3.2.2 | 0 | 127 | 111 | 0 | 22 | 0 | 260 | 0.4885 | 0.4269 | 0 | 0.0846 | 0 |
| 3 | July | 8 | ChildFeed | child | Bold | Bold | 1 | 2 | 3.2 | 3m2m8 | 3.1.2 | 0 | 116 | 51 | 0 | 63 | 20 | 250 | 0.464 | 0.204 | 0 | 0.252 | 0.08 |
| 3 | July | 8 | ChildFeed | child | Bold | Bold | 2 | 2 | 3.2 | 3m2m8 | 3.2.2 | 0 | 136 | 23 | 0 | 48 | 43 | 250 | 0.544 | 0.092 | 0 | 0.192 | 0.172 |
| 3 | July | 10 | ChildFeed | child | Bold | Bold | 1 | 2 | 3.2 | 3m2m10 | 3.1.2 | 0 | 114 | 117 | 4 | 46 | 4 | 285 | 0.4 | 0.4105 | 0.014 | 0.1614 | 0.014 |
| 3 | July | 10 | ChildFeed | child | Bold | Bold | 2 | 2 | 3.2 | 3m2m10 | 3.2.2 | 0 | 58 | 101 | 20 | 106 | 0 | 285 | 0.2035 | 0.3544 | 0.0702 | 0.3719 | 0 |
| 3 | July | 11 | ChildFeed | child | Bold | Bold | 1 | 2 | 3.2 | 3m2m11 | 3.1.2 | 0 | 54 | 153 | 0 | 64 | 0 | 271 | 0.1993 | 0.5646 | 0 | 0.2362 | 0 |
| 3 | July | 11 | ChildFeed | child | Bold | Bold | 2 | 2 | 3.2 | 3m2m11 | 3.2.2 | 0 | 108 | 98 | 8 | 57 | 0 | 271 | 0.3985 | 0.3616 | 0.0295 | 0.2103 | 0 |
| 3 | July | 12 | ChildFeed | child | Bold | Bold | 1 | 2 | 3.2 | 3m2m12 | 3.1.2 | 0 | 104 | 113 | 0 | 34 | 25 | 276 | 0.3768 | 0.4094 | 0 | 0.1232 | 0.0906 |
| 3 | July | 12 | ChildFeed | child | Bold | Bold | 2 | 2 | 3.2 | 3m2m12 | 3.2.2 | 0 | 81 | 119 | 0 | 56 | 20 | 276 | 0.2935 | 0.4312 | 0 | 0.2029 | 0.0725 |
| 3 | July | 1 | ChildFeed | child | Bold | Bold | 1 | 3 | 3.3 | 3m3m1 | 3.1.3 | 0 | 15 | 130 | 2 | 15 | 49 | 211 | 0.0711 | 0.6161 | 0.0095 | 0.0711 | 0.2322 |
| 3 | July | 1 | ChildFeed | child | Bold | Bold | 2 | 3 | 3.3 | 3m3m1 | 3.2.3 | 0 | 2 | 131 | 2 | 13 | 80 | 228 | 0.0088 | 0.5746 | 0.0088 | 0.057 | 0.3509 |
| 3 | July | 2 | ChildFeed | child | Bold | Bold | 1 | 3 | 3.3 | 3m3m2 | 3.1.3 | 0 | 11 | 145 | 0 | 10 | 49 | 215 | 0.0512 | 0.6744 | 0 | 0.0465 | 0.2279 |
| 3 | July | 2 | ChildFeed | child | Bold | Bold | 2 | 3 | 3.3 | 3m3m2 | 3.2.3 | 0 | 28 | 88 | 0 | 3 | 49 | 168 | 0.1667 | 0.5238 | 0 | 0.0179 | 0.2917 |
| 3 | July | 3 | ChildFeed | child | Bold | Bold | 1 | 3 | 3.3 | 3m3m3 | 3.1.3 | 0 | 24 | 76 | 0 | 5 | 0 | 105 | 0.2286 | 0.7238 | 0 | 0.0476 | 0 |
| 3 | July | 3 | ChildFeed | child | Bold | Bold | 2 | 3 | 3.3 | 3m3m3 | 3.2.3 | 0 | 27 | 54 | 2 | 0 | 32 | 115 | 0.2348 | 0.4696 | 0.0174 | 0 | 0.2783 |
| 3 | July | 4 | ChildFeed | child | Bold | Bold | 1 | 3 | 3.3 | 3m3m4 | 3.1.3 | 0 | 0 | 80 | 4 | 0 | 11 | 95 | 0 | 0.8421 | 0.0421 | 0 | 0.1158 |
| 3 | July | 4 | ChildFeed | child | Bold | Bold | 2 | 3 | 3.3 | 3m3m4 | 3.2.3 | 0 | 29 | 102 | 0 | 16 | 27 | 174 | 0.1667 | 0.5862 | 0 | 0.092 | 0.1552 |
| 3 | July | 5 | ChildFeed | child | Bold | Bold | 1 | 3 | 3.3 | 3m3m5 | 3.1.3 | 0 | 15 | 110 | 13 | 32 | 18 | 188 | 0.0798 | 0.5851 | 0.0691 | 0.1702 | 0.0957 |
| 3 | July | 5 | ChildFeed | child | Bold | Bold | 2 | 3 | 3.3 | 3m3m5 | 3.2.3 | 0 | 18 | 150 | 18 | 32 | 0 | 218 | 0.0826 | 0.6881 | 0.0826 | 0.1468 | 0 |
| 3 | July | 1 | ChildWalk | child | Bold | Bold | 1 | 4 | 3.4 | 3m4m1 | 3.1.4 | 0 | 63 | 84 | 0 | 34 | 44 | 225 | 0.28 | 0.3733 | 0 | 0.1511 | 0.1956 |
| 3 | July | 1 | ChildWalk | child | Bold | Bold | 2 | 4 | 3.4 | 3m4m1 | 3.2.4 | 0 | 0 | 221 | 0 | 0 | 4 | 225 | 0 | 0.9822 | 0 | 0 | 0.0178 |
| 3 | July | 2 | ChildWalk | child | Bold | Bold | 1 | 4 | 3.4 | 3m4m2 | 3.1.4 | 0 | 6 | 107 | 11 | 24 | 105 | 253 | 0.0237 | 0.4229 | 0.0435 | 0.0949 | 0.415 |
| 3 | July | 2 | ChildWalk | child | Bold | Bold | 2 | 4 | 3.4 | 3m4m2 | 3.2.4 | 0 | 5 | 182 | 2 | 14 | 50 | 253 | 0.0198 | 0.7194 | 0.0079 | 0.0553 | 0.1976 |
| 3 | July | 3 | ChildWalk | child | Bold | Bold | 1 | 4 | 3.4 | 3m4m3 | 3.1.4 | 0 | 6 | 214 | 0 | 24 | 15 | 259 | 0.0232 | 0.8263 | 0 | 0.0927 | 0.0579 |
| 3 | July | 3 | ChildWalk | child | Bold | Bold | 2 | 4 | 3.4 | 3m4m3 | 3.2.4 | 0 | 0 | 245 | 3 | 0 | 11 | 259 | 0 | 0.9459 | 0.0116 | 0 | 0.0425 |
| 3 | July | 4 | ChildWalk | child | Bold | Bold | 1 | 4 | 3.4 | 3m4m4 | 3.1.4 | 0 | 0 | 300 | 0 | 0 | 0 | 300 | 0 | 1 | 0 | 0 | 0 |
| 3 | July | 4 | ChildWalk | child | Bold | Bold | 2 | 4 | 3.4 | 3m4m4 | 3.2.4 | 0 | 0 | 300 | 0 | 0 | 0 | 300 | 0 | 1 | 0 | 0 | 0 |
| 3 | July | 5 | ChildWalk | child | Bold | Bold | 1 | 4 | 3.4 | 3m4m5 | 3.1.4 | 0 | 9 | 223 | 49 | 8 | 0 | 289 | 0.0311 | 0.7716 | 0.1696 | 0.0277 | 0 |
| 3 | July | 5 | ChildWalk | child | Bold | Bold | 2 | 4 | 3.4 | 3m4m5 | 3.2.4 | 0 | 14 | 222 | 33 | 20 | 0 | 289 | 0.0484 | 0.7682 | 0.1142 | 0.0692 | 0 |
| 3 | July | 12 | ChildWalk | child | Bold | Bold | 1 | 4 | 3.4 | 3m4m12 | 3.1.4 | 0 | 0 | 7 | 0 | 309 | 0 | 316 | 0 | 0.0222 | 0 | 0.9778 | 0 |
| 3 | July | 12 | ChildWalk | child | Bold | Bold | 2 | 4 | 3.4 | 3m4m12 | 3.2.4 | 0 | 0 | 0 | 0 | 316 | 0 | 316 | 0 | 0 | 0 | 1 | 0 |
| 3 | July | 1 | ChildWalk | child | boldshy | Bold | 1 | 5 | 3.5 | 3m5m1 | 3.1.5 | 0 | 0 | 214 | 0 | 11 | 27 | 252 | 0 | 0.8492 | 0 | 0.0437 | 0.1071 |
| 3 | July | 1 | ChildWalk | child | boldshy | Bold | 2 | 5 | 3.5 | 3m5m1 | 3.2.5 | 0 | 0 | 77 | 0 | 5 | 170 | 252 | 0 | 0.3056 | 0 | 0.0198 | 0.6746 |
| 3 | July | 2 | ChildWalk | child | boldshy | Bold | 1 | 5 | 3.5 | 3m5m2 | 3.1.5 | 0 | 0 | 39 | 0 | 39 | 185 | 263 | 0 | 0.1483 | 0 | 0.1483 | 0.7034 |
| 3 | July | 2 | ChildWalk | child | boldshy | Bold | 2 | 5 | 3.5 | 3m5m2 | 3.2.5 | 0 | 5 | 114 | 21 | 43 | 80 | 263 | 0.019 | 0.4335 | 0.0798 | 0.1635 | 0.3042 |
| 3 | July | 3 | ChildWalk | child | boldshy | Bold | 1 | 5 | 3.5 | 3m5m3 | 3.1.5 | 0 | 0 | 173 | 43 | 28 | 24 | 268 | 0 | 0.6455 | 0.1604 | 0.1045 | 0.0896 |
| 3 | July | 3 | ChildWalk | child | boldshy | Bold | 2 | 5 | 3.5 | 3m5m3 | 3.2.5 | 0 | 6 | 90 | 0 | 56 | 116 | 268 | 0.0224 | 0.3358 | 0 | 0.209 | 0.4328 |
| 3 | July | 4 | ChildWalk | child | boldshy | Bold | 1 | 5 | 3.5 | 3m5m4 | 3.1.5 | 0 | 0 | 75 | 15 | 65 | 109 | 264 | 0 | 0.2841 | 0.0568 | 0.2462 | 0.4129 |
| 3 | July | 4 | ChildWalk | child | boldshy | Bold | 2 | 5 | 3.5 | 3m5m4 | 3.2.5 | 0 | 0 | 28 | 5 | 164 | 67 | 264 | 0 | 0.1061 | 0.0189 | 0.6212 | 0.2538 |
| 3 | July | 5 | ChildWalk | child | boldshy | Bold | 1 | 5 | 3.5 | 3m5m5 | 3.1.5 | 0 | 36 | 8 | 35 | 227 | 0 | 306 | 0.1176 | 0.0261 | 0.1144 | 0.7418 | 0 |
| 3 | July | 5 | ChildWalk | child | boldshy | Bold | 2 | 5 | 3.5 | 3m5m5 | 3.2.5 | 0 | 0 | 0 | 0 | 306 | 0 | 306 | 0 | 0 | 0 | 1 | 0 |
| 3 | July | 9 | ChildWalk | child | boldshy | Bold | 1 | 5 | 3.5 | 3m5m9 | 3.1.5 | 0 | 0 | 0 | 0 | 307 | 0 | 307 | 0 | 0 | 0 | 1 | 0 |
| 3 | July | 9 | ChildWalk | child | boldshy | Bold | 2 | 5 | 3.5 | 3m5m9 | 3.2.5 | 0 | 16 | 25 | 0 | 266 | 0 | 307 | 0.0521 | 0.0814 | 0 | 0.8664 | 0 |
| 3 | July | 10 | ChildWalk | child | boldshy | Bold | 1 | 5 | 3.5 | 3m5m10 | 3.1.5 | 0 | 111 | 21 | 24 | 124 | 13 | 293 | 0.3788 | 0.0717 | 0.0819 | 0.4232 | 0.0444 |
| 3 | July | 10 | ChildWalk | child | boldshy | Bold | 2 | 5 | 3.5 | 3m5m10 | 3.2.5 | 0 | 105 | 17 | 11 | 151 | 10 | 294 | 0.3571 | 0.0578 | 0.0374 | 0.5136 | 0.034 |
| 3 | July | 11 | ChildWalk | child | boldshy | Bold | 1 | 5 | 3.5 | 3m5m11 | 3.1.5 | 0 | 0 | 0 | 0 | 308 | 0 | 308 | 0 | 0 | 0 | 1 | 0 |
| 3 | July | 11 | ChildWalk | child | boldshy | Bold | 2 | 5 | 3.5 | 3m5m11 | 3.2.5 | 0 | 0 | 0 | 0 | 308 | 0 | 308 | 0 | 0 | 0 | 1 | 0 |
| 3 | July | 12 | ChildWalk | child | boldshy | Bold | 1 | 5 | 3.5 | 3m5m12 | 3.1.5 | 0 | 10 | 10 | 0 | 299 | 0 | 319 | 0.0313 | 0.0313 | 0 | 0.9373 | 0 |
| 3 | July | 12 | ChildWalk | child | boldshy | Bold | 2 | 5 | 3.5 | 3m5m12 | 3.2.5 | 0 | 0 | 0 | 0 | 319 | 0 | 319 | 0 | 0 | 0 | 1 | 0 |
| 3 | July | 1 | ChildWalk | child | boldshy | Bold | 1 | 7 | 3.7 | 3m7m1 | 3.1.7 | 0 | 16 | 152 | 0 | 53 | 22 | 243 | 0.0658 | 0.6255 | 0 | 0.2181 | 0.0905 |
| 3 | July | 1 | ChildWalk | child | boldshy | Bold | 2 | 7 | 3.7 | 3m7m1 | 3.2.7 | 0 | 22 | 111 | 20 | 63 | 27 | 243 | 0.0905 | 0.4568 | 0.0823 | 0.2593 | 0.1111 |
| 3 | July | 2 | ChildWalk | child | boldshy | Bold | 1 | 7 | 3.7 | 3m7m2 | 3.1.7 | 0 | 0 | 66 | 0 | 37 | 22 | 125 | 0 | 0.528 | 0 | 0.296 | 0.176 |
| 3 | July | 2 | ChildWalk | child | boldshy | Bold | 2 | 7 | 3.7 | 3m7m2 | 3.2.7 | 0 | 7 | 11 | 0 | 8 | 99 | 125 | 0.056 | 0.088 | 0 | 0.064 | 0.792 |
| 3 | July | 3 | ChildWalk | child | boldshy | Bold | 1 | 7 | 3.7 | 3m7m3 | 3.1.7 | 0 | 42 | 54 | 11 | 61 | 100 | 268 | 0.1567 | 0.2015 | 0.041 | 0.2276 | 0.3731 |
| 3 | July | 3 | ChildWalk | child | boldshy | Bold | 2 | 7 | 3.7 | 3m7m3 | 3.2.7 | 0 | 14 | 20 | 0 | 81 | 153 | 268 | 0.0522 | 0.0746 | 0 | 0.3022 | 0.5709 |
| 3 | July | 4 | ChildWalk | child | boldshy | Bold | 1 | 7 | 3.7 | 3m7m4 | 3.1.7 | 0 | 9 | 47 | 0 | 130 | 92 | 278 | 0.0324 | 0.1691 | 0 | 0.4676 | 0.3309 |
| 3 | July | 4 | ChildWalk | child | boldshy | Bold | 2 | 7 | 3.7 | 3m7m4 | 3.2.7 | 0 | 17 | 122 | 6 | 133 | 0 | 278 | 0.0612 | 0.4388 | 0.0216 | 0.4784 | 0 |
| 3 | July | 5 | ChildWalk | child | boldshy | Bold | 1 | 7 | 3.7 | 3m7m5 | 3.1.7 | 0 | 82 | 142 | 22 | 50 | 0 | 296 | 0.277 | 0.4797 | 0.0743 | 0.1689 | 0 |
| 3 | July | 5 | ChildWalk | child | boldshy | Bold | 2 | 7 | 3.7 | 3m7m5 | 3.2.7 | 0 | 43 | 137 | 17 | 54 | 45 | 296 | 0.1453 | 0.4628 | 0.0574 | 0.1824 | 0.152 |
| 3 | July | 12 | aChildaControl | child | boldshy | Bold | 1 | 7 | 3.7 | 3m7m12 | 3.1.7 | 0 | 18 | 87 | 0 | 96 | 133 | 334 | 0.0539 | 0.2605 | 0 | 0.2874 | 0.3982 |
| 3 | July | 12 | aChildaControl | child | boldshy | Bold | 2 | 7 | 3.7 | 3m7m12 | 3.2.7 | 0 | 29 | 0 | 0 | 180 | 125 | 334 | 0.0868 | 0 | 0 | 0.5389 | 0.3743 |
| 3 | July | 10 | ChildWalk | child | Bold | Bold | 1 | 8 | 3.8 | 3m8m10 | 3.1.8 | 0 | 29 | 19 | 139 | 28 | 107 | 322 | 0.0901 | 0.059 | 0.4317 | 0.087 | 0.3323 |
| 3 | July | 10 | ChildWalk | child | Bold | Bold | 2 | 8 | 3.8 | 3m8m10 | 3.2.8 | 0 | 35 | 197 | 11 | 51 | 28 | 322 | 0.1087 | 0.6118 | 0.0342 | 0.1584 | 0.087 |
| 3 | July | 12 | ChildWalk | child | Bold | Bold | 1 | 8 | 3.8 | 3m8m12 | 3.1.8 | 0 | 3 | 145 | 13 | 160 | 10 | 331 | 0.0091 | 0.4381 | 0.0393 | 0.4834 | 0.0302 |
| 3 | July | 12 | ChildWalk | child | Bold | Bold | 2 | 8 | 3.8 | 3m8m12 | 3.2.8 | 0 | 0 | 0 | 0 | 331 | 0 | 331 | 0 | 0 | 0 | 1 | 0 |
| 4 | August | 1 | Dog | adult | Bold | Bold | 1 | 1 | 4.1 | 4m1m1 | 4.1.1 | 0 | 30 | 164 | 0 | 33 | 7 | 234 | 0.1282 | 0.7009 | 0 | 0.141 | 0.0299 |
| 4 | August | 1 | Dog | adult | Bold | Bold | 2 | 1 | 4.1 | 4m1m1 | 4.2.1 | 0 | 6 | 0 | 0 | 0 | 228 | 234 | 0.0256 | 0 | 0 | 0 | 0.9744 |
| 4 | August | 2 | Dog | adult | Bold | Bold | 1 | 1 | 4.1 | 4m1m2 | 4.1.1 | 0 | 35 | 114 | 0 | 16 | 26 | 191 | 0.1832 | 0.5969 | 0 | 0.0838 | 0.1361 |

| | | | | | | | | | | | | | | | | | | | | | | | |
|---|---|---|---|---|---|---|---|---|---|---|---|---|---|---|---|---|---|---|---|---|---|---|---|
| 4 | August | 2 | Dog | adult | Bold | Bold | 2 | 1 | 4.1 | 4m1m2 | 4.2.1 | 0 | 20 | 11 | 0 | 4 | 156 | 191 | 0.1047 | 0.0576 | 0 | 0.0209 | 0.8168 |
| 4 | August | 3 | Dog | adult | Bold | Bold | 1 | 1 | 4.1 | 4m1m3 | 4.1.1 | 0 | 45 | 141 | 24 | 33 | 11 | 254 | 0.1772 | 0.5551 | 0.0945 | 0.1299 | 0.0433 |
| 4 | August | 3 | Dog | adult | Bold | Bold | 2 | 1 | 4.1 | 4m1m3 | 4.2.1 | 0 | 62 | 21 | 6 | 13 | 152 | 254 | 0.2441 | 0.0827 | 0.0236 | 0.0512 | 0.5984 |
| 4 | August | 4 | Dog | adult | Bold | Bold | 1 | 1 | 4.1 | 4m1m4 | 4.1.1 | 0 | 32 | 47 | 0 | 29 | 129 | 237 | 0.135 | 0.1983 | 0 | 0.1224 | 0.5443 |
| 4 | August | 4 | Dog | adult | Bold | Bold | 2 | 1 | 4.1 | 4m1m4 | 4.2.1 | 0 | 13 | 73 | 13 | 6 | 132 | 237 | 0.0549 | 0.308 | 0.0549 | 0.0253 | 0.557 |
| 4 | August | 5 | Dog | adult | Bold | Bold | 1 | 1 | 4.1 | 4m1m5 | 4.1.1 | 0 | 111 | 74 | 23 | 5 | 32 | 245 | 0.4531 | 0.302 | 0.0939 | 0.0204 | 0.1306 |
| 4 | August | 5 | Dog | adult | Bold | Bold | 2 | 1 | 4.1 | 4m1m5 | 4.2.1 | 0 | 160 | 27 | 0 | 26 | 32 | 245 | 0.6531 | 0.1102 | 0 | 0.1061 | 0.1306 |
| 4 | August | 8 | Dog | adult | Bold | Bold | 2 | 1 | 4.1 | 4m1m8 | 4.2.1 | 0 | 15 | 18 | 0 | 0 | 198 | 231 | 0.0649 | 0.0779 | 0 | 0 | 0.8571 |
| 4 | August | 9 | Dog | adult | Bold | Bold | 2 | 1 | 4.1 | 4m1m9 | 4.2.1 | 0 | 55 | 50 | 5 | 101 | 30 | 241 | 0.2282 | 0.2075 | 0.0207 | 0.4191 | 0.1245 |
| 4 | August | 10 | Dog | adult | Bold | Bold | 2 | 1 | 4.1 | 4m1m10 | 4.2.1 | 0 | 61 | 0 | 13 | 5 | 123 | 202 | 0.302 | 0 | 0.0644 | 0.0248 | 0.6089 |
| 4 | August | 11 | Dog | adult | Bold | Bold | 2 | 1 | 4.1 | 4m1m11 | 4.2.1 | 0 | 7 | 21 | 16 | 0 | 179 | 223 | 0.0314 | 0.0942 | 0.0717 | 0 | 0.8027 |
| 4 | August | 12 | Dog | adult | Bold | Bold | 2 | 1 | 4.1 | 4m1m12 | 4.2.1 | 0 | 27 | 86 | 10 | 19 | 100 | 242 | 0.1116 | 0.3554 | 0.0413 | 0.0785 | 0.4132 |
| 4 | August | 1 | Dog | adult | Bold | Bold | 1 | 2 | 4.2 | 4m2m1 | 4.1.2 | 0 | 85 | 20 | 0 | 35 | 75 | 215 | 0.3953 | 0.093 | 0 | 0.1628 | 0.3488 |
| 4 | August | 1 | Dog | adult | Bold | Bold | 2 | 2 | 4.2 | 4m2m1 | 4.2.2 | 0 | 33 | 9 | 0 | 46 | 127 | 215 | 0.1535 | 0.0419 | 0 | 0.214 | 0.5907 |
| 4 | August | 2 | Dog | adult | Bold | Bold | 1 | 2 | 4.2 | 4m2m2 | 4.1.2 | 0 | 56 | 34 | 0 | 54 | 87 | 231 | 0.2424 | 0.1472 | 0 | 0.2338 | 0.3766 |
| 4 | August | 2 | Dog | adult | Bold | Bold | 2 | 2 | 4.2 | 4m2m2 | 4.2.2 | 0 | 162 | 0 | 0 | 5 | 64 | 231 | 0.7013 | 0 | 0 | 0.0216 | 0.2771 |
| 4 | August | 3 | Dog | adult | Bold | Bold | 1 | 2 | 4.2 | 4m2m3 | 4.1.2 | 0 | 91 | 34 | 0 | 40 | 81 | 246 | 0.3699 | 0.1382 | 0 | 0.1626 | 0.3293 |
| 4 | August | 3 | Dog | adult | Bold | Bold | 2 | 2 | 4.2 | 4m2m3 | 4.2.2 | 0 | 120 | 5 | 0 | 11 | 110 | 246 | 0.4878 | 0.0203 | 0 | 0.0447 | 0.4472 |
| 4 | August | 4 | Dog | adult | Bold | Bold | 1 | 2 | 4.2 | 4m2m4 | 4.1.2 | 0 | 11 | 23 | 0 | 15 | 174 | 223 | 0.0493 | 0.1031 | 0 | 0.0673 | 0.7803 |
| 4 | August | 4 | Dog | adult | Bold | Bold | 2 | 2 | 4.2 | 4m2m4 | 4.2.2 | 0 | 6 | 6 | 0 | 0 | 211 | 223 | 0.0269 | 0.0269 | 0 | 0 | 0.9462 |
| 4 | August | 5 | Dog | adult | Bold | Bold | 1 | 2 | 4.2 | 4m2m5 | 4.1.2 | 0 | 50 | 0 | 0 | 0 | 176 | 226 | 0.2212 | 0 | 0 | 0 | 0.7788 |
| 4 | August | 5 | Dog | adult | Bold | Bold | 2 | 2 | 4.2 | 4m2m5 | 4.2.2 | 0 | 116 | 0 | 0 | 0 | 110 | 226 | 0.5133 | 0 | 0 | 0 | 0.4867 |
| 4 | August | 8 | Dog | adult | Bold | Bold | 1 | 2 | 4.2 | 4m2m8 | 4.1.2 | 0 | 27 | 8 | 0 | 6 | 168 | 209 | 0.1292 | 0.0383 | 0 | 0.0287 | 0.8038 |
| 4 | August | 8 | Dog | adult | Bold | Bold | 2 | 2 | 4.2 | 4m2m8 | 4.2.2 | 0 | 40 | 9 | 0 | 14 | 146 | 209 | 0.1914 | 0.0431 | 0 | 0.067 | 0.6986 |
| 4 | August | 9 | Dog | adult | Bold | Bold | 1 | 2 | 4.2 | 4m2m9 | 4.1.2 | 0 | 110 | 0 | 0 | 0 | 112 | 222 | 0.4955 | 0 | 0 | 0 | 0.5045 |
| 4 | August | 9 | Dog | adult | Bold | Bold | 2 | 2 | 4.2 | 4m2m9 | 4.2.2 | 0 | 105 | 0 | 0 | 0 | 117 | 222 | 0.473 | 0 | 0 | 0 | 0.527 |
| 4 | August | 10 | Dog | adult | Bold | Bold | 1 | 2 | 4.2 | 4m2m10 | 4.1.2 | 0 | 30 | 24 | 0 | 21 | 145 | 220 | 0.1364 | 0.1091 | 0 | 0.0955 | 0.6591 |
| 4 | August | 10 | Dog | adult | Bold | Bold | 2 | 2 | 4.2 | 4m2m10 | 4.2.2 | 0 | 14 | 0 | 0 | 0 | 206 | 220 | 0.0636 | 0 | 0 | 0 | 0.9364 |
| 4 | August | 11 | Dog | adult | Bold | Bold | 1 | 2 | 4.2 | 4m2m11 | 4.1.2 | 0 | 83 | 3 | 9 | 0 | 128 | 223 | 0.3722 | 0.0135 | 0.0404 | 0 | 0.574 |
| 4 | August | 11 | Dog | adult | Bold | Bold | 2 | 2 | 4.2 | 4m2m11 | 4.2.2 | 0 | 42 | 0 | 0 | 5 | 176 | 223 | 0.1883 | 0 | 0 | 0.0224 | 0.7892 |
| 4 | August | 1 | Feed | adult | Bold | Bold | 1 | 3 | 4.3 | 4m3m1 | 4.1.3 | 0 | 9 | 160 | 2 | 0 | 0 | 171 | 0.0526 | 0.9357 | 0.0117 | 0 | 0 |
| 4 | August | 1 | Feed | adult | Bold | Bold | 2 | 3 | 4.3 | 4m3m1 | 4.2.3 | 0 | 44 | 103 | 0 | 0 | 31 | 178 | 0.2472 | 0.5787 | 0 | 0 | 0.1742 |
| 4 | August | 2 | Feed | adult | Bold | Bold | 1 | 3 | 4.3 | 4m3m2 | 4.1.3 | 0 | 30 | 142 | 3 | 0 | 19 | 194 | 0.1546 | 0.732 | 0.0155 | 0 | 0.0979 |
| 4 | August | 2 | Feed | adult | Bold | Bold | 2 | 3 | 4.3 | 4m3m2 | 4.2.3 | 0 | 0 | 201 | 0 | 0 | 0 | 201 | 0 | 1 | 0 | 0 | 0 |
| 4 | August | 3 | Feed | adult | Bold | Bold | 1 | 3 | 4.3 | 4m3m3 | 4.1.3 | 0 | 24 | 110 | 10 | 0 | 53 | 197 | 0.1218 | 0.5584 | 0.0508 | 0 | 0.269 |
| 4 | August | 3 | Feed | adult | Bold | Bold | 2 | 3 | 4.3 | 4m3m3 | 4.2.3 | 0 | 12 | 123 | 6 | 0 | 60 | 201 | 0.0597 | 0.6119 | 0.0299 | 0 | 0.2985 |
| 4 | August | 4 | Feed | adult | Bold | Bold | 1 | 3 | 4.3 | 4m3m4 | 4.1.3 | 0 | 18 | 74 | 25 | 0 | 21 | 138 | 0.1304 | 0.5362 | 0.1812 | 0 | 0.1522 |
| 4 | August | 4 | Feed | adult | Bold | Bold | 2 | 3 | 4.3 | 4m3m4 | 4.2.3 | 0 | 9 | 23 | 36 | 0 | 17 | 85 | 0.1059 | 0.2706 | 0.4235 | 0 | 0.2 |
| 4 | August | 5 | Feed | adult | Bold | Bold | 1 | 3 | 4.3 | 4m3m5 | 4.1.3 | 0 | 30 | 90 | 12 | 37 | 13 | 182 | 0.1648 | 0.4945 | 0.0659 | 0.2033 | 0.0714 |
| 4 | August | 5 | Feed | adult | Bold | Bold | 2 | 3 | 4.3 | 4m3m5 | 4.2.3 | 0 | 13 | 58 | 12 | 9 | 19 | 111 | 0.1171 | 0.5225 | 0.1081 | 0.0811 | 0.1712 |
| 4 | August | 1 | Feed | adult | Bold | Bold | 1 | 4 | 4.4 | 4m4m1 | 4.1.4 | 0 | 71 | 41 | 0 | 13 | 67 | 192 | 0.3698 | 0.2135 | 0 | 0.0677 | 0.349 |
| 4 | August | 1 | Feed | adult | Bold | Bold | 2 | 4 | 4.4 | 4m4m1 | 4.2.4 | 0 | 78 | 10 | 0 | 36 | 68 | 192 | 0.4063 | 0.0521 | 0 | 0.1875 | 0.3542 |
| 4 | August | 2 | Feed | adult | Bold | Bold | 1 | 4 | 4.4 | 4m4m2 | 4.1.4 | 0 | 59 | 57 | 0 | 90 | 44 | 250 | 0.236 | 0.228 | 0 | 0.36 | 0.176 |
| 4 | August | 2 | Feed | adult | Bold | Bold | 2 | 4 | 4.4 | 4m4m2 | 4.2.4 | 0 | 25 | 35 | 0 | 43 | 147 | 250 | 0.1 | 0.14 | 0 | 0.172 | 0.588 |
| 4 | August | 3 | Feed | adult | Bold | Bold | 1 | 4 | 4.4 | 4m4m3 | 4.1.4 | 0 | 61 | 46 | 0 | 37 | 71 | 215 | 0.2837 | 0.214 | 0 | 0.1721 | 0.3302 |
| 4 | August | 3 | Feed | adult | Bold | Bold | 2 | 4 | 4.4 | 4m4m3 | 4.2.4 | 0 | 129 | 26 | 0 | 34 | 81 | 270 | 0.4778 | 0.0963 | 0 | 0.1259 | 0.3 |
| 4 | August | 4 | Feed | adult | Bold | Bold | 1 | 4 | 4.4 | 4m4m4 | 4.1.4 | 0 | 20 | 65 | 0 | 13 | 40 | 138 | 0.1449 | 0.471 | 0 | 0.0942 | 0.2899 |
| 4 | August | 4 | Feed | adult | Bold | Bold | 2 | 4 | 4.4 | 4m4m4 | 4.2.4 | 0 | 88 | 41 | 0 | 12 | 96 | 237 | 0.3713 | 0.173 | 0 | 0.0506 | 0.4051 |
| 4 | August | 5 | Feed | adult | Bold | Bold | 1 | 4 | 4.4 | 4m4m5 | 4.1.4 | 0 | 16 | 115 | 0 | 14 | 0 | 145 | 0.1103 | 0.7931 | 0 | 0.0966 | 0 |
| 4 | August | 5 | Feed | adult | Bold | Bold | 2 | 4 | 4.4 | 4m4m5 | 4.2.4 | 0 | 74 | 0 | 0 | 31 | 169 | 274 | 0.2701 | 0 | 0 | 0.1131 | 0.6168 |
| 4 | August | 8 | Feed | adult | Bold | Bold | 1 | 4 | 4.4 | 4m4m8 | 4.1.4 | 0 | 15 | 93 | 0 | 0 | 106 | 214 | 0.0701 | 0.4346 | 0 | 0 | 0.4953 |
| 4 | August | 8 | Feed | adult | Bold | Bold | 2 | 4 | 4.4 | 4m4m8 | 4.2.4 | 0 | 9 | 52 | 0 | 4 | 149 | 214 | 0.0421 | 0.243 | 0 | 0.0187 | 0.6963 |
| 4 | August | 9 | Feed | adult | Bold | Bold | 1 | 4 | 4.4 | 4m4m9 | 4.1.4 | 0 | 52 | 47 | 0 | 3 | 106 | 208 | 0.25 | 0.226 | 0 | 0.0144 | 0.5096 |
| 4 | August | 9 | Feed | adult | Bold | Bold | 2 | 4 | 4.4 | 4m4m9 | 4.2.4 | 0 | 62 | 31 | 0 | 4 | 111 | 208 | 0.2981 | 0.149 | 0 | 0.0192 | 0.5337 |
| 4 | August | 10 | Feed | adult | Bold | Bold | 1 | 4 | 4.4 | 4m4m10 | 4.1.4 | 0 | 31 | 93 | 0 | 45 | 41 | 210 | 0.1476 | 0.4429 | 0 | 0.2143 | 0.1952 |
| 4 | August | 10 | Feed | adult | Bold | Bold | 2 | 4 | 4.4 | 4m4m10 | 4.2.4 | 0 | 9 | 27 | 0 | 0 | 174 | 210 | 0.0429 | 0.1286 | 0 | 0 | 0.8286 |
| 4 | August | 11 | Feed | adult | Bold | Bold | 1 | 4 | 4.4 | 4m4m11 | 4.1.4 | 0 | 5 | 57 | 0 | 50 | 103 | 215 | 0.0233 | 0.2651 | 0 | 0.2326 | 0.4791 |
| 4 | August | 11 | Feed | adult | Bold | Bold | 2 | 4 | 4.4 | 4m4m11 | 4.2.4 | 0 | 24 | 18 | 0 | 52 | 121 | 215 | 0.1116 | 0.0837 | 0 | 0.2419 | 0.5628 |
| 4 | August | 12 | Feed | adult | Bold | Bold | 1 | 4 | 4.4 | 4m4m12 | 4.1.4 | 0 | 73 | 87 | 0 | 44 | 22 | 226 | 0.323 | 0.385 | 0 | 0.1947 | 0.0973 |
| 4 | August | 12 | Feed | adult | Bold | Bold | 2 | 4 | 4.4 | 4m4m12 | 4.2.4 | 0 | 34 | 81 | 0 | 36 | 75 | 226 | 0.1504 | 0.3584 | 0 | 0.1593 | 0.3319 |
| 4 | August | 1 | Walk | adult | Bold | Bold | 1 | 5 | 4.5 | 4m5m1 | 4.1.5 | 0 | 32 | 153 | 0 | 42 | 0 | 227 | 0.141 | 0.674 | 0 | 0.185 | 0 |
| 4 | August | 1 | Walk | adult | Bold | Bold | 2 | 5 | 4.5 | 4m5m1 | 4.2.5 | 0 | 19 | 12 | 0 | 7 | 189 | 227 | 0.0837 | 0.0529 | 0 | 0.0308 | 0.8326 |
| 4 | August | 2 | Walk | adult | Bold | Bold | 1 | 5 | 4.5 | 4m5m2 | 4.1.5 | 0 | 62 | 100 | 14 | 7 | 33 | 216 | 0.287 | 0.463 | 0.0648 | 0.0324 | 0.1528 |
| 4 | August | 2 | Walk | adult | Bold | Bold | 2 | 5 | 4.5 | 4m5m2 | 4.2.5 | 0 | 25 | 47 | 0 | 54 | 90 | 216 | 0.1157 | 0.2176 | 0 | 0.25 | 0.4167 |
| 4 | August | 3 | Walk | adult | Bold | Bold | 1 | 5 | 4.5 | 4m5m3 | 4.1.5 | 0 | 21 | 138 | 11 | 11 | 44 | 225 | 0.0933 | 0.6133 | 0.0489 | 0.0489 | 0.1956 |
| 4 | August | 3 | Walk | adult | Bold | Bold | 2 | 5 | 4.5 | 4m5m3 | 4.2.5 | 0 | 40 | 0 | 0 | 36 | 149 | 225 | 0.1778 | 0 | 0 | 0.16 | 0.6622 |
| 4 | August | 4 | Walk | adult | Bold | Bold | 1 | 5 | 4.5 | 4m5m4 | 4.1.5 | 0 | 0 | 243 | 0 | 0 | 0 | 243 | 0 | 1 | 0 | 0 | 0 |
| 4 | August | 4 | Walk | adult | Bold | Bold | 2 | 5 | 4.5 | 4m5m4 | 4.2.5 | 0 | 7 | 33 | 0 | 0 | 203 | 243 | 0.0288 | 0.1358 | 0 | 0 | 0.8354 |
| 4 | August | 5 | Walk | adult | Bold | Bold | 1 | 5 | 4.5 | 4m5m5 | 4.1.5 | 0 | 0 | 42 | 0 | 0 | 190 | 232 | 0 | 0.181 | 0 | 0 | 0.819 |
| 4 | August | 5 | Walk | adult | Bold | Bold | 2 | 5 | 4.5 | 4m5m5 | 4.2.5 | 0 | 13 | 9 | 0 | 0 | 210 | 232 | 0.056 | 0.0388 | 0 | 0 | 0.9052 |
| 4 | August | 9 | Walk | adult | Bold | Bold | 1 | 5 | 4.5 | 4m5m9 | 4.1.5 | 0 | 5 | 4 | 0 | 0 | 209 | 218 | 0.0229 | 0.0183 | 0 | 0 | 0.9587 |
| 4 | August | 9 | Walk | adult | Bold | Bold | 2 | 5 | 4.5 | 4m5m9 | 4.2.5 | 0 | 43 | 0 | 0 | 0 | 175 | 218 | 0.1972 | 0 | 0 | 0 | 0.8028 |
| 4 | August | 10 | Walk | adult | Bold | Bold | 1 | 5 | 4.5 | 4m5m10 | 4.1.5 | 0 | 37 | 92 | 0 | 24 | 60 | 213 | 0.1737 | 0.4319 | 0 | 0.1127 | 0.2817 |
| 4 | August | 10 | Walk | adult | Bold | Bold | 2 | 5 | 4.5 | 4m5m10 | 4.2.5 | 0 | 11 | 1 | 0 | 15 | 186 | 213 | 0.0516 | 0.0047 | 0 | 0.0704 | 0.8732 |
| 4 | August | 11 | Walk | adult | Bold | Bold | 1 | 5 | 4.5 | 4m5m11 | 4.1.5 | 0 | 36 | 124 | 0 | 39 | 30 | 229 | 0.1572 | 0.5415 | 0 | 0.1703 | 0.131 |
| 4 | August | 11 | Walk | adult | Bold | Bold | 2 | 5 | 4.5 | 4m5m11 | 4.2.5 | 0 | 28 | 0 | 28 | 8 | 165 | 229 | 0.1223 | 0 | 0.1223 | 0.0349 | 0.7205 |
| 4 | August | 12 | Walk | adult | Bold | Bold | 1 | 5 | 4.5 | 4m5m12 | 4.1.5 | 0 | 54 | 105 | 0 | 44 | 22 | 225 | 0.24 | 0.4667 | 0 | 0.1956 | 0.0978 |
| 4 | August | 12 | Walk | adult | Bold | Bold | 2 | 5 | 4.5 | 4m5m12 | 4.2.5 | 0 | 24 | 14 | 21 | 30 | 136 | 225 | 0.1067 | 0.0622 | 0.0933 | 0.1333 | 0.6044 |
| 4 | August | 1 | Walk | adult | Bold | Bold | 1 | 7 | 4.7 | 4m7m1 | 4.1.7 | 0 | 106 | 46 | 0 | 24 | 46 | 222 | 0.4775 | 0.2072 | 0 | 0.1081 | 0.2072 |
| 4 | August | 1 | Walk | adult | Bold | Bold | 2 | 7 | 4.7 | 4m7m1 | 4.2.7 | 0 | 57 | 0 | 0 | 12 | 153 | 222 | 0.2568 | 0 | 0 | 0.0541 | 0.6892 |
| 4 | August | 2 | Walk | adult | Bold | Bold | 1 | 7 | 4.7 | 4m7m2 | 4.1.7 | 0 | 39 | 14 | 0 | 0 | 181 | 234 | 0.1667 | 0.0598 | 0 | 0 | 0.7735 |
| 4 | August | 2 | Walk | adult | Bold | Bold | 2 | 7 | 4.7 | 4m7m2 | 4.2.7 | 0 | 43 | 33 | 0 | 41 | 117 | 234 | 0.1838 | 0.141 | 0 | 0.1752 | 0.5 |
| 4 | August | 3 | Walk | adult | Bold | Bold | 1 | 7 | 4.7 | 4m7m3 | 4.1.7 | 0 | 65 | 28 | 0 | 86 | 48 | 227 | 0.2863 | 0.1233 | 0 | 0.3789 | 0.2115 |
| 4 | August | 3 | Walk | adult | Bold | Bold | 2 | 7 | 4.7 | 4m7m3 | 4.2.7 | 0 | 131 | 12 | 0 | 31 | 53 | 227 | 0.5771 | 0.0529 | 0 | 0.1366 | 0.2335 |
| 4 | August | 4 | Walk | adult | Bold | Bold | 1 | 7 | 4.7 | 4m7m4 | 4.1.7 | 0 | 117 | 0 | 0 | 0 | 110 | 227 | 0.5154 | 0 | 0 | 0 | 0.4846 |
| 4 | August | 4 | Walk | adult | Bold | Bold | 2 | 7 | 4.7 | 4m7m4 | 4.2.7 | 0 | 92 | 0 | 0 | 0 | 135 | 227 | 0.4053 | 0 | 0 | 0 | 0.5947 |
| 4 | August | 5 | Walk | adult | Bold | Bold | 1 | 7 | 4.7 | 4m7m5 | 4.1.7 | 0 | 98 | 20 | 0 | 0 | 119 | 237 | 0.4135 | 0.0844 | 0 | 0 | 0.5021 |
| 4 | August | 5 | Walk | adult | Bold | Bold | 2 | 7 | 4.7 | 4m7m5 | 4.2.7 | 0 | 24 | 0 | 0 | 0 | 213 | 237 | 0.1013 | 0 | 0 | 0 | 0.8987 |
| 4 | August | 8 | Walk | adult | Bold | Bold | 1 | 7 | 4.7 | 4m7m8 | 4.1.7 | 0 | 24 | 0 | 0 | 0 | 199 | 223 | 0.1076 | 0 | 0 | 0 | 0.8924 |
| 4 | August | 8 | Walk | adult | Bold | Bold | 2 | 7 | 4.7 | 4m7m8 | 4.2.7 | 0 | 11 | 0 | 0 | 0 | 212 | 223 | 0.0493 | 0 | 0 | 0 | 0.9507 |
| 4 | August | 9 | Walk | adult | Bold | Bold | 1 | 7 | 4.7 | 4m7m9 | 4.1.7 | 0 | 25 | 0 | 0 | 2 | 190 | 217 | 0.1152 | 0 | 0 | 0.0092 | 0.8756 |
| 4 | August | 9 | Walk | adult | Bold | Bold | 2 | 7 | 4.7 | 4m7m9 | 4.2.7 | 0 | 5 | 0 | 0 | 26 | 186 | 217 | 0.023 | 0 | 0 | 0.1198 | 0.8571 |
| 4 | August | 10 | Walk | adult | Bold | Bold | 1 | 7 | 4.7 | 4m7m10 | 4.1.7 | 0 | 20 | 0 | 0 | 9 | 190 | 219 | 0.0913 | 0 | 0 | 0.0411 | 0.8676 |

| | | | | | | | | | | | | | | | | | | | | | | | |
|---|---|---|---|---|---|---|---|---|---|---|---|---|---|---|---|---|---|---|---|---|---|---|---|
| 4 | August | 10 | Walk | adult | Bold | Bold | 2 | 7 | 4.7 | 4m7m10 | 4.2.7 | 0 | 9 | 0 | 0 | 0 | 210 | 219 | 0.0411 | 0 | 0 | 0 | 0.9589 |
| 4 | August | 11 | Walk | adult | Bold | Bold | 1 | 7 | 4.7 | 4m7m11 | 4.1.7 | 0 | 9 | 5 | 0 | 0 | 205 | 219 | 0.0411 | 0.0228 | 0 | 0 | 0.9361 |
| 4 | August | 11 | Walk | adult | Bold | Bold | 2 | 7 | 4.7 | 4m7m11 | 4.2.7 | 0 | 13 | 0 | 0 | 0 | 206 | 219 | 0.0594 | 0 | 0 | 0 | 0.9406 |
| 4 | August | 12 | Walk | adult | Bold | Bold | 1 | 7 | 4.7 | 4m7m12 | 4.1.7 | 0 | 21 | 0 | 0 | 0 | 207 | 228 | 0.0921 | 0 | 0 | 0 | 0.9079 |
| 4 | August | 12 | Walk | adult | Bold | Bold | 2 | 7 | 4.7 | 4m7m12 | 4.2.7 | 0 | 20 | 0 | 0 | 22 | 186 | 228 | 0.0877 | 0 | 0 | 0.0965 | 0.8158 |
| 5 | Septembe | 1 | Dog | adult | Bold | Bold | 1 | 1 | 5.1 | 5m1m1 | 5.1.1 | 0 | 0 | 188 | 0 | 8 | 23 | 219 | 0 | 0.8584 | 0 | 0.0365 | 0.105 |
| 5 | Septembe | 1 | Dog | adult | Bold | Bold | 2 | 1 | 5.1 | 5m1m1 | 5.2.1 | 0 | 1 | 0 | 0 | 0 | 218 | 219 | 0.0046 | 0 | 0 | 0 | 0.9954 |
| 5 | Septembe | 2 | Dog | adult | Bold | Bold | 1 | 1 | 5.1 | 5m1m2 | 5.1.1 | 0 | 0 | 209 | 0 | 60 | 0 | 269 | 0 | 0.777 | 0 | 0.223 | 0 |
| 5 | Septembe | 2 | Dog | adult | Bold | Bold | 2 | 1 | 5.1 | 5m1m2 | 5.2.1 | 0 | 10 | 0 | 0 | 0 | 259 | 269 | 0.0372 | 0 | 0 | 0 | 0.9628 |
| 5 | Septembe | 3 | Dog | adult | Bold | Bold | 1 | 1 | 5.1 | 5m1m3 | 5.1.1 | 0 | 11 | 180 | 0 | 43 | 6 | 240 | 0.0458 | 0.75 | 0 | 0.1792 | 0.025 |
| 5 | Septembe | 3 | Dog | adult | Bold | Bold | 2 | 1 | 5.1 | 5m1m3 | 5.2.1 | 0 | 7 | 0 | 0 | 0 | 233 | 240 | 0.0292 | 0 | 0 | 0 | 0.9708 |
| 5 | Septembe | 4 | Dog | adult | Bold | Bold | 1 | 1 | 5.1 | 5m1m4 | 5.1.1 | 0 | 0 | 230 | 0 | 18 | 20 | 268 | 0 | 0.8582 | 0 | 0.0672 | 0.0746 |
| 5 | Septembe | 4 | Dog | adult | Bold | Bold | 2 | 1 | 5.1 | 5m1m4 | 5.2.1 | 0 | 23 | 0 | 0 | 0 | 245 | 268 | 0.0858 | 0 | 0 | 0 | 0.9142 |
| 5 | Septembe | 5 | Dog | adult | Bold | Bold | 1 | 1 | 5.1 | 5m1m5 | 5.1.1 | 0 | 7 | 183 | 3 | 25 | 27 | 245 | 0.0286 | 0.7469 | 0.0122 | 0.102 | 0.1102 |
| 5 | Septembe | 5 | Dog | adult | Bold | Bold | 2 | 1 | 5.1 | 5m1m5 | 5.2.1 | 0 | 13 | 0 | 0 | 0 | 232 | 245 | 0.0531 | 0 | 0 | 0 | 0.9469 |
| 5 | Septembe | 1 | Dog | adult | Shy | Bold | 1 | 2 | 5.2 | 5m2m1 | 5.1.2 | 0 | 1 | 192 | 0 | 0 | 6 | 199 | 0.005 | 0.9648 | 0 | 0 | 0.0302 |
| 5 | Septembe | 1 | Dog | adult | Shy | Bold | 2 | 2 | 5.2 | 5m2m1 | 5.2.2 | 0 | 0 | 52 | 0 | 8 | 2 | 62 | 0 | 0.8387 | 0 | 0.129 | 0.0323 |
| 5 | Septembe | 2 | Dog | adult | Shy | Bold | 1 | 2 | 5.2 | 5m2m2 | 5.1.2 | 0 | 7 | 197 | 0 | 12 | 5 | 221 | 0.0317 | 0.8914 | 0 | 0.0543 | 0.0226 |
| 5 | Septembe | 2 | Dog | adult | Shy | Bold | 2 | 2 | 5.2 | 5m2m2 | 5.2.2 | 0 | 13 | 184 | 0 | 8 | 16 | 221 | 0.0588 | 0.8326 | 0 | 0.0362 | 0.0724 |
| 5 | Septembe | 3 | Dog | adult | Shy | Bold | 1 | 2 | 5.2 | 5m2m3 | 5.1.2 | 0 | 0 | 220 | 0 | 3 | 0 | 223 | 0 | 0.9865 | 0 | 0.0135 | 0 |
| 5 | Septembe | 3 | Dog | adult | Shy | Bold | 2 | 2 | 5.2 | 5m2m3 | 5.2.2 | 0 | 0 | 194 | 0 | 4 | 25 | 223 | 0 | 0.87 | 0 | 0.0179 | 0.1121 |
| 5 | Septembe | 4 | Dog | adult | Shy | Bold | 1 | 2 | 5.2 | 5m2m4 | 5.1.2 | 0 | 0 | 211 | 0 | 4 | 8 | 223 | 0 | 0.9462 | 0 | 0.0179 | 0.0359 |
| 5 | Septembe | 4 | Dog | adult | Shy | Bold | 2 | 2 | 5.2 | 5m2m4 | 5.2.2 | 0 | 0 | 194 | 6 | 6 | 17 | 223 | 0 | 0.87 | 0.0269 | 0.0269 | 0.0762 |
| 5 | Septembe | 5 | Dog | adult | Shy | Bold | 1 | 2 | 5.2 | 5m2m5 | 5.1.2 | 0 | 10 | 180 | 0 | 13 | 25 | 228 | 0.0439 | 0.7895 | 0 | 0.057 | 0.1096 |
| 5 | Septembe | 5 | Dog | adult | Shy | Bold | 2 | 2 | 5.2 | 5m2m5 | 5.2.2 | 0 | 5 | 204 | 7 | 0 | 12 | 228 | 0.0219 | 0.8947 | 0.0307 | 0 | 0.0526 |
| 5 | Septembe | 1 | Feed | adult | Bold | Bold | 1 | 3 | 5.3 | 5m3m1 | 5.1.3 | 0 | 48 | 107 | 6 | 11 | 9 | 181 | 0.2652 | 0.5912 | 0.0331 | 0.0608 | 0.0497 |
| 5 | Septembe | 1 | Feed | adult | Bold | Bold | 2 | 3 | 5.3 | 5m3m1 | 5.2.3 | 0 | 27 | 94 | 0 | 2 | 13 | 136 | 0.1985 | 0.6912 | 0 | 0.0147 | 0.0956 |
| 5 | Septembe | 2 | Feed | adult | Bold | Bold | 1 | 3 | 5.3 | 5m3m2 | 5.1.3 | 0 | 18 | 166 | 12 | 7 | 0 | 203 | 0.0887 | 0.8177 | 0.0591 | 0.0345 | 0 |
| 5 | Septembe | 2 | Feed | adult | Bold | Bold | 2 | 3 | 5.3 | 5m3m2 | 5.2.3 | 0 | 20 | 80 | 13 | 4 | 82 | 199 | 0.1005 | 0.402 | 0.0653 | 0.0201 | 0.4121 |
| 5 | Septembe | 3 | Feed | adult | Bold | Bold | 1 | 3 | 5.3 | 5m3m3 | 5.1.3 | 0 | 54 | 119 | 22 | 7 | 6 | 208 | 0.2596 | 0.5721 | 0.1058 | 0.0337 | 0.0288 |
| 5 | Septembe | 3 | Feed | adult | Bold | Bold | 2 | 3 | 5.3 | 5m3m3 | 5.2.3 | 0 | 48 | 40 | 17 | 9 | 57 | 171 | 0.2807 | 0.2339 | 0.0994 | 0.0526 | 0.3333 |
| 5 | Septembe | 4 | Feed | adult | Bold | Bold | 1 | 3 | 5.3 | 5m3m4 | 5.1.3 | 0 | 13 | 115 | 14 | 12 | 32 | 186 | 0.0699 | 0.6183 | 0.0753 | 0.0645 | 0.172 |
| 5 | Septembe | 4 | Feed | adult | Bold | Bold | 2 | 3 | 5.3 | 5m3m4 | 5.2.3 | 0 | 46 | 29 | 14 | 0 | 102 | 191 | 0.2408 | 0.1518 | 0.0733 | 0 | 0.534 |
| 5 | Septembe | 5 | Feed | adult | Bold | Bold | 1 | 3 | 5.3 | 5m3m5 | 5.1.3 | 0 | 36 | 83 | 32 | 6 | 27 | 184 | 0.1957 | 0.4511 | 0.1739 | 0.0326 | 0.1467 |
| 5 | Septembe | 5 | Feed | adult | Bold | Bold | 2 | 3 | 5.3 | 5m3m5 | 5.2.3 | 0 | 38 | 102 | 27 | 9 | 36 | 212 | 0.1792 | 0.4811 | 0.1274 | 0.0425 | 0.1698 |
| 5 | Septembe | 1 | Feed | adult | Shy | Shy | 1 | 4 | 5.4 | 5m4m1 | 5.1.4 | 0 | 17 | 161 | 0 | 6 | 0 | 184 | 0.0924 | 0.875 | 0 | 0.0326 | 0 |
| 5 | Septembe | 1 | Feed | adult | Shy | Shy | 2 | 4 | 5.4 | 5m4m1 | 5.2.4 | 0 | 3 | 0 | 0 | 0 | 214 | 217 | 0.0138 | 0 | 0 | 0 | 0.9862 |
| 5 | Septembe | 2 | Feed | adult | Shy | Shy | 1 | 4 | 5.4 | 5m4m2 | 5.1.4 | 0 | 10 | 158 | 0 | 7 | 0 | 175 | 0.0571 | 0.9029 | 0 | 0.04 | 0 |
| 5 | Septembe | 2 | Feed | adult | Shy | Shy | 2 | 4 | 5.4 | 5m4m2 | 5.2.4 | 0 | 9 | 0 | 0 | 0 | 206 | 215 | 0.0419 | 0 | 0 | 0 | 0.9581 |
| 5 | Septembe | 3 | Feed | adult | Shy | Shy | 1 | 4 | 5.4 | 5m4m3 | 5.1.4 | 0 | 12 | 146 | 10 | 28 | 0 | 196 | 0.0612 | 0.7449 | 0.051 | 0.1429 | 0 |
| 5 | Septembe | 3 | Feed | adult | Shy | Shy | 2 | 4 | 5.4 | 5m4m3 | 5.2.4 | 0 | 11 | 0 | 0 | 0 | 211 | 222 | 0.0495 | 0 | 0 | 0 | 0.9505 |
| 5 | Septembe | 4 | Feed | adult | Shy | Shy | 1 | 4 | 5.4 | 5m4m4 | 5.1.4 | 0 | 0 | 154 | 6 | 0 | 18 | 178 | 0 | 0.8652 | 0.0337 | 0 | 0.1011 |
| 5 | Septembe | 4 | Feed | adult | Shy | Shy | 2 | 4 | 5.4 | 5m4m4 | 5.2.4 | 0 | 2 | 0 | 0 | 9 | 216 | 227 | 0.0088 | 0 | 0 | 0.0396 | 0.9515 |
| 5 | Septembe | 5 | Feed | adult | Shy | Shy | 1 | 4 | 5.4 | 5m4m5 | 5.1.4 | 0 | 0 | 94 | 52 | 42 | 0 | 188 | 0 | 0.5 | 0.2766 | 0.2234 | 0 |
| 5 | Septembe | 5 | Feed | adult | Shy | Shy | 2 | 4 | 5.4 | 5m4m5 | 5.2.4 | 0 | 6 | 0 | 0 | 0 | 221 | 227 | 0.0264 | 0 | 0 | 0 | 0.9736 |
| 5 | Septembe | 11 | Feed | adult | Shy | Shy | 1 | 4 | 5.4 | 5m4m11 | 5.1.4 | 0 | 10 | 62 | 39 | 48 | 75 | 234 | 0.0427 | 0.265 | 0.1667 | 0.2051 | 0.3205 |
| 5 | Septembe | 11 | Feed | adult | Shy | Shy | 2 | 4 | 5.4 | 5m4m11 | 5.2.4 | 0 | 40 | 19 | 0 | 9 | 166 | 234 | 0.1709 | 0.0812 | 0 | 0.0385 | 0.7094 |
| 5 | Septembe | 12 | Feed | adult | Shy | Shy | 1 | 4 | 5.4 | 5m4m12 | 5.1.4 | 0 | 28 | 21 | 0 | 166 | 29 | 244 | 0.1148 | 0.0861 | 0 | 0.6803 | 0.1189 |
| 5 | Septembe | 12 | Feed | adult | Shy | Shy | 2 | 4 | 5.4 | 5m4m12 | 5.2.4 | 0 | 34 | 0 | 0 | 4 | 206 | 244 | 0.1393 | 0 | 0 | 0.0164 | 0.8443 |
| 5 | Septembe | 1 | Walk | adult | Shy | Shy | 1 | 5 | 5.5 | 5m5m1 | 5.1.5 | 0 | 49 | 80 | 0 | 45 | 40 | 214 | 0.229 | 0.3738 | 0 | 0.2103 | 0.1869 |
| 5 | Septembe | 1 | Walk | adult | Shy | Shy | 2 | 5 | 5.5 | 5m5m1 | 5.2.5 | 0 | 30 | 0 | 0 | 7 | 177 | 214 | 0.1402 | 0 | 0 | 0.0327 | 0.8271 |
| 5 | Septembe | 2 | Walk | adult | Shy | Shy | 1 | 5 | 5.5 | 5m5m2 | 5.1.5 | 0 | 102 | 30 | 0 | 62 | 32 | 226 | 0.4513 | 0.1327 | 0 | 0.2743 | 0.1416 |
| 5 | Septembe | 2 | Walk | adult | Shy | Shy | 2 | 5 | 5.5 | 5m5m2 | 5.2.5 | 0 | 38 | 0 | 0 | 17 | 171 | 226 | 0.1681 | 0 | 0 | 0.0752 | 0.7566 |
| 5 | Septembe | 3 | Walk | adult | Shy | Shy | 1 | 5 | 5.5 | 5m5m3 | 5.1.5 | 0 | 99 | 0 | 0 | 82 | 61 | 242 | 0.4091 | 0 | 0 | 0.3388 | 0.2521 |
| 5 | Septembe | 3 | Walk | adult | Shy | Shy | 2 | 5 | 5.5 | 5m5m3 | 5.2.5 | 0 | 39 | 72 | 0 | 62 | 69 | 242 | 0.1612 | 0.2975 | 0 | 0.2562 | 0.2851 |
| 5 | Septembe | 4 | Walk | adult | Shy | Shy | 1 | 5 | 5.5 | 5m5m4 | 5.1.5 | 0 | 21 | 0 | 0 | 6 | 57 | 84 | 0.25 | 0 | 0 | 0.0714 | 0.6786 |
| 5 | Septembe | 4 | Walk | adult | Shy | Shy | 2 | 5 | 5.5 | 5m5m4 | 5.2.5 | 0 | 32 | 11 | 0 | 18 | 23 | 84 | 0.381 | 0.131 | 0 | 0.2143 | 0.2738 |
| 5 | Septembe | 5 | Walk | adult | Shy | Shy | 1 | 5 | 5.5 | 5m5m5 | 5.1.5 | 0 | 14 | 66 | 0 | 62 | 100 | 242 | 0.0579 | 0.2727 | 0 | 0.2562 | 0.4132 |
| 5 | Septembe | 5 | Walk | adult | Shy | Shy | 2 | 5 | 5.5 | 5m5m5 | 5.2.5 | 0 | 32 | 41 | 0 | 91 | 78 | 242 | 0.1322 | 0.1694 | 0 | 0.376 | 0.3223 |
| 5 | Septembe | 8 | Walk | adult | Shy | Shy | 1 | 5 | 5.5 | 5m5m8 | 5.1.5 | 0 | 22 | 118 | 0 | 72 | 10 | 222 | 0.0991 | 0.5315 | 0 | 0.3243 | 0.045 |
| 5 | Septembe | 8 | Walk | adult | Shy | Shy | 2 | 5 | 5.5 | 5m5m8 | 5.2.5 | 0 | 6 | 3 | 0 | 50 | 163 | 222 | 0.027 | 0.0135 | 0 | 0.2252 | 0.7342 |
| 5 | Septembe | 9 | Walk | adult | Shy | Shy | 1 | 5 | 5.5 | 5m5m9 | 5.1.5 | 0 | 20 | 36 | 0 | 65 | 138 | 259 | 0.0772 | 0.139 | 0 | 0.251 | 0.5328 |
| 5 | Septembe | 9 | Walk | adult | Shy | Shy | 2 | 5 | 5.5 | 5m5m9 | 5.2.5 | 0 | 62 | 119 | 0 | 56 | 22 | 259 | 0.2394 | 0.4595 | 0 | 0.2162 | 0.0849 |
| 5 | Septembe | 10 | Walk | adult | Shy | Shy | 1 | 5 | 5.5 | 5m5m10 | 5.1.5 | 0 | 32 | 65 | 0 | 51 | 93 | 241 | 0.1328 | 0.2697 | 0 | 0.2116 | 0.3859 |
| 5 | Septembe | 10 | Walk | adult | Shy | Shy | 2 | 5 | 5.5 | 5m5m10 | 5.2.5 | 0 | 41 | 57 | 0 | 53 | 90 | 241 | 0.1701 | 0.2365 | 0 | 0.2199 | 0.3734 |
| 5 | Septembe | 11 | Walk | adult | Shy | Shy | 1 | 5 | 5.5 | 5m5m11 | 5.1.5 | 0 | 6 | 111 | 0 | 43 | 79 | 239 | 0.0251 | 0.4644 | 0 | 0.1799 | 0.3305 |
| 5 | Septembe | 11 | Walk | adult | Shy | Shy | 2 | 5 | 5.5 | 5m5m11 | 5.2.5 | 0 | 0 | 91 | 0 | 95 | 53 | 239 | 0 | 0.3808 | 0 | 0.3975 | 0.2218 |
| 5 | Septembe | 12 | Walk | adult | Shy | Shy | 1 | 5 | 5.5 | 5m5m12 | 5.1.5 | 0 | 30 | 102 | 4 | 43 | 67 | 246 | 0.122 | 0.4146 | 0.0163 | 0.1748 | 0.2724 |
| 5 | Septembe | 12 | Walk | adult | Shy | Shy | 2 | 5 | 5.5 | 5m5m12 | 5.2.5 | 0 | 0 | 73 | 0 | 30 | 143 | 246 | 0 | 0.2967 | 0 | 0.122 | 0.5813 |
| 5 | Septembe | 8 | aControl | adult | Shy | Shy | 1 | 8 | 5.8 | 5m8m8 | 5.1.8 | 0 | 29 | 102 | 2 | 47 | 48 | 228 | 0.1272 | 0.4474 | 0.0088 | 0.2061 | 0.2105 |
| 5 | Septembe | 8 | aControl | adult | Shy | Shy | 2 | 8 | 5.8 | 5m8m8 | 5.2.8 | 0 | 6 | 97 | 0 | 20 | 105 | 228 | 0.0263 | 0.4254 | 0 | 0.0877 | 0.4605 |
| 5 | Septembe | 9 | aControl | adult | Shy | Shy | 1 | 8 | 5.8 | 5m8m9 | 5.1.8 | 0 | 4 | 119 | 37 | 59 | 10 | 229 | 0.0175 | 0.5197 | 0.1616 | 0.2576 | 0.0437 |
| 5 | Septembe | 9 | aControl | adult | Shy | Shy | 2 | 8 | 5.8 | 5m8m9 | 5.2.8 | 0 | 0 | 30 | 0 | 21 | 178 | 229 | 0 | 0.131 | 0 | 0.0917 | 0.7773 |
| 5 | Septembe | 11 | aControl | adult | Shy | Shy | 1 | 8 | 5.8 | 5m8m11 | 5.1.8 | 0 | 53 | 90 | 3 | 44 | 57 | 247 | 0.2146 | 0.3644 | 0.0121 | 0.1781 | 0.2308 |
| 5 | Septembe | 11 | aControl | adult | Shy | Shy | 2 | 8 | 5.8 | 5m8m11 | 5.2.8 | 0 | 0 | 49 | 0 | 32 | 166 | 247 | 0 | 0.1984 | 0 | 0.1296 | 0.6721 |
| 5 | Septembe | 12 | aControl | adult | Shy | Shy | 1 | 8 | 5.8 | 5m8m12 | 5.1.8 | 0 | 37 | 48 | 9 | 53 | 101 | 248 | 0.1492 | 0.1935 | 0.0363 | 0.2137 | 0.4073 |
| 5 | Septembe | 12 | aControl | adult | Shy | Shy | 2 | 8 | 5.8 | 5m8m12 | 5.2.8 | 0 | 62 | 2 | 0 | 65 | 119 | 248 | 0.25 | 0.0081 | 0 | 0.2621 | 0.4798 |
| 1 | April | 8 | Dog | adult | Shy | Shy | 2 | 2 | 1.2 | 1m2m8 | 1.2.2 | 1 | 17 | 83 | 0 | 80 | 13 | 193 | 0.0881 | 0.4301 | 0 | 0.4145 | 0.0674 |
| 1 | April | 10 | Dog | adult | Shy | Shy | 1 | 2 | 1.2 | 1m2m10 | 1.1.2 | 1 | 33 | 32 | 0 | 20 | 153 | 238 | 0.1387 | 0.1345 | 0 | 0.084 | 0.6429 |
| 1 | April | 8 | Walk | adult | Bold | Bold | 1 | 7 | 1.7 | 1m7m8 | 1.1.7 | 1 | 15 | 41 | 0 | 25 | 94 | 175 | 0.0857 | 0.2343 | 0 | 0.1429 | 0.5371 |
| 1 | April | 9 | Walk | adult | Bold | Bold | 1 | 7 | 1.7 | 1m7m9 | 1.1.7 | 1 | 27 | 36 | 17 | 29 | 124 | 233 | 0.1159 | 0.1545 | 0.073 | 0.1245 | 0.5322 |
| 2 | June | 11 | ChildFeed | child | Bold | Bold | 1 | 1 | 2.1 | 2m1m11 | 2.1.1 | 1 | 60 | 21 | 25 | 58 | 14 | 178 | 0.3371 | 0.118 | 0.1404 | 0.3258 | 0.0787 |
| 2 | June | 11 | ChildFeed | child | Bold | Bold | 2 | 1 | 2.1 | 2m1m11 | 2.2.1 | 1 | 49 | 18 | 16 | 56 | 39 | 178 | 0.2753 | 0.1011 | 0.0899 | 0.3146 | 0.2191 |
| 2 | June | 10 | ChildFeed | child | Bold | Bold | 1 | 2 | 2.2 | 2m2m10 | 2.1.2 | 1 | 43 | 53 | 11 | 134 | 0 | 241 | 0.1784 | 0.2199 | 0.0456 | 0.556 | 0 |
| 2 | June | 10 | ChildFeed | child | Bold | Bold | 2 | 2 | 2.2 | 2m2m10 | 2.2.2 | 1 | 127 | 35 | 15 | 49 | 15 | 241 | 0.527 | 0.1452 | 0.0622 | 0.2033 | 0.0622 |
| 2 | June | 11 | ChildFeed | child | Bold | Bold | 1 | 2 | 2.2 | 2m2m11 | 2.1.2 | 1 | 77 | 68 | 0 | 73 | 0 | 218 | 0.3532 | 0.3119 | 0 | 0.3349 | 0 |
| 2 | June | 11 | ChildFeed | child | Bold | Bold | 2 | 2 | 2.2 | 2m2m11 | 2.2.2 | 1 | 34 | 68 | 0 | 91 | 25 | 218 | 0.156 | 0.3119 | 0 | 0.4174 | 0.1147 |
| 3 | July | 12 | ChildFeed | child | Bold | Bold | 1 | 1 | 3.1 | 3m1m12 | 3.1.1 | 1 | 64 | 13 | 94 | 60 | 83 | 314 | 0.2038 | 0.0414 | 0.2994 | 0.1911 | 0.2643 |
| 3 | July | 12 | ChildFeed | child | Bold | Bold | 2 | 1 | 3.1 | 3m1m12 | 3.2.1 | 1 | 15 | 140 | 0 | 98 | 61 | 314 | 0.0478 | 0.4459 | 0 | 0.3121 | 0.1943 |
| 3 | July | 9 | ChildFeed | child | Bold | Bold | 1 | 2 | 3.2 | 3m2m9 | 3.1.2 | 1 | 130 | 87 | 0 | 62 | 0 | 279 | 0.4659 | 0.3118 | 0 | 0.2222 | 0 |
| 3 | July | 9 | ChildFeed | child | Bold | Bold | 2 | 2 | 3.2 | 3m2m9 | 3.2.2 | 1 | 97 | 68 | 9 | 85 | 20 | 279 | 0.3477 | 0.2437 | 0.0323 | 0.3047 | 0.0717 |
| 3 | July | 12 | ChildFeed | child | Bold | Bold | 1 | 3 | 3.3 | 3m3m12 | 3.1.3 | 1 | 37 | 127 | 0 | 150 | 0 | 314 | 0.1178 | 0.4045 | 0 | 0.4777 | 0 |
| 3 | July | 12 | ChildFeed | child | Bold | Bold | 2 | 3 | 3.3 | 3m3m12 | 3.2.3 | 1 | 51 | 121 | 0 | 69 | 73 | 314 | 0.1624 | 0.3854 | 0 | 0.2197 | 0.2325 |

| | | | | | | | | | | | | | | | | | | | | | | | |
|---|---|---|---|---|---|---|---|---|---|---|---|---|---|---|---|---|---|---|---|---|---|---|---|
| 3 | July | 11 | aChildaControl | child | boldshy | Bold | 1 | 7 | 3.7 | 3m7m11 | 3.1.7 | 1 | 30 | 155 | 27 | 58 | 20 | 290 | 0.1034 | 0.5345 | 0.0931 | 0.2 | 0.069 |
| 3 | July | 11 | aChildaControl | child | boldshy | Bold | 2 | 7 | 3.7 | 3m7m11 | 3.2.7 | 1 | 17 | 138 | 29 | 106 | 0 | 290 | 0.0586 | 0.4759 | 0.1 | 0.3655 | 0 |
| 3 | July | 8 | ChildWalk | child | Bold | Bold | 1 | 8 | 3.8 | 3m8m8 | 3.1.8 | 1 | 2 | 260 | 9 | 46 | 0 | 317 | 0.0063 | 0.8202 | 0.0284 | 0.1451 | 0 |
| 3 | July | 8 | ChildWalk | child | Bold | Bold | 2 | 8 | 3.8 | 3m8m8 | 3.2.8 | 1 | 95 | 173 | 0 | 43 | 6 | 317 | 0.2997 | 0.5457 | 0 | 0.1356 | 0.0189 |
| 3 | July | 11 | ChildWalk | child | Bold | Bold | 1 | 8 | 3.8 | 3m8m11 | 3.1.8 | 1 | 52 | 38 | 172 | 26 | 0 | 288 | 0.1806 | 0.1319 | 0.5972 | 0.0903 | 0 |
| 3 | July | 11 | ChildWalk | child | Bold | Bold | 2 | 8 | 3.8 | 3m8m11 | 3.2.8 | 1 | 4 | 0 | 163 | 44 | 77 | 288 | 0.0139 | 0 | 0.566 | 0.1528 | 0.2674 |
| 4 | August | 12 | Dog | adult | Bold | Bold | 1 | 2 | 4.2 | 4m2m12 | 4.1.2 | 1 | 38 | 89 | 10 | 27 | 56 | 220 | 0.1727 | 0.4045 | 0.0455 | 0.1227 | 0.2545 |
| 4 | August | 12 | Dog | adult | Bold | Bold | 2 | 2 | 4.2 | 4m2m12 | 4.2.2 | 1 | 23 | 16 | 0 | 16 | 165 | 220 | 0.1045 | 0.0727 | 0 | 0.0727 | 0.75 |
| 5 | ieptembe | 10 | Feed | adult | Bold | Bold | 1 | 3 | 5.3 | 5m3m10 | 5.1.3 | 1 | 19 | 144 | 17 | 30 | 16 | 226 | 0.0841 | 0.6372 | 0.0752 | 0.1327 | 0.0708 |
| 5 | ieptembe | 10 | Feed | adult | Bold | Bold | 2 | 3 | 5.3 | 5m3m10 | 5.2.3 | 1 | 8 | 86 | 5 | 17 | 110 | 226 | 0.0354 | 0.3805 | 0.0221 | 0.0752 | 0.4867 |
| 5 | ieptembe | 12 | Feed | adult | Bold | Bold | 1 | 3 | 5.3 | 5m3m12 | 5.1.3 | 1 | 18 | 83 | 5 | 8 | 119 | 233 | 0.0773 | 0.3562 | 0.0215 | 0.0343 | 0.5107 |
| 5 | ieptembe | 12 | Feed | adult | Bold | Bold | 2 | 3 | 5.3 | 5m3m12 | 5.2.3 | 1 | 12 | 183 | 13 | 22 | 3 | 233 | 0.0515 | 0.7854 | 0.0558 | 0.0944 | 0.0129 |
| 5 | ieptembe | 9 | Feed | adult | Shy | Shy | 1 | 4 | 5.4 | 5m4m9 | 5.1.4 | 1 | 15 | 8 | 8 | 194 | 0 | 225 | 0.0667 | 0.0356 | 0.0356 | 0.8622 | 0 |
| 5 | ieptembe | 9 | Feed | adult | Shy | Shy | 2 | 4 | 5.4 | 5m4m9 | 5.2.4 | 1 | 21 | 18 | 0 | 29 | 157 | 225 | 0.0933 | 0.08 | 0 | 0.1289 | 0.6978 |
| 5 | ieptembe | 10 | Feed | adult | Shy | Shy | 1 | 4 | 5.4 | 5m4m10 | 5.1.4 | 1 | 3 | 48 | 8 | 136 | 47 | 242 | 0.0124 | 0.1983 | 0.0331 | 0.562 | 0.1942 |
| 5 | ieptembe | 10 | Feed | adult | Shy | Shy | 2 | 4 | 5.4 | 5m4m10 | 5.2.4 | 1 | 29 | 17 | 0 | 0 | 196 | 242 | 0.1198 | 0.0702 | 0 | 0 | 0.8099 |
| 5 | ieptembe | 10 | aControl | adult | Shy | Shy | 1 | 8 | 5.8 | 5m8m10 | 5.1.8 | 1 | 3 | 23 | 0 | 46 | 159 | 231 | 0.013 | 0.0996 | 0 | 0.1991 | 0.6883 |
| 5 | ieptembe | 10 | aControl | adult | Shy | Shy | 2 | 8 | 5.8 | 5m8m10 | 5.2.8 | 1 | 0 | 79 | 0 | 43 | 109 | 231 | 0 | 0.342 | 0 | 0.1861 | 0.4719 |
| 1 | April | 8 | Dog | adult | Bold | Bold | 1 | 1 | 1.1 | 1m1m8 | 1.1.1 | 2 | 18 | 137 | 2 | 68 | 12 | 237 | 0.0759 | 0.5781 | 0.0084 | 0.2869 | 0.0506 |
| 1 | April | 11 | Dog | adult | Bold | Bold | 1 | 1 | 1.1 | 1m1m11 | 1.1.1 | 2 | 5 | 130 | 7 | 56 | 34 | 232 | 0.0216 | 0.5603 | 0.0302 | 0.2414 | 0.1466 |
| 1 | April | 11 | Dog | adult | Bold | Bold | 2 | 1 | 1.1 | 1m1m11 | 1.2.1 | 2 | 8 | 139 | 0 | 57 | 28 | 232 | 0.0345 | 0.5991 | 0 | 0.2457 | 0.1207 |
| 1 | April | 12 | Dog | adult | Bold | Bold | 2 | 1 | 1.1 | 1m1m12 | 1.2.1 | 2 | 128 | 43 | 25 | 66 | 26 | 288 | 0.4444 | 0.1493 | 0.0868 | 0.2292 | 0.0903 |
| 1 | April | 9 | Dog | adult | Shy | Shy | 2 | 2 | 1.2 | 1m2m9 | 1.2.2 | 2 | 34 | 96 | 0 | 25 | 62 | 217 | 0.1567 | 0.4424 | 0 | 0.1152 | 0.2857 |
| 1 | April | 11 | Dog | adult | Shy | Shy | 2 | 2 | 1.2 | 1m2m11 | 1.2.2 | 2 | 16 | 123 | 0 | 34 | 51 | 224 | 0.0714 | 0.5491 | 0 | 0.1518 | 0.2277 |
| 1 | April | 9 | Feed | adult | Bold | Bold | 2 | 4 | 1.4 | 1m4m9 | 1.2.4 | 2 | 61 | 8 | 2 | 11 | 171 | 253 | 0.2411 | 0.0316 | 0.0079 | 0.0435 | 0.6759 |
| 1 | April | 11 | Feed | adult | Bold | Bold | 2 | 4 | 1.4 | 1m4m11 | 1.2.4 | 2 | 34 | 49 | 0 | 13 | 145 | 241 | 0.1411 | 0.2033 | 0 | 0.0539 | 0.6017 |
| 1 | April | 10 | Walk | adult | Bold | Bold | 1 | 7 | 1.7 | 1m7m10 | 1.1.7 | 2 | 19 | 86 | 0 | 26 | 110 | 241 | 0.0788 | 0.3568 | 0 | 0.1079 | 0.4564 |
| 1 | April | 11 | Walk | adult | Bold | Bold | 2 | 7 | 1.7 | 1m7m11 | 1.2.7 | 2 | 31 | 66 | 0 | 33 | 110 | 240 | 0.1292 | 0.275 | 0 | 0.1375 | 0.4583 |
| 2 | June | 12 | ChildWalk | child | Bold | Bold | 1 | 5 | 2.5 | 2m5m12 | 2.1.5 | 2 | 37 | 0 | 0 | 229 | 0 | 266 | 0.1391 | 0 | 0 | 0.8609 | 0 |
| 2 | June | 12 | ChildWalk | child | Bold | Bold | 2 | 5 | 2.5 | 2m5m12 | 2.2.5 | 2 | 0 | 0 | 0 | 266 | 0 | 266 | 0 | 0 | 0 | 1 | 0 |
| 3 | July | 9 | ChildFeed | child | Bold | Bold | 1 | 3 | 3.3 | 3m3m9 | 3.1.3 | 2 | 8 | 75 | 8 | 209 | 0 | 300 | 0.0267 | 0.25 | 0.0267 | 0.6967 | 0 |
| 3 | July | 9 | ChildFeed | child | Bold | Bold | 2 | 3 | 3.3 | 3m3m9 | 3.2.3 | 2 | 68 | 26 | 16 | 145 | 45 | 300 | 0.2267 | 0.0867 | 0.0533 | 0.4833 | 0.15 |
| 3 | July | 11 | ChildFeed | child | Bold | Bold | 1 | 3 | 3.3 | 3m3m11 | 3.1.3 | 2 | 106 | 47 | 71 | 103 | 0 | 327 | 0.3242 | 0.1437 | 0.2171 | 0.315 | 0 |
| 3 | July | 11 | ChildFeed | child | Bold | Bold | 2 | 3 | 3.3 | 3m3m11 | 3.2.3 | 2 | 106 | 0 | 31 | 117 | 73 | 327 | 0.3242 | 0 | 0.0948 | 0.3578 | 0.2232 |
| 3 | July | 9 | ChildWalk | child | Bold | Bold | 1 | 4 | 3.4 | 3m4m9 | 3.1.4 | 2 | 30 | 91 | 12 | 167 | 0 | 300 | 0.1 | 0.3033 | 0.04 | 0.5567 | 0 |
| 3 | July | 9 | ChildWalk | child | Bold | Bold | 2 | 4 | 3.4 | 3m4m9 | 3.2.4 | 2 | 39 | 21 | 7 | 229 | 4 | 300 | 0.13 | 0.07 | 0.0233 | 0.7633 | 0.0133 |
| 3 | July | 8 | ChildWalk | child | boldshy | Bold | 1 | 5 | 3.5 | 3m5m8 | 3.1.5 | 2 | 0 | 99 | 21 | 38 | 77 | 235 | 0 | 0.4213 | 0.0894 | 0.1617 | 0.3277 |
| 3 | July | 8 | ChildWalk | child | boldshy | Bold | 2 | 5 | 3.5 | 3m5m8 | 3.2.5 | 2 | 24 | 103 | 0 | 44 | 64 | 235 | 0.1021 | 0.4383 | 0 | 0.1872 | 0.2723 |
| 5 | ieptembe | 11 | Dog | adult | Shy | Bold | 1 | 2 | 5.2 | 5m2m11 | 5.1.2 | 2 | 0 | 213 | 0 | 6 | 0 | 219 | 0 | 0.9726 | 0 | 0.0274 | 0 |
| 5 | ieptembe | 11 | Dog | adult | Shy | Bold | 2 | 2 | 5.2 | 5m2m11 | 5.2.2 | 2 | 0 | 80 | 0 | 16 | 123 | 219 | 0 | 0.3653 | 0 | 0.0731 | 0.5616 |
| 5 | ieptembe | 11 | Feed | adult | Bold | Bold | 1 | 3 | 5.3 | 5m3m11 | 5.1.3 | 2 | 13 | 87 | 32 | 18 | 75 | 225 | 0.0578 | 0.3867 | 0.1422 | 0.08 | 0.3333 |
| 5 | ieptembe | 11 | Feed | adult | Bold | Bold | 2 | 3 | 5.3 | 5m3m11 | 5.2.3 | 2 | 10 | 125 | 40 | 23 | 27 | 225 | 0.0444 | 0.5556 | 0.1778 | 0.1022 | 0.12 |
| 1 | April | 10 | Dog | adult | Bold | Bold | 1 | 1 | 1.1 | 1m1m10 | 1.1.1 | 3 | 18 | 115 | 7 | 58 | 44 | 242 | 0.0744 | 0.4752 | 0.0289 | 0.2397 | 0.1818 |
| 1 | April | 12 | Dog | adult | Shy | Shy | 1 | 2 | 1.2 | 1m2m12 | 1.1.2 | 3 | 31 | 8 | 0 | 14 | 164 | 217 | 0.1429 | 0.0369 | 0 | 0.0645 | 0.7558 |
| 1 | April | 12 | Dog | adult | Shy | Shy | 2 | 2 | 1.2 | 1m2m12 | 1.2.2 | 3 | 31 | 25 | 0 | 20 | 141 | 217 | 0.1429 | 0.1152 | 0 | 0.0922 | 0.6498 |
| 1 | April | 12 | aControl | adult | Shy | Shy | 2 | 8 | 1.8 | 1m8m12 | 1.2.8 | 3 | 9 | 59 | 0 | 43 | 117 | 228 | 0.0395 | 0.2588 | 0 | 0.1886 | 0.5132 |
| 2 | June | 12 | ChildWalk | child | Bold | Bold | 1 | 6 | 2.6 | 2m6m12 | 2.1.6 | 3 | 0 | 214 | 0 | 8 | 0 | 222 | 0 | 0.964 | 0 | 0.036 | 0 |
| 2 | June | 12 | ChildWalk | child | Bold | Bold | 2 | 6 | 2.6 | 2m6m12 | 2.2.6 | 3 | 33 | 7 | 0 | 126 | 56 | 222 | 0.1486 | 0.0315 | 0 | 0.5676 | 0.2523 |
| 3 | July | 10 | ChildFeed | child | Bold | Bold | 1 | 3 | 3.3 | 3m3m10 | 3.1.3 | 3 | 102 | 84 | 48 | 64 | 4 | 302 | 0.3377 | 0.2781 | 0.1589 | 0.2119 | 0.0132 |
| 3 | July | 10 | ChildFeed | child | Bold | Bold | 2 | 3 | 3.3 | 3m3m10 | 3.2.3 | 3 | 60 | 13 | 3 | 38 | 188 | 302 | 0.1987 | 0.043 | 0.0099 | 0.1258 | 0.6225 |
| 3 | July | 10 | ChildWalk | child | Bold | Bold | 1 | 4 | 3.4 | 3m4m10 | 3.1.4 | 3 | 76 | 109 | 33 | 96 | 17 | 331 | 0.2296 | 0.3293 | 0.0997 | 0.29 | 0.0514 |
| 3 | July | 10 | ChildWalk | child | Bold | Bold | 2 | 4 | 3.4 | 3m4m10 | 3.2.4 | 3 | 33 | 52 | 16 | 56 | 174 | 331 | 0.0997 | 0.1571 | 0.0483 | 0.1692 | 0.5257 |
| 4 | August | 10 | Dog | adult | Bold | Bold | 1 | 1 | 4.1 | 4m1m10 | 4.1.1 | 3 | 9 | 77 | 13 | 11 | 92 | 202 | 0.0446 | 0.3812 | 0.0644 | 0.0545 | 0.4554 |
| 5 | ieptembe | 9 | Feed | adult | Bold | Bold | 1 | 3 | 5.3 | 5m3m9 | 5.1.3 | 3 | 57 | 79 | 15 | 46 | 24 | 221 | 0.2579 | 0.3575 | 0.0679 | 0.2081 | 0.1086 |
| 5 | ieptembe | 9 | Feed | adult | Bold | Bold | 2 | 3 | 5.3 | 5m3m9 | 5.2.3 | 3 | 42 | 89 | 32 | 26 | 32 | 221 | 0.19 | 0.4027 | 0.1448 | 0.1176 | 0.1448 |
| 1 | April | 9 | Dog | adult | Bold | Bold | 1 | 1 | 1.1 | 1m1m9 | 1.1.1 | 4 | 25 | 127 | 6 | 50 | 28 | 236 | 0.1059 | 0.5381 | 0.0254 | 0.2119 | 0.1186 |
| 1 | April | 9 | Dog | adult | Bold | Bold | 2 | 1 | 1.1 | 1m1m9 | 1.2.1 | 4 | 14 | 110 | 26 | 49 | 37 | 236 | 0.0593 | 0.4661 | 0.1102 | 0.2076 | 0.1568 |
| 1 | April | 11 | aControl | adult | Shy | Shy | 1 | 8 | 1.8 | 1m8m11 | 1.1.8 | 4 | 5 | 56 | 0 | 8 | 168 | 237 | 0.0211 | 0.2363 | 0 | 0.0338 | 0.7089 |
| 2 | June | 8 | ChildFeed | child | Bold | Bold | 1 | 2 | 2.2 | 2m2m8 | 2.1.2 | 4 | 61 | 22 | 0 | 72 | 66 | 221 | 0.276 | 0.0995 | 0 | 0.3258 | 0.2986 |
| 2 | June | 8 | ChildFeed | child | Bold | Bold | 2 | 2 | 2.2 | 2m2m8 | 2.2.2 | 4 | 142 | 51 | 8 | 20 | 0 | 221 | 0.6425 | 0.2308 | 0.0362 | 0.0905 | 0 |
| 3 | July | 10 | aChildaControl | child | boldshy | Bold | 1 | 7 | 3.7 | 3m7m10 | 3.1.7 | 4 | 26 | 155 | 11 | 117 | 8 | 317 | 0.082 | 0.489 | 0.0347 | 0.3691 | 0.0252 |
| 3 | July | 10 | aChildaControl | child | boldshy | Bold | 2 | 7 | 3.7 | 3m7m10 | 3.2.7 | 4 | 21 | 171 | 33 | 92 | 0 | 317 | 0.0662 | 0.5394 | 0.1041 | 0.2902 | 0 |
| 3 | July | 9 | ChildWalk | child | Bold | Bold | 1 | 8 | 3.8 | 3m8m9 | 3.1.8 | 4 | 52 | 194 | 14 | 76 | 0 | 336 | 0.1548 | 0.5774 | 0.0417 | 0.2262 | 0 |
| 3 | July | 9 | ChildWalk | child | Bold | Bold | 2 | 8 | 3.8 | 3m8m9 | 3.2.8 | 4 | 27 | 157 | 26 | 126 | 0 | 336 | 0.0804 | 0.4673 | 0.0774 | 0.375 | 0 |
| 4 | August | 10 | Feed | adult | Bold | Bold | 1 | 3 | 4.3 | 4m3m10 | 4.1.3 | 4 | 8 | 23 | 39 | 143 | 0 | 213 | 0.0376 | 0.108 | 0.1831 | 0.6714 | 0 |
| 4 | August | 10 | Feed | adult | Bold | Bold | 2 | 3 | 4.3 | 4m3m10 | 4.2.3 | 4 | 8 | 28 | 14 | 163 | 0 | 213 | 0.0376 | 0.1315 | 0.0657 | 0.7653 | 0 |
| 4 | August | 11 | Feed | adult | Bold | Bold | 1 | 3 | 4.3 | 4m3m11 | 4.1.3 | 4 | 36 | 24 | 3 | 64 | 89 | 216 | 0.1667 | 0.1111 | 0.0139 | 0.2963 | 0.412 |
| 4 | August | 11 | Feed | adult | Bold | Bold | 2 | 3 | 4.3 | 4m3m11 | 4.2.3 | 4 | 32 | 37 | 18 | 72 | 57 | 216 | 0.1481 | 0.1713 | 0.0833 | 0.3333 | 0.2639 |
| 4 | August | 8 | Walk | adult | Bold | Bold | 1 | 5 | 4.5 | 4m5m8 | 4.1.5 | 4 | 9 | 178 | 0 | 22 | 15 | 224 | 0.0402 | 0.7946 | 0 | 0.0982 | 0.067 |
| 4 | August | 8 | Walk | adult | Bold | Bold | 2 | 5 | 4.5 | 4m5m8 | 4.2.5 | 4 | 15 | 0 | 0 | 3 | 206 | 224 | 0.067 | 0 | 0 | 0.0134 | 0.9196 |
| 3 | July | 9 | ChildFeed | child | Bold | Bold | 1 | 1 | 3.1 | 3m1m9 | 3.1.1 | 5 | 31 | 0 | 0 | 101 | 156 | 288 | 0.1076 | 0 | 0 | 0.3507 | 0.5417 |
| 3 | July | 9 | ChildFeed | child | Bold | Bold | 2 | 1 | 3.1 | 3m1m9 | 3.2.1 | 5 | 76 | 80 | 0 | 132 | 0 | 288 | 0.2639 | 0.2778 | 0 | 0.4583 | 0 |
| 4 | August | 11 | Dog | adult | Bold | Bold | 1 | 1 | 4.1 | 4m1m11 | 4.1.1 | 5 | 16 | 64 | 47 | 18 | 78 | 223 | 0.0717 | 0.287 | 0.2108 | 0.0807 | 0.3498 |
| 5 | ieptembe | 12 | Dog | adult | Shy | Bold | 1 | 2 | 5.2 | 5m2m12 | 5.1.2 | 5 | 0 | 70 | 8 | 40 | 130 | 248 | 0 | 0.2823 | 0.0323 | 0.1613 | 0.5242 |
| 5 | ieptembe | 12 | Dog | adult | Shy | Bold | 2 | 2 | 5.2 | 5m2m12 | 5.2.2 | 5 | 0 | 220 | 0 | 16 | 12 | 248 | 0 | 0.8871 | 0 | 0.0645 | 0.0484 |
| 5 | ieptembe | 8 | Feed | adult | Shy | Shy | 1 | 4 | 5.4 | 5m4m8 | 5.1.4 | 5 | 51 | 72 | 3 | 84 | 0 | 210 | 0.2429 | 0.3429 | 0.0143 | 0.4 | 0 |
| 5 | ieptembe | 8 | Feed | adult | Shy | Shy | 2 | 4 | 5.4 | 5m4m8 | 5.2.4 | 5 | 46 | 0 | 0 | 39 | 125 | 210 | 0.219 | 0 | 0 | 0.1857 | 0.5952 |
| 1 | April | 8 | Dog | adult | Bold | Bold | 2 | 1 | 1.1 | 1m1m8 | 1.2.1 | 6 | 12 | 139 | 9 | 49 | 28 | 237 | 0.0506 | 0.5865 | 0.038 | 0.2068 | 0.1181 |
| 1 | April | 10 | Dog | adult | Shy | Shy | 2 | 2 | 1.2 | 1m2m10 | 1.2.2 | 6 | 9 | 143 | 0 | 21 | 65 | 238 | 0.0378 | 0.6008 | 0 | 0.0882 | 0.2731 |
| 1 | April | 11 | aControl | adult | Shy | Shy | 2 | 8 | 1.8 | 1m8m11 | 1.2.8 | 6 | 0 | 150 | 20 | 44 | 23 | 237 | 0 | 0.6329 | 0.0844 | 0.1857 | 0.097 |
| 2 | June | 11 | ChildWalk | child | Bold | Bold | 1 | 6 | 2.6 | 2m6m11 | 2.1.6 | 6 | 12 | 0 | 0 | 84 | 163 | 259 | 0.0463 | 0 | 0 | 0.3243 | 0.6293 |
| 2 | June | 11 | ChildWalk | child | Bold | Bold | 2 | 6 | 2.6 | 2m6m11 | 2.2.6 | 6 | 6 | 135 | 0 | 21 | 97 | 259 | 0.0232 | 0.5212 | 0 | 0.0811 | 0.3745 |
| 3 | July | 10 | ChildFeed | child | Bold | Bold | 1 | 1 | 3.1 | 3m1m10 | 3.1.1 | 6 | 10 | 210 | 22 | 81 | 0 | 323 | 0.031 | 0.6502 | 0.0681 | 0.2508 | 0 |
| 3 | July | 10 | ChildFeed | child | Bold | Bold | 2 | 1 | 3.1 | 3m1m10 | 3.2.1 | 6 | 9 | 6 | 21 | 32 | 255 | 323 | 0.0279 | 0.0186 | 0.065 | 0.0991 | 0.7895 |
| 3 | July | 11 | ChildFeed | child | Bold | Bold | 1 | 1 | 3.1 | 3m1m11 | 3.1.1 | 6 | 103 | 152 | 14 | 81 | 37 | 387 | 0.2661 | 0.3928 | 0.0362 | 0.2093 | 0.0956 |
| 3 | July | 11 | ChildFeed | child | Bold | Bold | 2 | 1 | 3.1 | 3m1m11 | 3.2.1 | 6 | 9 | 203 | 5 | 34 | 136 | 387 | 0.0233 | 0.5245 | 0.0129 | 0.0879 | 0.3514 |
| 4 | August | 9 | Dog | adult | Bold | Bold | 1 | 1 | 4.1 | 4m1m9 | 4.1.1 | 6 | 23 | 93 | 5 | 105 | 15 | 241 | 0.0954 | 0.3859 | 0.0207 | 0.4357 | 0.0622 |
| 1 | April | 10 | Dog | adult | Bold | Bold | 2 | 1 | 1.1 | 1m1m10 | 1.2.1 | 7 | 6 | 159 | 0 | 57 | 20 | 242 | 0.0248 | 0.657 | 0 | 0.2355 | 0.0826 |
| 3 | July | 8 | ChildWalk | child | Bold | Bold | 1 | 4 | 3.4 | 3m4m8 | 3.1.4 | 7 | 67 | 111 | 16 | 94 | 0 | 288 | 0.2326 | 0.3854 | 0.0556 | 0.3264 | 0 |
| 3 | July | 8 | ChildWalk | child | Bold | Bold | 2 | 4 | 3.4 | 3m4m8 | 3.2.4 | 7 | 64 | 121 | 0 | 85 | 18 | 288 | 0.2222 | 0.4201 | 0 | 0.2951 | 0.0625 |
| 4 | August | 12 | Dog | adult | Bold | Bold | 1 | 1 | 4.1 | 4m1m12 | 4.1.1 | 7 | 39 | 129 | 42 | 20 | 12 | 242 | 0.1612 | 0.5331 | 0.1736 | 0.0826 | 0.0496 |
| 1 | April | 9 | Feed | adult | Bold | Bold | 1 | 4 | 1.4 | 1m4m9 | 1.1.4 | 8 | 25 | 20 | 0 | 44 | 164 | 253 | 0.0988 | 0.0791 | 0 | 0.1739 | 0.6482 |
| 1 | April | 11 | Feed | adult | Bold | Bold | 1 | 4 | 1.4 | 1m4m11 | 1.1.4 | 8 | 30 | 51 | 0 | 67 | 93 | 241 | 0.1245 | 0.2116 | 0 | 0.278 | 0.3859 |

| | | | | | | | | | | | | | | | | | | | | | | | |
|---|---|---|---|---|---|---|---|---|---|---|---|---|---|---|---|---|---|---|---|---|---|---|---|
| 2 | June | 8 | ChildFeed | child | Bold | Bold | 1 | 1 | 2.1 | 2m1m8 | 2.1.1 | 8 | 150 | 53 | 3 | 16 | 0 | 222 | 0.6757 | 0.2387 | 0.0135 | 0.0721 | 0 |
| 2 | June | 8 | ChildFeed | child | Bold | Bold | 2 | 1 | 2.1 | 2m1m8 | 2.2.1 | 8 | 181 | 34 | 7 | 0 | 0 | 222 | 0.8153 | 0.1532 | 0.0315 | 0 | 0 |
| 2 | June | 12 | aChildaControl | child | Bold | Bold | 1 | 8 | 2.8 | 2m8m12 | 2.1.8 | 8 | 82 | 61 | 0 | 104 | 10 | 257 | 0.3191 | 0.2374 | 0 | 0.4047 | 0.0389 |
| 2 | June | 12 | aChildaControl | child | Bold | Bold | 2 | 8 | 2.8 | 2m8m12 | 2.2.8 | 8 | 56 | 35 | 0 | 78 | 88 | 257 | 0.2179 | 0.1362 | 0 | 0.3035 | 0.3424 |
| 3 | July | 8 | ChildFeed | child | Bold | Bold | 1 | 3 | 3.3 | 3m3m8 | 3.1.3 | 8 | 94 | 74 | 25 | 59 | 19 | 271 | 0.3469 | 0.2731 | 0.0923 | 0.2177 | 0.0701 |
| 3 | July | 8 | ChildFeed | child | Bold | Bold | 2 | 3 | 3.3 | 3m3m8 | 3.2.3 | 8 | 62 | 64 | 0 | 73 | 72 | 271 | 0.2288 | 0.2362 | 0 | 0.2694 | 0.2657 |
| 4 | August | 12 | Feed | adult | Bold | Bold | 1 | 3 | 4.3 | 4m3m12 | 4.1.3 | 8 | 40 | 55 | 27 | 84 | 16 | 222 | 0.1802 | 0.2477 | 0.1216 | 0.3784 | 0.0721 |
| 4 | August | 12 | Feed | adult | Bold | Bold | 2 | 3 | 4.3 | 4m3m12 | 4.2.3 | 8 | 19 | 29 | 23 | 117 | 34 | 222 | 0.0856 | 0.1306 | 0.1036 | 0.527 | 0.1532 |
| 1 | April | 10 | aControl | adult | Shy | Shy | 1 | 8 | 1.8 | 1m8m10 | 1.1.8 | 9 | 5 | 126 | 9 | 25 | 74 | 239 | 0.0209 | 0.5272 | 0.0377 | 0.1046 | 0.3096 |
| 3 | July | 8 | ChildFeed | child | Bold | Bold | 1 | 1 | 3.1 | 3m1m8 | 3.1.1 | 9 | 66 | 92 | 12 | 64 | 49 | 283 | 0.2332 | 0.3251 | 0.0424 | 0.2261 | 0.1731 |
| 3 | July | 8 | ChildFeed | child | Bold | Bold | 2 | 1 | 3.1 | 3m1m8 | 3.2.1 | 9 | 76 | 135 | 10 | 62 | 0 | 283 | 0.2686 | 0.477 | 0.0353 | 0.2191 | 0 |
| 5 | Septembe | 10 | Dog | adult | Shy | Bold | 1 | 2 | 5.2 | 5m2m10 | 5.1.2 | 9 | 2 | 106 | 0 | 4 | 125 | 237 | 0.0084 | 0.4473 | 0 | 0.0169 | 0.5274 |
| 5 | Septembe | 10 | Dog | adult | Shy | Bold | 2 | 2 | 5.2 | 5m2m10 | 5.2.2 | 9 | 0 | 216 | 0 | 7 | 14 | 237 | 0 | 0.9114 | 0 | 0.0295 | 0.0591 |
| 1 | April | 10 | Feed | adult | Bold | Bold | 1 | 4 | 1.4 | 1m4m10 | 1.1.4 | 11 | 28 | 77 | 8 | 64 | 69 | 246 | 0.1138 | 0.313 | 0.0325 | 0.2602 | 0.2805 |
| 2 | June | 9 | ChildWalk | child | Bold | Bold | 1 | 5 | 2.5 | 2m5m9 | 2.1.5 | 12 | 40 | 151 | 0 | 38 | 76 | 305 | 0.1311 | 0.4951 | 0 | 0.1246 | 0.2492 |
| 2 | June | 9 | ChildWalk | child | Bold | Bold | 2 | 5 | 2.5 | 2m5m9 | 2.2.5 | 12 | 0 | 160 | 0 | 92 | 53 | 305 | 0 | 0.5246 | 0 | 0.3016 | 0.1738 |
| 3 | July | 9 | aChildaControl | child | boldshy | Bold | 1 | 7 | 3.7 | 3m7m9 | 3.1.7 | 12 | 35 | 7 | 21 | 47 | 157 | 267 | 0.1311 | 0.0262 | 0.0787 | 0.176 | 0.588 |
| 3 | July | 9 | aChildaControl | child | boldshy | Bold | 2 | 7 | 3.7 | 3m7m9 | 3.2.7 | 12 | 18 | 130 | 10 | 109 | 0 | 267 | 0.0674 | 0.4869 | 0.0375 | 0.4082 | 0 |
| 3 | July | 11 | ChildWalk | child | Bold | Bold | 1 | 4 | 3.4 | 3m4m11 | 3.1.4 | 14 | 43 | 202 | 6 | 49 | 31 | 331 | 0.1299 | 0.6103 | 0.0181 | 0.148 | 0.0937 |
| 3 | July | 11 | ChildWalk | child | Bold | Bold | 2 | 4 | 3.4 | 3m4m11 | 3.2.4 | 14 | 41 | 73 | 50 | 113 | 54 | 331 | 0.1239 | 0.2205 | 0.1511 | 0.3414 | 0.1631 |
| 4 | August | 9 | Feed | adult | Bold | Bold | 1 | 3 | 4.3 | 4m3m9 | 4.1.3 | 14 | 72 | 58 | 6 | 39 | 34 | 209 | 0.3445 | 0.2775 | 0.0287 | 0.1866 | 0.1627 |
| 4 | August | 9 | Feed | adult | Bold | Bold | 2 | 3 | 4.3 | 4m3m9 | 4.2.3 | 14 | 58 | 76 | 0 | 75 | 0 | 209 | 0.2775 | 0.3636 | 0 | 0.3589 | 0 |
| 2 | June | 10 | ChildWalk | child | Bold | Bold | 1 | 5 | 2.5 | 2m5m10 | 2.1.5 | 15 | 2 | 212 | 0 | 31 | 19 | 264 | 0.0076 | 0.803 | 0 | 0.1174 | 0.072 |
| 2 | June | 10 | ChildWalk | child | Bold | Bold | 2 | 5 | 2.5 | 2m5m10 | 2.2.5 | 15 | 64 | 148 | 0 | 32 | 20 | 264 | 0.2424 | 0.5606 | 0 | 0.1212 | 0.0758 |
| 2 | June | 11 | aChildaControl | child | Bold | Bold | 1 | 8 | 2.8 | 2m8m11 | 2.1.8 | 15 | 42 | 150 | 0 | 44 | 32 | 268 | 0.1567 | 0.5597 | 0 | 0.1642 | 0.1194 |
| 2 | June | 11 | aChildaControl | child | Bold | Bold | 2 | 8 | 2.8 | 2m8m11 | 2.2.8 | 15 | 23 | 45 | 0 | 64 | 136 | 268 | 0.0858 | 0.1679 | 0 | 0.2388 | 0.5075 |
| 3 | July | 8 | aChildaControl | child | boldshy | Bold | 1 | 7 | 3.7 | 3m7m8 | 3.1.7 | 15 | 73 | 77 | 13 | 108 | 27 | 298 | 0.245 | 0.2584 | 0.0436 | 0.3624 | 0.0906 |
| 3 | July | 8 | aChildaControl | child | boldshy | Bold | 2 | 7 | 3.7 | 3m7m8 | 3.2.7 | 15 | 85 | 78 | 0 | 75 | 60 | 298 | 0.2852 | 0.2617 | 0 | 0.2517 | 0.2013 |
| 5 | Septembe | 8 | Feed | adult | Bold | Bold | 1 | 3 | 5.3 | 5m3m8 | 5.1.3 | 15 | 48 | 129 | 20 | 14 | 0 | 211 | 0.2275 | 0.6114 | 0.0948 | 0.0664 | 0 |
| 5 | Septembe | 8 | Feed | adult | Bold | Bold | 2 | 3 | 5.3 | 5m3m8 | 5.2.3 | 15 | 76 | 92 | 18 | 22 | 3 | 211 | 0.3602 | 0.436 | 0.0853 | 0.1043 | 0.0142 |
| 1 | April | 8 | aControl | adult | Shy | Shy | 1 | 8 | 1.8 | 1m8m8 | 1.1.8 | 17 | 11 | 212 | 0 | 20 | 0 | 243 | 0.0453 | 0.8724 | 0 | 0.0823 | 0 |
| 2 | June | 9 | ChildWalk | child | Bold | Bold | 1 | 6 | 2.6 | 2m6m9 | 2.1.6 | 17 | 0 | 245 | 0 | 0 | 0 | 245 | 0 | 1 | 0 | 0 | 0 |
| 2 | June | 9 | ChildWalk | child | Bold | Bold | 2 | 6 | 2.6 | 2m6m9 | 2.2.6 | 17 | 9 | 0 | 0 | 0 | 236 | 245 | 0.0367 | 0 | 0 | 0 | 0.9633 |
| 4 | August | 8 | Dog | adult | Bold | Bold | 1 | 1 | 4.1 | 4m1m8 | 4.1.1 | 17 | 36 | 114 | 7 | 45 | 29 | 231 | 0.1558 | 0.4935 | 0.0303 | 0.1948 | 0.1255 |
| 2 | June | 9 | aChildaControl | child | Bold | Bold | 1 | 8 | 2.8 | 2m8m9 | 2.1.8 | 18 | 0 | 145 | 0 | 4 | 89 | 238 | 0 | 0.6092 | 0 | 0.0168 | 0.3739 |
| 2 | June | 9 | aChildaControl | child | Bold | Bold | 2 | 8 | 2.8 | 2m8m9 | 2.2.8 | 18 | 0 | 80 | 0 | 29 | 129 | 238 | 0 | 0.3361 | 0 | 0.1218 | 0.542 |
| 5 | Septembe | 12 | Dog | adult | Bold | Bold | 1 | 1 | 5.1 | 5m1m12 | 5.1.1 | 19 | 0 | 158 | 0 | 98 | 0 | 256 | 0 | 0.6172 | 0 | 0.3828 | 0 |
| 5 | Septembe | 12 | Dog | adult | Bold | Bold | 2 | 1 | 5.1 | 5m1m12 | 5.2.1 | 19 | 3 | 0 | 0 | 0 | 253 | 256 | 0.0117 | 0 | 0 | 0 | 0.9883 |
| 1 | April | 9 | aControl | adult | Shy | Shy | 1 | 8 | 1.8 | 1m8m9 | 1.1.8 | 20 | 31 | 131 | 0 | 42 | 41 | 245 | 0.1265 | 0.5347 | 0 | 0.1714 | 0.1673 |
| 4 | August | 11 | aControl | adult | Bold | Bold | 1 | 8 | 4.8 | 4m8m11 | 4.1.8 | 20 | 6 | 95 | 6 | 15 | 95 | 217 | 0.0276 | 0.4378 | 0.0276 | 0.0691 | 0.4378 |
| 4 | August | 11 | aControl | adult | Bold | Bold | 2 | 8 | 4.8 | 4m8m11 | 4.2.8 | 20 | 0 | 135 | 4 | 3 | 75 | 217 | 0 | 0.6221 | 0.0184 | 0.0138 | 0.3456 |
| 5 | Septembe | 11 | Dog | adult | Bold | Bold | 1 | 1 | 5.1 | 5m1m11 | 5.1.1 | 21 | 0 | 188 | 3 | 56 | 0 | 247 | 0 | 0.7611 | 0.0121 | 0.2267 | 0 |
| 5 | Septembe | 11 | Dog | adult | Bold | Bold | 2 | 1 | 5.1 | 5m1m11 | 5.2.1 | 21 | 1 | 0 | 0 | 0 | 246 | 247 | 0.004 | 0 | 0 | 0 | 0.996 |
| 2 | June | 8 | ChildWalk | child | Bold | Bold | 1 | 5 | 2.5 | 2m5m8 | 2.1.5 | 22 | 4 | 126 | 15 | 72 | 10 | 227 | 0.0176 | 0.5551 | 0.0661 | 0.3172 | 0.0441 |
| 2 | June | 8 | ChildWalk | child | Bold | Bold | 2 | 5 | 2.5 | 2m5m8 | 2.2.5 | 22 | 0 | 122 | 9 | 46 | 50 | 227 | 0 | 0.5374 | 0.0396 | 0.2026 | 0.2203 |
| 2 | June | 8 | ChildWalk | child | Bold | Bold | 1 | 6 | 2.6 | 2m6m8 | 2.1.6 | 23 | 0 | 243 | 0 | 5 | 0 | 248 | 0 | 0.9798 | 0 | 0.0202 | 0 |
| 2 | June | 8 | ChildWalk | child | Bold | Bold | 2 | 6 | 2.6 | 2m6m8 | 2.2.6 | 23 | 19 | 0 | 0 | 0 | 229 | 248 | 0.0766 | 0 | 0 | 0 | 0.9234 |
| 2 | June | 10 | ChildWalk | child | Bold | Bold | 1 | 6 | 2.6 | 2m6m10 | 2.1.6 | 25 | 6 | 227 | 0 | 14 | 0 | 247 | 0.0243 | 0.919 | 0 | 0.0567 | 0 |
| 2 | June | 10 | ChildWalk | child | Bold | Bold | 2 | 6 | 2.6 | 2m6m10 | 2.2.6 | 25 | 10 | 0 | 0 | 21 | 216 | 247 | 0.0405 | 0 | 0 | 0.085 | 0.8745 |
| 4 | August | 10 | aControl | adult | Bold | Bold | 1 | 8 | 4.8 | 4m8m10 | 4.1.8 | 25 | 2 | 109 | 0 | 16 | 94 | 221 | 0.009 | 0.4932 | 0 | 0.0724 | 0.4253 |
| 4 | August | 10 | aControl | adult | Bold | Bold | 2 | 8 | 4.8 | 4m8m10 | 4.2.8 | 25 | 5 | 125 | 0 | 26 | 65 | 221 | 0.0226 | 0.5656 | 0 | 0.1176 | 0.2941 |
| 5 | Septembe | 10 | Dog | adult | Bold | Bold | 1 | 1 | 5.1 | 5m1m10 | 5.1.1 | 26 | 9 | 155 | 0 | 76 | 3 | 243 | 0.037 | 0.6379 | 0 | 0.3128 | 0.0123 |
| 5 | Septembe | 10 | Dog | adult | Bold | Bold | 2 | 1 | 5.1 | 5m1m10 | 5.2.1 | 26 | 1 | 0 | 0 | 0 | 242 | 243 | 0.0041 | 0 | 0 | 0 | 0.9959 |
| 5 | Septembe | 9 | Dog | adult | Shy | Bold | 1 | 2 | 5.2 | 5m2m9 | 5.1.2 | 31 | 2 | 192 | 0 | 33 | 42 | 269 | 0.0074 | 0.7138 | 0 | 0.1227 | 0.1561 |
| 5 | Septembe | 9 | Dog | adult | Shy | Bold | 2 | 2 | 5.2 | 5m2m9 | 5.2.2 | 31 | 8 | 238 | 0 | 23 | 0 | 269 | 0.0297 | 0.8848 | 0 | 0.0855 | 0 |
| 4 | August | 9 | aControl | adult | Bold | Bold | 1 | 8 | 4.8 | 4m8m9 | 4.1.8 | 33 | 42 | 131 | 0 | 31 | 18 | 222 | 0.1892 | 0.5901 | 0 | 0.1396 | 0.0811 |
| 4 | August | 9 | aControl | adult | Bold | Bold | 2 | 8 | 4.8 | 4m8m9 | 4.2.8 | 33 | 22 | 112 | 5 | 34 | 49 | 222 | 0.0991 | 0.5045 | 0.0225 | 0.1532 | 0.2207 |
| 5 | Septembe | 8 | Dog | adult | Shy | Bold | 1 | 2 | 5.2 | 5m2m8 | 5.1.2 | 33 | 11 | 77 | 0 | 6 | 110 | 204 | 0.0539 | 0.3775 | 0 | 0.0294 | 0.5392 |
| 5 | Septembe | 8 | Dog | adult | Shy | Bold | 2 | 2 | 5.2 | 5m2m8 | 5.2.2 | 33 | 0 | 179 | 0 | 11 | 14 | 204 | 0 | 0.8775 | 0 | 0.0539 | 0.0686 |
| 1 | April | 8 | Feed | adult | Bold | Bold | 2 | 4 | 1.4 | 1m4m8 | 1.2.4 | 34 | 64 | 144 | 0 | 34 | 27 | 269 | 0.2379 | 0.5353 | 0 | 0.1264 | 0.1004 |
| 4 | August | 12 | aControl | adult | Bold | Bold | 1 | 8 | 4.8 | 4m8m12 | 4.1.8 | 34 | 5 | 124 | 0 | 17 | 76 | 222 | 0.0225 | 0.5586 | 0 | 0.0766 | 0.3423 |
| 4 | August | 12 | aControl | adult | Bold | Bold | 2 | 8 | 4.8 | 4m8m12 | 4.2.8 | 34 | 3 | 128 | 1 | 7 | 83 | 222 | 0.0135 | 0.5766 | 0.0045 | 0.0315 | 0.3739 |
| 5 | Septembe | 9 | Dog | adult | Bold | Bold | 1 | 1 | 5.1 | 5m1m9 | 5.1.1 | 34 | 7 | 171 | 1 | 29 | 27 | 235 | 0.0298 | 0.7277 | 0.0043 | 0.1234 | 0.1149 |
| 5 | Septembe | 9 | Dog | adult | Bold | Bold | 2 | 1 | 5.1 | 5m1m9 | 5.2.1 | 34 | 1 | 0 | 0 | 0 | 234 | 235 | 0.0043 | 0 | 0 | 0 | 0.9957 |
| 5 | Septembe | 8 | Dog | adult | Bold | Bold | 1 | 1 | 5.1 | 5m1m8 | 5.1.1 | 36 | 0 | 180 | 4 | 38 | 25 | 247 | 0 | 0.7287 | 0.0162 | 0.1538 | 0.1012 |
| 5 | Septembe | 8 | Dog | adult | Bold | Bold | 2 | 1 | 5.1 | 5m1m8 | 5.2.1 | 36 | 1 | 0 | 0 | 0 | 246 | 247 | 0.004 | 0 | 0 | 0 | 0.996 |
| 4 | August | 8 | Feed | adult | Bold | Bold | 1 | 3 | 4.3 | 4m3m8 | 4.1.3 | 37 | 21 | 120 | 27 | 1 | 51 | 220 | 0.0955 | 0.5455 | 0.1227 | 0.0045 | 0.2318 |
| 4 | August | 8 | Feed | adult | Bold | Bold | 2 | 3 | 4.3 | 4m3m8 | 4.2.3 | 37 | 9 | 97 | 28 | 7 | 79 | 220 | 0.0409 | 0.4409 | 0.1273 | 0.0318 | 0.3591 |
| 4 | August | 8 | aControl | adult | Bold | Bold | 1 | 8 | 4.8 | 4m8m8 | 4.1.8 | 45 | 36 | 163 | 0 | 6 | 26 | 231 | 0.1558 | 0.7056 | 0 | 0.026 | 0.1126 |
| 4 | August | 8 | aControl | adult | Bold | Bold | 2 | 8 | 4.8 | 4m8m8 | 4.2.8 | 45 | 23 | 118 | 25 | 12 | 53 | 231 | 0.0996 | 0.5108 | 0.1082 | 0.0519 | 0.2294 |
| 2 | June | 10 | aChildaControl | child | Bold | Bold | 1 | 8 | 2.8 | 2m8m10 | 2.1.8 | 48 | 0 | 259 | 0 | 0 | 13 | 272 | 0 | 0.9522 | 0 | 0 | 0.0478 |
| 2 | June | 10 | aChildaControl | child | Bold | Bold | 2 | 8 | 2.8 | 2m8m10 | 2.2.8 | 48 | 18 | 186 | 13 | 48 | 7 | 272 | 0.0662 | 0.6838 | 0.0478 | 0.1765 | 0.0257 |
| 2 | June | 8 | aChildaControl | child | Bold | Bold | 1 | 8 | 2.8 | 2m8m8 | 2.1.8 | 54 | 11 | 224 | 0 | 0 | 0 | 235 | 0.0468 | 0.9532 | 0 | 0 | 0 |
| 2 | June | 8 | aChildaControl | child | Bold | Bold | 2 | 8 | 2.8 | 2m8m8 | 2.2.8 | 54 | 42 | 127 | 0 | 32 | 34 | 235 | 0.1787 | 0.5404 | 0 | 0.1362 | 0.1447 |
